# Supplementary material for: Electroredox carbene organocatalysis with iodide as promoter
Source: Nat Commun. 2022 Jul 2;13:3827. doi: 10.1038/s41467-022-31453-7 (PMC9250514; doi:10.1038/s41467-022-31453-7)
Supplement: Supplementary file 1 — Supplementary Information [file 41467_2022_31453_MOESM1_ESM.pdf]

Supplementary Information

Electroredox Carbene Organocatalysis with Iodide as Promoter

*Peng Zhou, Wenchang Li, Jianyong Lan, and Tingshun Zhu**

School of chemistry, Sun Yat-sen University, Guangzhou 510275, China;

zhutshun@mail.sysu.edu.cn

List of Contents

1. Supplementary Tables 2
2. Supplementary Methods 8
   2.1 General information 8
   2.2 General produce for electrochemical strategy by the catalysis of NHC 9
   2.3 Radical clock experiments 14
   2.4 Cyclic Voltammetry 16
   2.5 Characterization Data 17
3. Supplementary Figures 32
   3.1 Cyclic Voltammetry 32
   3.2 NMR spectra 35
   3.3 UPCC Spectra 83
4. Supplementary Reference 115

1. Supplementary Tables

Supplementary Table 1. Condition evaluation for electrochemical [4+2] annulation of β-methyl enals (1) with hydrazones (2) by the catalysis of NHC A.

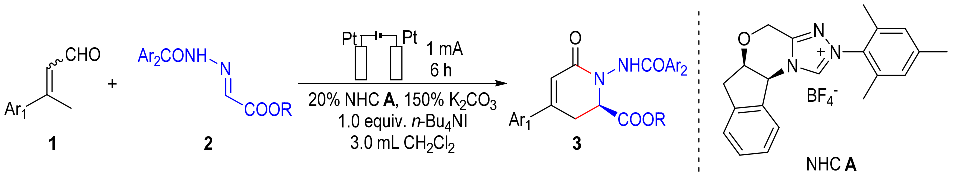

entry	deviation	yield (%)[a]	ee (%)[b]
1	None	79	97
2	No NHC **A**	0	-
3	No electricity	n.r.	-
4	DCE as solvent	60	95
5	THF as solvent	64	96
6	CH_3CN as solvent	30	93
7	DMF as solvent	14	85
8	DMSO as solvent	10	81
9	*n*-Bu_4NBF_4 as electrolyte	trace	-
10	*n*-Bu_4NBr as electrolyte	30	95
11	Et_4NI as electrolyte	68	95
12	Cs_2CO_3 as base	29	90
13	DBU as base	29	31
14	DMAP as base	26	97
15	DIEA as base	67	97
16	Graphite as anode	62	97

[a]Yield of the isolated product. [b]The enantiomeric ratio (ee) was determined by chiral stationary HPLC.

Supplementary Table 2. Condition evaluation for electrochemical [3+3] annulation of α,β-unsaturated aldehydes (4) with 1,3-dicarbonyl derivatives (5) by the catalysis of NHC B

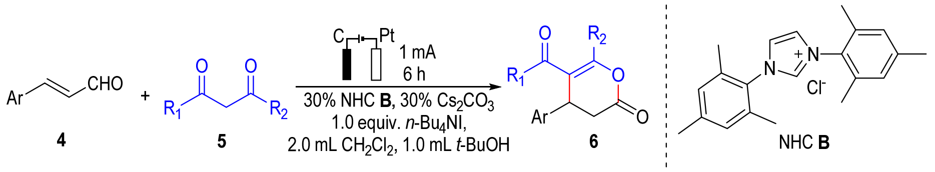

entry	4/5 ratio	deviation	yield (%)[a]
1	0.1 mmol/0.1 mmol	CH_3CN as solvent	43
2	0.1 mmol/0.1 mmol	DCE as solvent	30
3	0.1 mmol/0.1 mmol	CH_2Cl_2 as solvent	57
4	0.1 mmol/0.1 mmol	CH_2Cl_2/*t*-BuOH (2.5/0.5) as solvent	62
5	0.1 mmol/0.1 mmol	none	66
6	0.1 mmol/0.1 mmol	CH_3CN/*t*-BuOH (1.5/1.5) as solvent	58
7	0.1 mmol/0.2 mmol	none	73

[a]Yield of the isolated product.

Supplementary Table 3. Condition evaluation for electrochemical [3+3] annulation of α,β-unsaturated aldehydes (4) with 1,3-dicarbonyl derivatives (5) by the catalysis of NHC C.

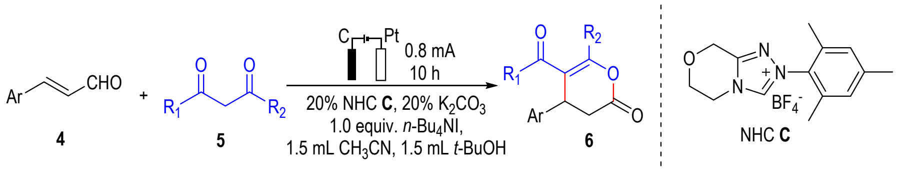

entry	Current	deviation	yield (%)[a]
1	1 mA, 6 h	CH_3CN as solvent	40
2	1 mA, 6 h	CH_2Cl_2 as solvent	24
3	1 mA, 6 h	CH_3CN/*t*-BuOH (2/1) as solvent	37
4	1 mA, 6 h	none	67
5	0.8 mA, 10 h	none	73
6	0.8 mA, 10 h	DBU as base	54
7	0.8 mA, 10 h	DABCO as base	62
8	0.8 mA, 10 h	DMAP as base	50

[a]Yield of the isolated product.

Supplementary Table 4. Condition evaluation for electrochemical [2+4] annulation of aldehydes (7) with enones (8) by the catalysis of NHC A.

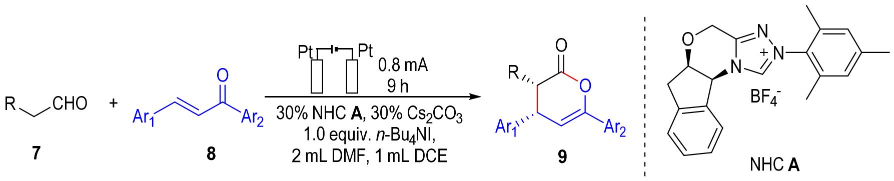

entry	deviation	yield (%)[a]	dr[b]	ee (%)[c]
1	None	81	14:1	99
2	No NHC **A**	0	-	-
3	No electricity	n.r.	-	-
4	DMF as the solvent	92	2:1	99
5	DCE as the solvent	6	>20:1	99
6	DMF/DCE (1:2) as the solvent	47	19:1	99
7	Bu_4NBF_4 as the electrolyte	7	2:1	94
8	Bu_4NBr as the electrolyte	27	>20:1	97
9	NHC **A** (20%)	62	12:1	99
10	Graphite as the anode	55	8:1	99

[a]The yield was determined by ^1H NMR using CH_2Br_2 as an internal standard. [b]The dr was determined by ^1H NMR analysis of the crude products. [c]The ee was determined by chiral stationary HPLC.

Supplementary Table 5. Condition evaluation for electrochemical asymmetric acylation of hydroxyphthalide by carbene-catalyzed dynamic kinetic resolution.

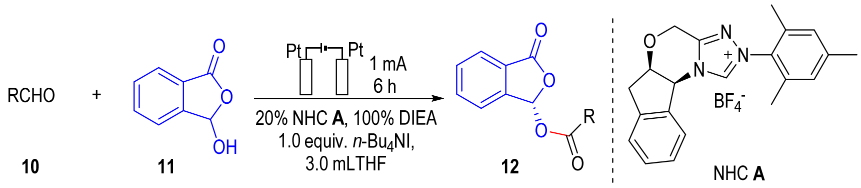

entry	**10/11** ratio	deviation	yield (%)[a]	ee (%)[b]
1	0.1 mmol/0.12 mmol	Cs_2CO_3 as base	18	65
2	0.1 mmol/0.12 mmol	DABCO as base	76	85
3	0.1 mmol/0.12 mmol	DBU as base	58	84
4	0.1 mmol/0.12 mmol	130% DIEA as base	36	90
5	0.1 mmol/0.12 mmol	CH_3CN as solvent	35	80
6	0.1 mmol/0.12 mmol	DMF as solvent	64	70
7	0.1 mmol/0.12 mmol	Bu_4NBr as electrolyte	trace	-
8	0.1 mmol/0.12 mmol	none	47	93
9	0.15 mmol/0.1 mmol	none	66	95
10	0.18 mmol/0.1 mmol	none	84	95

[a]Yield of the isolated product. [b]The enantiomeric ratio (ee) was determined by chiral stationary HPLC.

Supplementary Table 6. Condition evaluation for electrochemical [4+2] annulation of aldehyde (13) and 1,3-diketone (5a) by the catalysis of NHC B

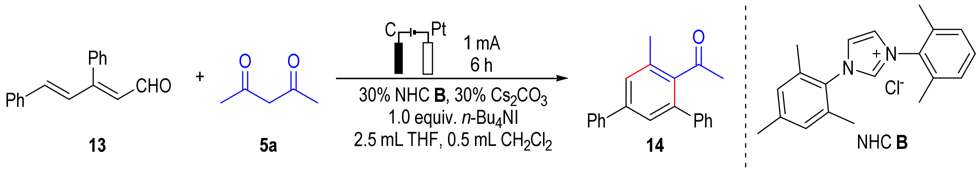

entry	deviation	yield (%)[a]
`1	THF as solvent	46
2	DCE as solvent	16
3	none	48
4	DMAP as base	0
5	DABCO as base	0
6	K_2CO_3 as base	trace
7	Pt as anode	42

[a]Yield of the isolated product.

Supplementary Table 7. Condition evaluation for electrochemical [3+3] annulation of cinnamaldehyde (4a) and imine (15) by the catalysis of NHC A

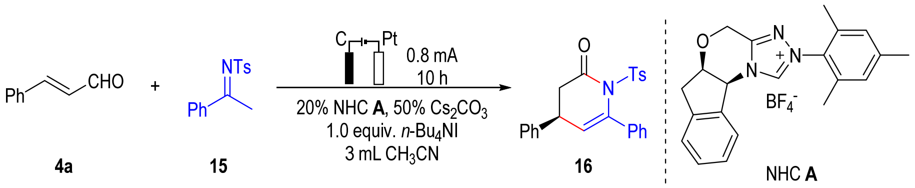

entry	Current	deviation	yield (%)[a]	ee (%)[b]
1	0.8 mA, 9 h	DMF as solvent	29	97
2	0.8 mA, 9 h	CH_2Cl_2 as solvent	20	97
3	0.8 mA, 9 h	30% Cs_2CO_3 as base	33	97
4	0.8 mA, 9 h	none	48	97
5	0.8 mA, 9 h	75% Cs_2CO_3 as base	31	97
6	0.8 mA, 9 h	100% Cs_2CO_3 as base	19	97
7	0.8 mA, 10 h	none	55	97

[a]Yield of the isolated product. [b]The enantiomeric ratio (ee) was determined by chiral stationary HPLC.

Supplementary Table 8. Condition evaluation for electrochemical [4+2] annulation of enal (1a) and imine (17) by the catalysis of NHC A

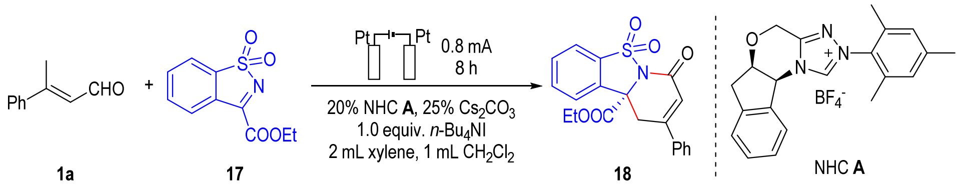

entry	Current	anode	deviation	yield (%)[a]	ee (%)[b]
1	1 mA, 6 h	graphite	CH_2Cl_2 as solvent	61	44
2	1 mA, 6 h	graphite	none	45	88
3	1 mA, 6 h	graphite	CH_2Cl_2/xylene (2/1) as solvent	55	74
4	1 mA, 6 h	Pt	none	53	86
5	0.8 mA, 8 h	Pt	none	51	88

[a]Yield of the isolated product. [b]The enantiomeric ratio (ee) was determined by chiral stationary HPLC.

2. Supplementary Methods

2.1 General information

Unless otherwise stated, all reagent-grade chemicals were obtained from commercial suppliers and were used as received without further purification. CH_2Cl_2 and acetonitrile were distilled from CaH_2 immediately prior to use. THF used in reactions was freshly distilled from sodium. The other solvents used in the experiments were all purchased anhydrous solvents and used directly. All reactions were conducted using an IKA Electrasyn 2.0 using 5 mL vials for reaction optimization. Unless otherwise noted, all reactions were carried out under an atmosphere of air. Analytical thin layer chromatography was performed on 0.20 mm silica gel plates and visualized under 254 nm UV light. Flash column chromatography was performed using silica gel (200-300 mesh). Electrochemical experiments were performed with Electrasyn 2.0 (IKA). Pt electrode were purchased in Baoji Zhiming Company as titanium electrode coated with Pt.

^{1}H NMR, ^{13}C NMR and ^{19}F NMR spectra were measured on a 400 MHz Bruker AVANCE III spectrometer (400 MHz for ^{1}H, 100 MHz for ^{13}C and 376 MHz for ^{19}F), using DMSO-d_6 or $CDCl_3$ as the solvent with tetramethylsilane (TMS) as the internal standard at ambient temperature. Chemical shifts were reported in ppm. ^{1}H NMR spectra were referenced to $CDCl_3$ (7.26 ppm) or DMSO-d_6 (2.50 ppm). Peak multiplicities were designated by the following abbreviations: s, singlet; d, doublet; t, triplet; q, quartet; dd, doublet of doublets; m, multiplets and etc. Chemical shifts are given in δ relative to TMS, the coupling constants J are given in Hz. High resolution mass spectra of new compounds were recorded on MAT 95XP (Thermo, EI) Infrared (IR) spectra were recorded on PerkinElmer Frontier spectrometer and reported in wave numbers (cm^{-1}).

The enantiomeric excesses were determined with Waters Acquity Ultra Performance Convergence Chromatography (UPCC) and chiral stationary phases column was used Chiralcel OX-3, OD-3, Chiralpak AD-3 columns from Daicel Chiral Technologies and Trefoil CEL2 column from Waters. The cyclic voltammetry was carried out with a Metrohm Autolab M204 workstation. High resolution mass spectra of new compounds were recorded on LTQ Orbitrap Elite LC/MS (ESI or APCI).

2.2 General produce for electrochemical strategy by the catalysis of NHC

General produce for electrochemical [4+2] annulation of β-methyl enals (1) with hydrazones (2) by the catalysis of NHC A.

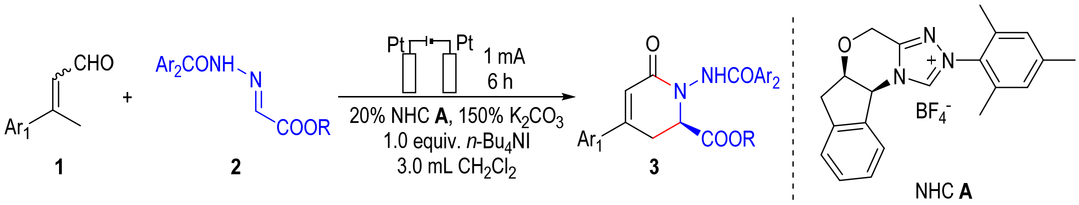

The ElectraSyn vial (5 mL) with a stir bar was charged with β-methyl enals **1** (0.15 mmol, 1.5 equiv.), NHC **A** (0.02 mmol, 20%), K_2CO_3 (0.15 mmol, 1.5 equiv.), *n*-Bu_4NI (0.1 mmol, 1.0 equiv.) and hydrazones **2** (0.1 mmol, 1.0 equiv.) followed by anhydrous CH_2Cl_2 (3.0 mL). The ElectraSyn vial cap equipped with anode (Pt) and cathode (Pt) were inserted into the mixture. After pre-stirring for 2 minutes, the Electrasyn vial was connected to the Electrasyn 2.0 and the reaction mixture was electrolyzed under a constant current of 1.0 mA for a total reaction time of 6 hours for a total of 2.24 F/mol of charge accompanied by magnetic stirring. The ElectraSyn vial cap was removed, and electrodes were rinsed with EtOAc, which was combined with the crude mixture. After concentrated under reduced pressure, the crude residue was purified via flash column chromatography to afford the desired product **3**.

General produce for electrochemical [3+3] annulation of α,β-unsaturated aldehydes (4) with 1,3-dicarbonyl derivatives (5) by the catalysis of NHC B.

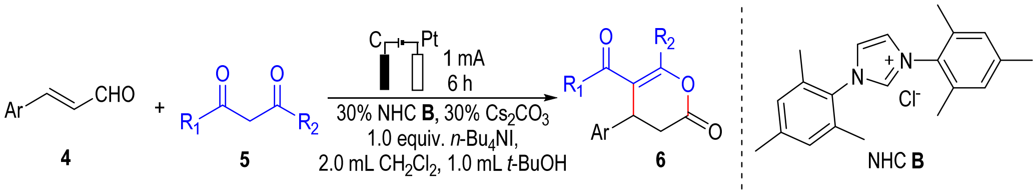

The ElectraSyn vial (5 mL) with a stir bar was charged with α,β-unsaturated aldehydes **4** (0.1 mmol, 1.0 equiv.), NHC **B** (0.03 mmol, 30%), Cs_2CO_3 (0.03 mmol, 30%.), *n*-Bu_4NI (0.1 mmol, 1.0 equiv.) and 1,3-dicarbonyl derivatives **5** (0.2 mmol, 2.0 equiv.) followed by anhydrous CH_2Cl_2 (2.0 mL) and *t*-BuOH (1.0 mL). The ElectraSyn vial cap equipped with anode (graphite) and cathode (Pt) were inserted into the mixture. After pre-stirring for 2 minutes, the Electrasyn vial was connected to the Electrasyn 2.0 and the reaction mixture was electrolyzed under a constant current of 1.0 mA for a total reaction time of 6 hours for a total of 2.24 F/mol of charge accompanied by magnetic stirring. The ElectraSyn vial cap was removed, and electrodes were rinsed with EtOAc, which was combined with the crude mixture. After concentrated under reduced pressure, the crude residue was purified via flash column chromatography to afford the desired product **6**.

General produce for electrochemical [3+3] annulation of α,β-unsaturated aldehydes (4) with 1,3-dicarbonyl derivatives (5) by the catalysis of NHC C.

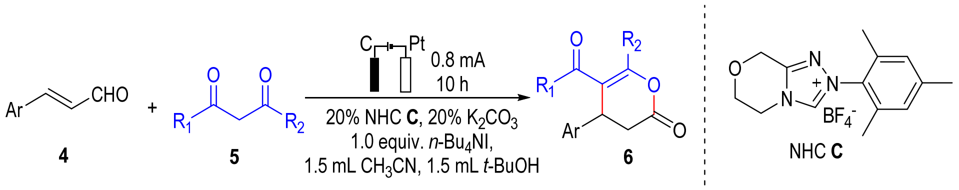

The ElectraSyn vial (5 mL) with a stir bar was charged with α,β-unsaturated aldehydes **4** (0.1 mmol, 1.0 equiv.), NHC **C** (0.02 mmol, 20%), K_2CO_3 (0.02 mmol, 20%.), *n*-Bu_4NI (0.1 mmol, 1.0 equiv.) and 1,3-dicarbonyl derivatives **5** (0.2 mmol, 2.0 equiv.) followed by anhydrous CH_3CN (1.5 mL) and *t*-BuOH (1.5 mL). The ElectraSyn vial cap equipped with anode (graphite) and cathode (Pt) were inserted into the mixture. After pre-stirring for 2 minutes, the Electrasyn vial was connected to the Electrasyn 2.0 and the reaction mixture was electrolyzed under a constant current of 0.8 mA for a total reaction time of 10 hours for a total of 2.98 F/mol of charge accompanied by magnetic stirring. The ElectraSyn vial cap was removed, and electrodes were rinsed with EtOAc, which was combined with the crude mixture. After concentrated under reduced pressure, the crude residue was purified via flash column chromatography to afford the desired product **6**.

General produce for electrochemical [2+4] annulation of aldehydes (7) with enones (8) by the catalysis of NHC A.

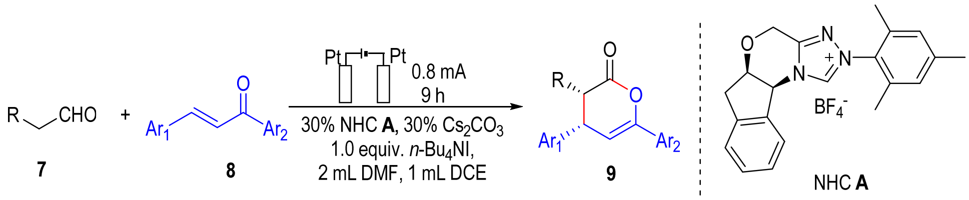

The ElectraSyn vial (5 mL) with a stir bar was charged with enones **8** (0.1 mmol, 1.0 equiv.), NHC **A** (0.03 mmol, 30%), Cs_2CO_3 (0.03 mmol, 30%.), *n*-Bu_4NI (0.1 mmol, 1.0 equiv.) and aldehydes **7** (0.25 mmol, 2.5 equiv.) followed by anhydrous DMF (2.0 mL) and DCE (1.0 mL). The ElectraSyn vial cap equipped with anode (Pt) and cathode (Pt) were inserted into the mixture. After pre-stirring for 2 minutes, the Electrasyn vial was connected to the Electrasyn 2.0 and the reaction mixture was electrolyzed under a constant current of 0.8 mA for a total reaction time of 9 hours for a total of 2.68 F/mol of charge accompanied by magnetic stirring. The ElectraSyn vial cap was removed, and electrodes were rinsed with EtOAc, which was combined with the crude mixture, and extracted with H_2O three times. After concentrated the organic phase under reduced pressure, the crude residue was purified via flash column chromatography to afford the desired product **9**.

General produce for electrochemical asymmetric acylation of hydroxyphthalide by carbene-catalyzed dynamic kinetic resolution.

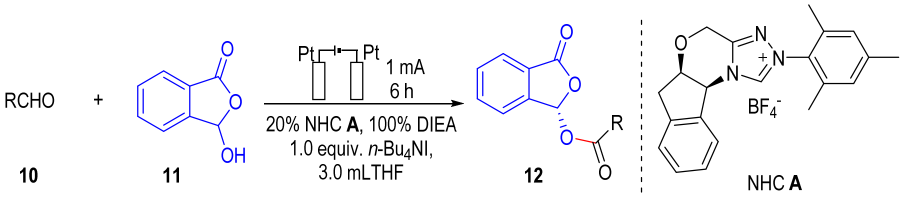

The ElectraSyn vial (5 mL) with a stir bar was charged with aldehydes **10** (0.18 mmol, 1.8 equiv.), NHC **A** (0.02 mmol, 20%), DIEA (0.1 mmol, 100%.), *n*-Bu_4NI (0.1 mmol, 1.0 equiv.) and hydroxyphthalide **11** (0.1 mmol, 1.0 equiv.) followed by anhydrous THF (3.0 mL). The ElectraSyn vial cap equipped with anode (Pt) and cathode (Pt) were inserted into the mixture. After pre-stirring for 2 minutes, the Electrasyn vial was connected to the Electrasyn 2.0 and the reaction mixture was electrolyzed under a constant current of 1 mA for a total reaction time of 6 hours for a total of 2.24 F/mol of charge accompanied by magnetic stirring. The ElectraSyn vial cap was removed, and electrodes were rinsed with EtOAc, which was combined with the crude mixture. After concentrated under reduced pressure, the crude residue was purified via flash column chromatography to afford the desired product **12**.

General produce for electrochemical [4+2] annulation of aldehyde (13) and 1,3-diketone (5a) by the catalysis of NHC B

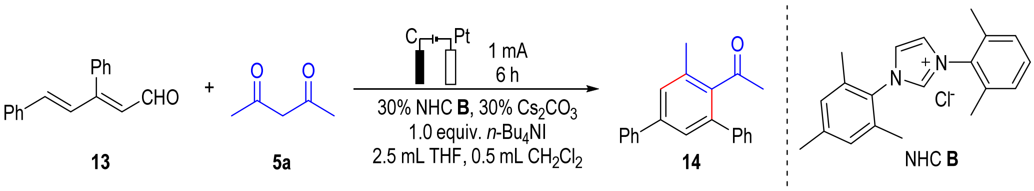

The ElectraSyn vial (5 mL) with a stir bar was charged with aldehydes **13** (0.10 mmol, 1.0 equiv.), NHC **B** (0.03 mmol, 30%), Cs_2CO_3 (0.03 mmol, 30%.), *n*-Bu_4NI (0.1 mmol, 1.0 equiv.) and 1,3-diketone **5a** (0.1 mmol, 1.0 equiv.) followed by anhydrous THF (2.5 mL) and CH_2Cl_2 (0.5 mL). The ElectraSyn vial cap equipped with anode (graphite) and cathode (Pt) were inserted into the mixture. After pre-stirring for 2 minutes, the Electrasyn vial was connected to the Electrasyn 2.0 and the reaction mixture was electrolyzed under a constant current of 1 mA for a total reaction time of 6 hours for a total of 2.24 F/mol of charge accompanied by magnetic stirring. The ElectraSyn vial cap was removed, and electrodes were rinsed with EtOAc, which was combined with the crude mixture. After concentrated under reduced pressure, the crude residue was purified via flash column chromatography to afford the desired product **14**.

General produce for electrochemical [3+3] annulation of cinnamaldehyde (4a) and imine (15) by the catalysis of NHC A

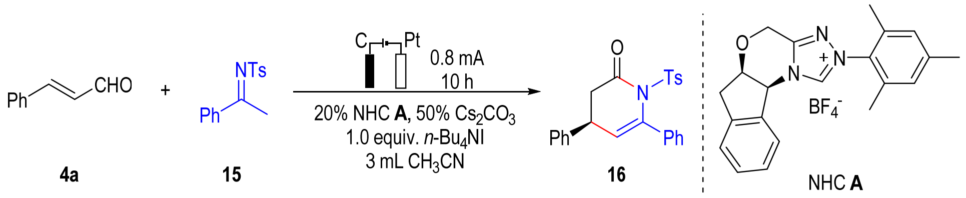

The ElectraSyn vial (5 mL) with a stir bar was charged with cinnamaldehyde **4a** (0.10 mmol, 1.0 equiv.), NHC **A** (0.02 mmol, 20%), Cs_2CO_3 (0.05 mmol, 50%.), *n*-Bu_4NI (0.1 mmol, 1.0 equiv.) and imine **15** (0.2 mmol, 2.0 equiv.) followed by anhydrous CH_3CN (3 mL). The ElectraSyn vial cap equipped with anode (graphite) and cathode (Pt) were inserted into the mixture. After pre-stirring for 2 minutes, the Electrasyn vial was connected to the Electrasyn 2.0 and the reaction mixture was electrolyzed under a constant current of 0.8 mA for a total reaction time of 10 hours for a total of 2.98 F/mol of charge accompanied by magnetic stirring. The ElectraSyn vial cap was removed, and electrodes were rinsed with EtOAc, which was combined with the crude mixture. After concentrated under reduced pressure, the crude residue was purified via flash column chromatography to afford the desired product **16**.

General produce for electrochemical [4+2] annulation of enal (1a) and imine (17) by the catalysis of NHC A

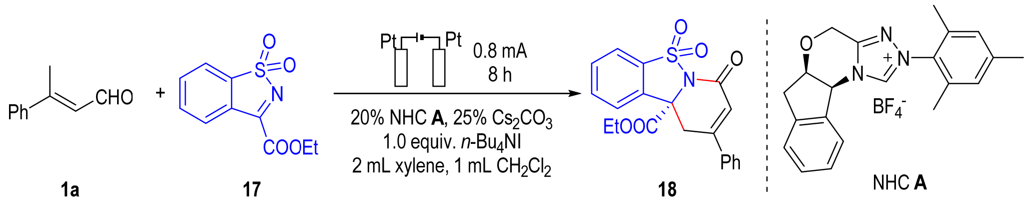

The ElectraSyn vial (5 mL) with a stir bar was charged with enal **1a** (0.15 mmol, 1.5 equiv.), NHC **A** (0.02 mmol, 20%), Cs_2CO_3 (0.025 mmol, 25%.), *n*-Bu_4NI (0.1 mmol, 1.0 equiv.) and imine **17** (0.1 mmol, 1.0 equiv.) followed by anhydrous xylene (2 mL) and CH_2Cl_2 (1 mL). The ElectraSyn vial cap equipped with anode (Pt) and cathode (Pt) were inserted into the mixture. After pre-stirring for 2 minutes, the Electrasyn vial was connected to the Electrasyn 2.0 and the reaction mixture was electrolyzed under a constant current of 0.8 mA for a total reaction time of 8 hours for a total of 2.39 F/mol of charge accompanied by magnetic stirring. The ElectraSyn vial cap was removed, and electrodes were rinsed with EtOAc, which was combined with the crude mixture. After concentrated under reduced pressure, the crude residue was purified via flash column chromatography to afford the desired product **18**.

Gram-scale synthesis of 3a

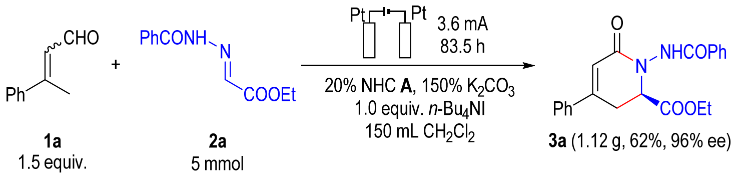

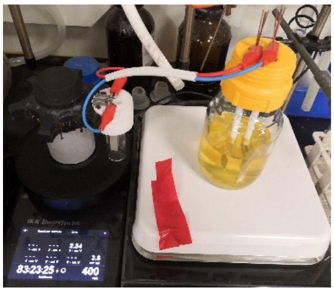

To a 250 mL clean and dry glass chamber charged with a proper stir bar was added **1a** (1.5 equiv.), **2a** (5 mmol), NHC **A** (20%, 1mmol), K_2CO_3 (1.5 equiv.), Bu_4NI (1.0 equiv.) and CH_2Cl_2 (150 mL). The glass chamber was sealed with a septum equipped with two Pt electrodes (2.0 × 2.0 cm^2) and the reaction mixture was electrolyzed under a constant current of 3.6 mA for 83.5 h. After the reaction was completed, the solvent removed under reduced pressure. The crude product was purified by flash column chromatography affording the desired product **3a** in 62% (1.12 g) isolated yield (96% ee).

The synthesis of D and E

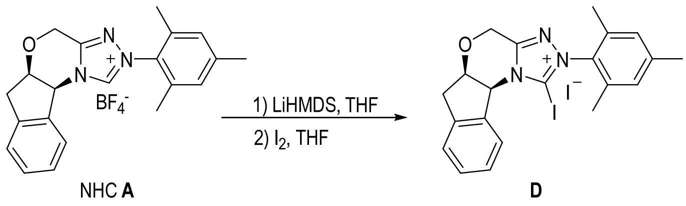

To a stirred the solution of NHC **A** (0.5 mmol, 1.0 equiv) in anhydrous THF (4.0 mL) was slowly added LiHMDS (0.55 mmol, 1.1 equiv.) in a flame-dried Schlenk tube under N_2. The resultant mixture was stirred at room temperature for 2 h followed by the addition of I_2 solution (0.6 mmol, 1.2 equiv. in 2 mL THF) carefully and the color of the iodine solution must not be persistent upon addition. The reaction mixture was stirred at room temperature for further 8 h under N_2. The solvent was removed under reduced pressure and the residue was purified by silica gel column chromatography to afford (5a*R*,10b*S*)-1-iodo-2-mesityl-5a,10b-dihydro-4H,6H-indeno[2,1-b][1,2,4]triazolo[4,3-d][1,4]oxazin-2-ium iodide (**D**).

1,3-bis(2,4,6-trimethylphenyl)-2-iodoimidazolium iodide (**E**) was produced in the same way but replace NHC **A** with NHC **B**.

2.3 Radical clock experiments

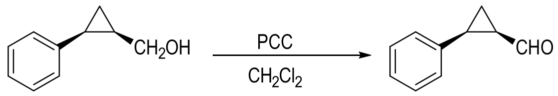

cis-**20**-S *cis*-**20**

Allylic alcohol *cis*-**20**-S was synthesized following a two-steps procedure from styrene and Ethyl diazoacetate, as described in the literature [1](ref).

To a solution of *cis*-**20**-S (1.0 equiv.) in DCM (0.5 M) was added PCC (1.5 equiv.) portion wise at rt. After overnight stirring at rt, the reaction mixture was filtered through a pad of celite and the filtrate was concentrated in vacuo. The residue was purified by column chromatography on silica gel to afford product *cis*-**20** as described in the literature.[1]

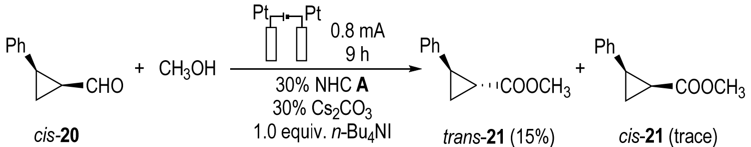

cis-**20** 0.1 mmol; 0.2 mmol; *trans*-**21** (15%); *cis*-**21** (trace)

The ElectraSyn vial (5 mL) with a stir bar was charged with CH_3OH (0.2 mmol, 2.0 equiv.), NHC **A** (0.03 mmol, 30%), Cs_2CO_3 (0.03 mmol, 30%.), *n*-Bu_4NI (0.1 mmol, 1.0 equiv.) and *cis*-**20** (0.1 mmol) followed by anhydrous DMF (2.0 mL) and DCE (1.0 mL). The ElectraSyn vial cap equipped with anode (Pt) and cathode (Pt) were inserted into the mixture. After pre-stirring for 2 minutes, the Electrasyn vial was connected to the Electrasyn 2.0 and the reaction mixture was electrolyzed under a constant current of 0.8 mA for a total reaction time of 9 hours accompanied by magnetic stirring. The ElectraSyn vial cap was removed, and electrodes were rinsed with EtOAc, which was combined with the crude mixture, and extracted with H_2O three times. After concentrated the organic phase under reduced pressure, the crude residue was purified via flash column chromatography to afford *trans*-**21** as a pale yellow oil (15%), according with the description of the literature.[2]

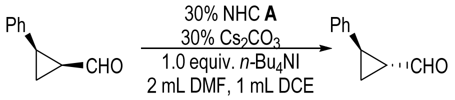

cis-**20** *trans*-**20** (0%)

The ElectraSyn vial (5 mL) with a stir bar was charged with NHC **A** (0.03 mmol, 30%), Cs_2CO_3 (0.03 mmol, 30%.), *n*-Bu_4NI (0.1 mmol, 1.0 equiv.) and *cis*-**20** (0.1 mmol) followed by anhydrous DMF (2.0 mL) and DCE (1.0 mL). The ElectraSyn vial cap equipped with anode (Pt) and cathode (Pt) were inserted into the mixture. The Electrasyn vial was for 9 hours accompanied by magnetic stirring. The ElectraSyn vial cap was removed, and electrodes were rinsed with EtOAc, which was combined

with the crude mixture, and extracted with H_2O three times. After concentrated the organic phase under reduced pressure, the crude residue was tested by ^1H NMR which shows that there is not the formation of *trans*-**20**.

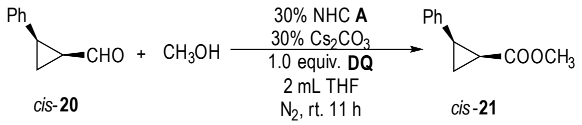

To a flame-dried Schlenk tube equipped with a magnetic stirrer bar was charged with *cis*-**20** (0.2 mmol), NHC **A** (30%), Cs_2CO_3 (30%), **DQ** (1.0 equiv.) under air, then the vessel was evacuated and re-filled with N_2 for four times. CH_3OH (2 equiv.) and anhydrous THF (2.0 mL) were added under N_2 counter flow. The Schlenk tube was sealed and the reaction mixture was stirred at the room temperature for 11 h. The crude mixture was tested by ^1H NMR (red line) and purified by column chromatography to provide 92% product *cis*-**21** exclusively.

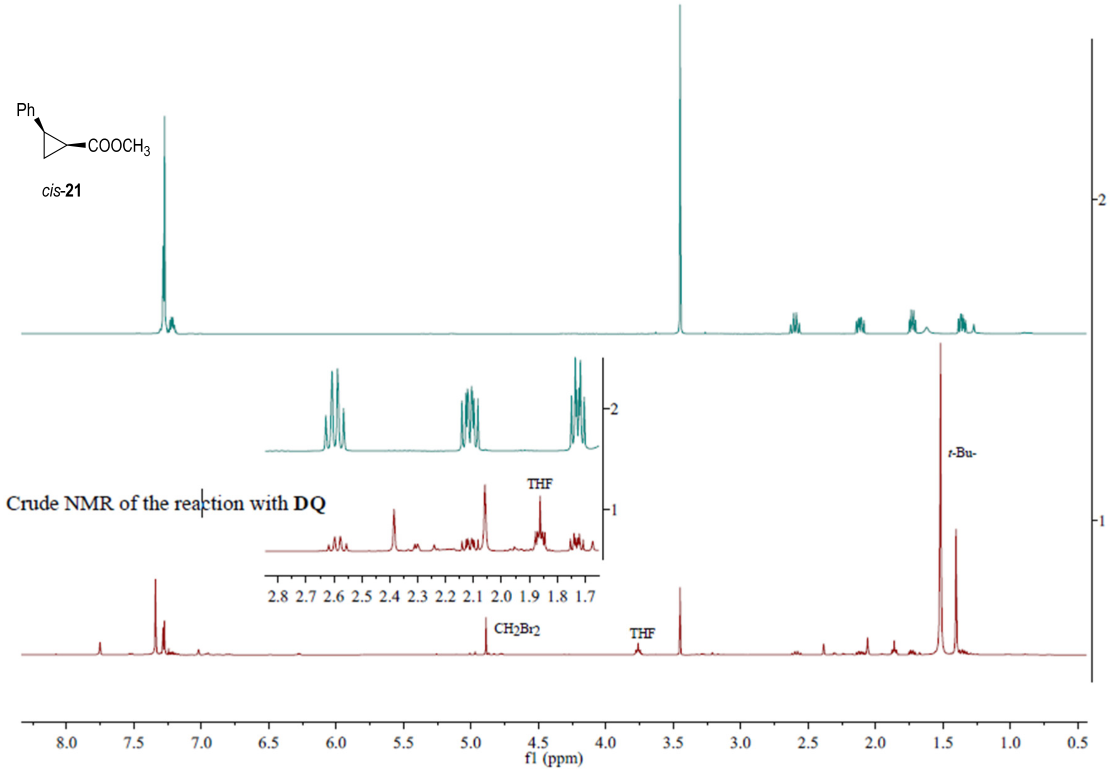

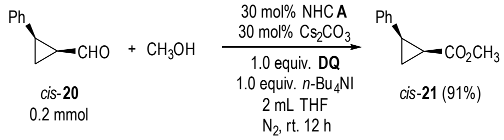

To a flame-dried Schlenk tube equipped with a magnetic stirrer bar was charged with *cis*-**20** (0.2 mmol), NHC **A** (30%), Cs_2CO_3 (30%), n-Bu_4NI (1.0 equiv.), **DQ** (1.0 equiv.) under air, then the vessel was evacuated and re-filled with N_2 for four times. CH_3OH (2 equiv.) and anhydrous THF (2.0 mL) were added under N_2 counter flow. The Schlenk tube was sealed and the reaction mixture was stirred at the room temperature for 12 h. The crude mixture was tested by ^1H NMR (red line) and purified by column chromatography to provide 91% product *cis*-**21** exclusively.

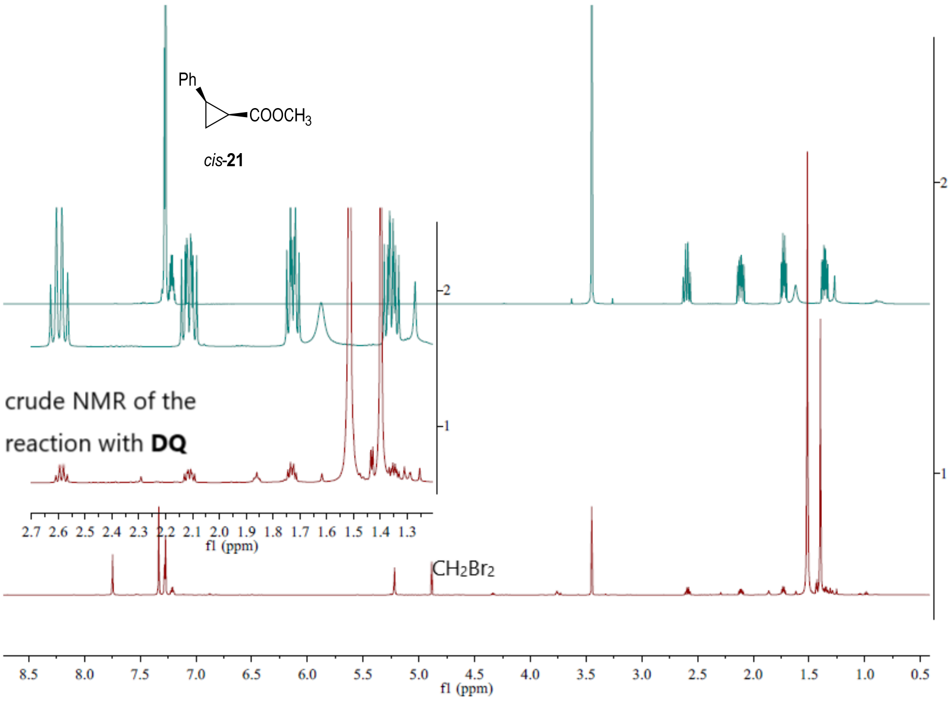

2.4 Cyclic Voltammetry

Cyclic voltammograms were recorded with a Metrohm Autolab M204 workstation at room temperature. *n*-Bu_4NBF_4 (0.1 M) was used as the supporting electrolyte, and a Pt electrode (diameter 1 mm, length 1 cm) was used as the working electrode. The counter electrode was a Pt electrode (diameter 1 mm, length 1 cm) and the Ag/$AgNO_3$ electrode (0.05 M $AgNO_3$ and 0.1 M *n*-Bu_4NBF_4 in CH_3CN) was utilized as a quasi-reference electrode. After each set of scans, the redox potential of ferrocene was added to measured and used to provide an internal reference. Then the potential values were the adjusted relative to SCE ($E°(Fc/Fc^+)$ = 0.424 V vs SCE[3]). The scan rate was 50 $mV{\cdot}s^{-1}$.

2.5 Characterization Data

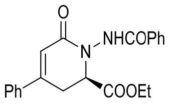

3a

Ethyl (*R*)-1-benzamido-6-oxo-4-phenyl-1,2,3,6-tetrahydropyridine-2-carboxylate (3a)[4]: white solid; ^{1}H NMR (400 MHz, $CDCl_3$) δ 9.05 (s, 1H), 7.85 (d, *J* = 7.5 Hz, 2H), 7.55 – 7.46 (m, 3H), 7.46 – 7.39 (m, 5H), 6.34 (d, *J* = 2.5 Hz, 1H), 4.79 (dd, *J* = 7.2, 2.3 Hz, 1H), 4.21 (q, *J* = 7.1 Hz, 2H), 3.60 (ddd, *J* = 17.6, 7.1, 2.6 Hz, 1H), 3.38 (dd, *J* = 17.6, 2.4 Hz, 1H), 1.23 (t, *J* = 7.1 Hz, 3H); ^{13}C NMR (100 MHz, $CDCl_3$) δ 170.8, 166.3, 164.3, 149.6, 136.7, 132.4, 132.0, 130.2, 128.9, 128.7, 127.5, 126.1, 118.5, 62.2, 61.2, 30.1, 14.2; UPCC analysis: 97% ee (Chiralcel OX-3, CO_2/CH_3OH = 85/15, 1.0 mL/min, detector: 290 nm), Rt (minor) = 9.3 min, Rt (major) = 30.3 min.

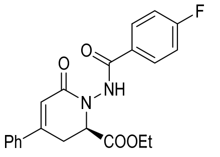

3b

Ethyl (*R*)-1-(4-fluorobenzamido)-6-oxo-4-phenyl-1,2,3,6-tetrahydropyridine-2-carboxylate (3b)[4]: white solid**;** ^{1}H NMR (400 MHz, $CDCl_3$) δ 9.15 (d, *J* = 8.1 Hz, 1H), 7.92 – 7.83 (m, 2H), 7.54 – 7.47 (m, 2H), 7.46 – 7.39 (m, 3H), 7.09 (t, *J* = 8.5 Hz, 2H), 6.34 (d, *J* = 2.5 Hz, 1H), 4.76 (d, *J* = 6.2 Hz, 1H), 4.22 (q, *J* = 7.1 Hz, 2H), 3.59 (dd, *J* = 17.5, 6.3 Hz, 1H), 3.38 (dd, *J* = 17.6, 2.3 Hz, 1H), 1.24 (t, *J* = 7.1 Hz, 3H); ^{13}C NMR (100 MHz, $CDCl_3$) δ 170.7, 165.2 (*J* = 251 Hz), 165.2, 164.3, 149.7, 136.7, 130.2, 130.0 (*J* = 9 Hz), 128.9, 128.1, 126.1, 118.5, 115.8 (*J* = 22 Hz), 62.2, 61.2, 30.2, 14.2; ^{19}F NMR (376 MHz, $CDCl_3$) δ 106.4; UPCC analysis: 96% ee (Chiralcel OX-3, CO_2/CH_3OH = 85/15, 1.0 mL/min, detector: 290 nm), Rt (minor) = 5.9 min, Rt (major) = 13.2 min.

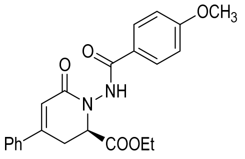

3c

Ethyl (*R*)-1-(4-methoxybenzamido)-6-oxo-4-phenyl-1,2,3,6-tetrahydropyridine-2-carboxylate (3c)[4]: white solid; ^{1}H NMR (400 MHz, $CDCl_3$) δ 8.78 (s, 1H), 7.83 (d, *J* = 8.4 Hz, 2H), 7.57 – 7.38 (m, 5H), 6.92 (d, *J* = 8.5 Hz, 2H), 6.34 (d, *J* = 2.4 Hz, 1H), 4.78 (d, *J* = 6.4 Hz, 1H), 4.22 (q, *J* = 7.1 Hz, 2H), 3.85 (s, 3H), 3.59 (dd, *J* = 17.4, 6.4 Hz, 1H), 3.37 (d, *J* = 17.4 Hz, 1H), 1.24 (t, *J* = 7.1 Hz, 3H); ^{13}C NMR (100 MHz, $CDCl_3$) δ 170.8, 165.9, 164.3, 162.9, 149.4, 136.8, 130.1, 129.4, 128.9, 126.1, 124.2, 118.6, 113.9, 62.2, 61.2, 55.5, 30.1, 14.2; UPCC analysis: 96% ee (Chiralcel OX-3, CO_2/CH_3OH = 85/15, 1.0 mL/min, detector: 290 nm), Rt (minor) = 14.9 min, Rt (major) = 35.4 min.

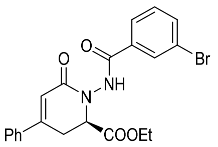

3d

Ethyl (*R*)-1-(3-bromobenzamido)-6-oxo-4-phenyl-1,2,3,6-tetrahydropyridine-2-carboxylate (3d)[4]: white solid; ^{1}H NMR (400 MHz, $CDCl_3$) δ 9.13 (s, 1H), 7.98 (s, 1H), 7.79 (d, *J* = 7.8 Hz, 1H), 7.65 (d, *J* = 8.0 Hz, 1H), 7.54 – 7.38 (m, 5H), 7.30 (t, *J* = 8.0 Hz, 1H), 6.33 (s, 1H), 4.76 (d, *J* = 6.9 Hz, 1H), 4.23 (q, *J* = 7.1 Hz, 2H), 3.59 (dd, *J* = 17.8, 7.0 Hz, 1H), 3.38 (d, *J* = 17.5 Hz, 1H), 1.25 (t, *J* = 7.1 Hz, 3H); ^{13}C NMR (100 MHz, $CDCl_3$) δ 170.7, 164.8, 164.3, 149.8, 136.7, 135.3, 133.8, 130.8, 130.3, 130.2, 129.0, 126.1, 126.0, 122.9, 118.4, 62.3, 61.1, 30.1, 14.2; UPCC analysis: 96% ee (Chiralcel OX-3, CO_2/CH_3OH = 85/15, 1.0 mL/min, detector: 290 nm), Rt (minor) = 10.0 min, Rt (major) = 23.1 min.

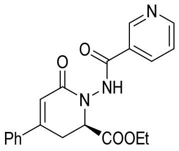

3e

Ethyl (*R*)-1-(nicotinamido)-6-oxo-4-phenyl-1,2,3,6-tetrahydropyridine-2-carboxylate (3e): white solid; ^{1}H NMR (400 MHz, $CDCl_3$) δ 9.33 (s, 1H), 9.07 (s, 1H), 8.76 (d, *J* = 4.7 Hz, 1H), 8.17 (d, *J* = 7.9 Hz, 1H), 7.56 – 7.47 (m, 2H), 7.46 – 7.35 (m, 4H), 6.34 (s, 1H), 4.76 (d, *J* = 6.9 Hz, 1H), 4.22 (q, *J* = 7.2 Hz, 2H), 3.60 (dd, *J* = 17.8, 7.0 Hz, 1H), 3.40 (d, *J* = 17.5 Hz, 1H), 1.24 (t, *J* = 7.0 Hz, 3H); ^{13}C NMR (100 MHz, $CDCl_3$) δ 170.6, 164.6, 164.3, 153.0, 149.9, 148.7, 136.6, 135.4, 130.3, 129.0, 127.8, 126.1, 123.5, 118.4, 62.3, 61.2, 30.2, 14.2; IR (film) ν_{max}: 3211, 2982, 1736, 1656, 1420, 1199, 1024, 764, 697; HRMS (ESI) for $C_{20}H_{20}N_3O_4^+$ ($[M+H]^+$): calcd 366.1448, found 366.1445; UPCC analysis: 95% ee (Chiralcel OX-3, CO_2/CH_3OH, 1.0 mL/min = 85/15, detector: 290 nm), Rt (minor) = 14.5 min, Rt (major) = 15.9 min.

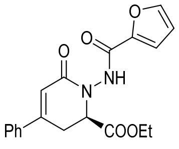

3f

Ethyl (*R*)-1-(furan-2-carboxamido)-6-oxo-4-phenyl-1,2,3,6-tetrahydropyridine-2-carboxylate (3f)[4]: white solid; ^{1}H NMR (400 MHz, $CDCl_3$) δ 8.77 (s, 1H), 7.54 – 7.46 (m, 3H), 7.46 – 7.39 (m, 3H), 7.22 (d, *J* = 3.5 Hz, 1H), 6.53 (dd, *J* = 3.5, 1.7 Hz, 1H), 6.34 (d, *J* = 2.5 Hz, 1H), 4.72 (dd, *J* = 7.1, 2.2 Hz, 1H), 4.22 (q, *J* = 7.1 Hz, 2H), 3.57 (ddd, *J* = 17.6, 7.1, 2.6 Hz, 1H), 3.37 (dd, *J* = 17.6, 2.3 Hz, 1H), 1.25 (t, *J* = 7.1 Hz, 3H); ^{13}C NMR (100 MHz, $CDCl_3$) δ 170.7, 164.2, 157.0, 149.6, 146.1, 144.9, 136.7, 130.2, 128.9, 126.1, 118.6, 116.3, 112.2, 62.3, 61.5, 30.1, 14.2; UPCC analysis: 94% ee

(Chiralcel OX-3, CO_2/CH_3OH = 85/15, 1.0 mL/min, detector: 290 nm), Rt (minor) = 9.6 min, Rt (major) = 49.7 min.

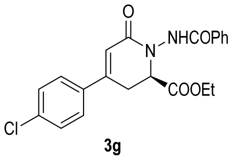

3g

Ethyl (*R*)-1-benzamido-4-(4-chlorophenyl)-6-oxo-1,2,3,6-tetrahydropyridine-2-carboxylate (3g)[5]: white solid; ^{1}H NMR (400 MHz, $CDCl_3$) δ 8.73 (s, 1H), 7.86 (d, *J* = 7.5 Hz, 2H), 7.55 (t, *J* = 7.4 Hz, 1H), 7.51 – 7.37 (m, 6H), 6.33 (d, *J* = 2.5 Hz, 1H), 4.79 (d, *J* = 5.9 Hz, 1H), 4.29 – 4.16 (m, 2H), 3.58 (ddd, *J* = 17.7, 7.2, 2.6 Hz, 1H), 3.33 (dd, *J* = 17.5, 2.3 Hz, 1H), 1.24 (t, *J* = 7.1 Hz, 3H); ^{13}C NMR (100 MHz, $CDCl_3$) δ 170.7, 166.3, 164.0, 148.2, 136.4, 135.1, 132.5, 132.0, 129.2, 128.7, 127.5, 127.3, 118.9, 62.3, 61.1, 30.1, 14.2.; UPCC analysis: 97% ee (Chiralcel OX-3, CO_2/CH_3OH = 80/20, 1.0 mL/min, detector: 290 nm), Rt (minor) = 10.3 min, Rt (major) = 47.2 min.

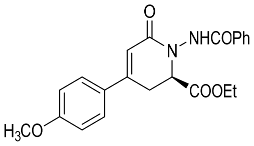

3h

Ethyl (*R*)-1-benzamido-4-(4-methoxyphenyl)-6-oxo-1,2,3,6-tetrahydropyridine-2-carboxylate (3h)[4]: white solid; ^{1}H NMR (400 MHz, $CDCl_3$) δ 9.05 (s, 1H), 7.84 (d, *J* = 7.7 Hz, 2H), 7.56 – 7.38 (m, 5H), 6.93 (d, *J* = 8.7 Hz, 2H), 6.27 (d, *J* = 2.4 Hz, 1H), 4.77 (d, *J* = 5.3 Hz, 1H), 4.20 (q, *J* = 7.0 Hz, 2H), 3.83 (s, 3H), 3.54 (ddd, *J* = 17.5, 7.0, 2.6 Hz, 1H), 3.36 (dd, *J* = 17.4, 2.5 Hz, 1H), 1.22 (t, *J* = 7.1 Hz, 3H); ^{13}C NMR (100 MHz, $CDCl_3$) δ 170.8, 166.2, 164.6, 161.3, 149.0, 132.3, 132.1, 128.9, 128.6, 127.6, 127.5, 116.5, 114.3, 62.1, 61.1, 55.4, 30.0, 14.2; UPCC analysis: 97% ee (Chiralcel OX-3, CO_2/CH_3OH = 80/20, 1.0 mL/min, detector: 290 nm), Rt (minor) = 10.7 min, Rt (major) = 47.3 min.

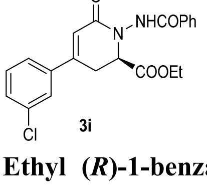

3i

Ethyl (*R*)-1-benzamido-4-(3-chlorophenyl)-6-oxo-1,2,3,6-tetrahydropyridine-2-carboxylate (3i): white solid; ^{1}H NMR (400 MHz, $CDCl_3$) δ 8.97 (s, 1H), 7.85 (d, *J* = 7.6 Hz, 2H), 7.53 (t, *J* = 7.4 Hz, 1H), 7.48 – 7.31 (m, 6H), 6.32 (d, *J* = 2.6 Hz, 1H), 4.78 (dd, *J* = 7.1, 2.3 Hz, 1H), 4.27 – 4.18 (m, 2H), 3.58 (ddd, *J* = 17.5, 7.0, 2.7 Hz, 1H), 3.31 (dd, *J* = 17.5, 2.4 Hz, 1H), 1.25 (t, *J* = 7.1 Hz, 3H); ^{13}C NMR (100 MHz, $CDCl_3$) δ 170.6, 166.2, 163.8, 148.1, 138.6, 135.0, 132.4, 131.8, 130.2, 130.1, 128.7, 127.6, 126.2, 124.2, 119.6, 62.3, 61.0, 30.1, 14.2; IR (film) ν_{max}: 3240, 2981, 1736, 1655, 1425, 1196, 1019, 787, 690; HRMS (ESI) for $C_{21}H_{20}ClN_2O_4^+$ ($[M+H]^+$): calcd 399.1106, found 399.1103; UPCC analysis: 97% ee (Chiralcel OX-3, CO_2/CH_3OH = 80/20, 1.0 mL/min, detector: 290 nm), Rt (minor) = 6.4 min, Rt (major) = 18.2 min.

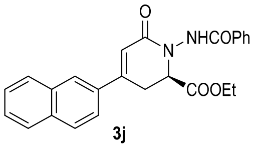

3j

Ethyl (*R*)-1-benzamido-4-(naphthalen-2-yl)-6-oxo-1,2,3,6-tetrahydropyridine-2-carboxylate (3j)[4]: white solid; ^{1}H NMR (400 MHz, $CDCl_3$) δ 8.85 (s, 1H), 7.97 (s, 1H), 7.93 – 7.82 (m, 5H), 7.63 (d, *J* = 8.6 Hz, 1H), 7.59 – 7.51 (m, 3H), 7.46 (t, *J* = 7.5 Hz, 2H), 6.50 (d, *J* = 2.5 Hz, 1H), 4.86 (d, *J* = 6.0 Hz, 1H), 4.23 (q, *J* = 7.1 Hz, 2H), 3.71 (dd, *J* = 17.4, 5.9 Hz, 1H), 3.54 (d, *J* = 16.8 Hz, 1H), 1.25 (t, *J* = 7.1 Hz, 3H); ^{13}C NMR (100 MHz, $CDCl_3$) δ 170.8, 166.3, 164.3, 149.2, 134.0, 133.8, 133.1, 132.4, 132.1, 128.8, 128.7, 128.7, 127.7, 127.5, 127.4, 126.9, 126.1, 123.1, 118.8, 62.2, 61.2, 30.1, 14.2; UPCC analysis: 97% ee (Chiralcel OX-3, CO_2/CH_3OH = 80/20, 1.0 mL/min, detector: 290 nm), Rt (minor) = 15.0 min, Rt (major) = 44.9 min.

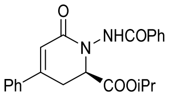

3k

Isopropyl (*R*)-1-benzamido-6-oxo-4-phenyl-1,2,3,6-tetrahydropyridine-2-carboxylate (3k)[6]: white solid; ^{1}H NMR (400 MHz, $CDCl_3$) δ 8.70 (s, 1H), 7.86 (d, *J* = 7.5 Hz, 2H), 7.56 (t, *J* = 7.4 Hz, 1H), 7.52 – 7.40 (m, 7H), 6.34 (s, 1H), 5.07 (hept, *J* = 6.3 Hz, 1H), 4.76 (d, *J* = 6.8 Hz, 1H), 3.58 (dd, *J* = 17.8, 6.9 Hz, 1H), 3.38 (d, *J* = 17.5 Hz, 1H), 1.24 (d, *J* = 6.4 Hz, 3H), 1.20 (d, *J* = 6.1 Hz, 3H); ^{13}C NMR (100 MHz, $CDCl_3$) δ 170.3, 166.2, 164.4, 149.7, 136.8, 132.4, 132.1, 130.2, 129.0, 128.7, 127.5, 126.1, 118.6, 70.2, 61.3, 30.2, 21.8, 21.7; UPCC analysis: 96% ee (Chiralcel OX-3, CO_2/CH_3OH = 85/15, 1.0 mL/min, detector: 290 nm), Rt (minor) = 8.6 min, Rt (major) = 32.5 min.

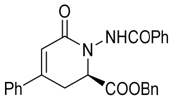

3l

Benzyl (*R*)-1-benzamido-6-oxo-4-phenyl-1,2,3,6-tetrahydropyridine-2-carboxylate (3l)[6]: white solid; ^{1}H NMR (400 MHz, $CDCl_3$) δ 8.75 (s, 1H), 7.80 (d, *J* = 7.6 Hz, 2H), 7.55 (t, *J* = 7.6 Hz, 1H), 7.48 – 7.37 (m, 7H), 7.33 – 7.24 (m, 5H), 6.33 (s, 1H), 5.30 (d, *J* = 12.1 Hz, 1H), 5.11 (d, *J* = 12.1 Hz, 1H), 4.85 (d, *J* = 6.2 Hz, 1H), 3.60 (dd, *J* = 17.6, 6.2 Hz, 1H), 3.37 (d, *J* = 17.4 Hz, 1H); ^{13}C NMR (100 MHz, $CDCl_3$) δ 170.7, 166.2, 164.3, 149.6, 136.6, 134.9, 132.4, 131.9, 130.2, 128.9, 128.7, 128.7, 128.7, 128.3, 127.5, 126.1, 118.5, 67.8, 61.2, 30.1; UPCC analysis: 98% ee (Chiralcel OX-3, CO_2/CH_3OH = 80/20, 1.0 mL/min, detector: 290 nm), Rt (minor) = 11.2 min, Rt (major) = 36.3 min.

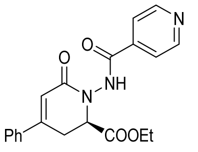

3m

Ethyl (R)-1-(isonicotinamido)-6-oxo-4-phenyl-1,2,3,6-tetrahydropyridine-2-carboxylate (3m): white solid; ^{1}H NMR (400 MHz, $CDCl_3$) δ 9.64 (s, 1H), 8.76 – 8.68 (m, 2H), 7.71 – 7.64 (m, 2H), 7.54 – 7.48 (m, 2H), 7.46 – 7.41 (m, 3H), 6.34 (d, *J* = 2.5 Hz, 1H), 4.74 (dd, *J* = 7.1, 2.4 Hz, 1H), 4.22 (q, *J* = 7.1 Hz, 2H), 3.60 (ddd, *J* = 17.6, 7.1, 2.6 Hz, 1H), 3.40 (dd, *J* = 17.6, 2.4 Hz, 1H), 1.24 (t, *J* = 7.1 Hz, 3H); ^{13}C NMR (100 MHz, $CDCl_3$) δ 170.4, 164.3, 164.2, 150.6, 150.0, 138.8, 136.5, 130.3, 129.0, 126.0, 121.1, 118.3, 62.3, 61.0, 30.1, 14.2; IR (film) ν_{max}: 3210, 2972, 1730, 1652, 1411, 1197, 1021, 762, 690; HRMS (ESI) for $C_{20}H_{20}N_3O_4^+$ ($[M+H]^+$): calcd 366.1448, found 366.1444; UPCC analysis: 92% ee (Chiralcel OX-3, CO_2/CH_3OH, 1.0 mL/min = 85/15, detector: 290 nm), Rt (minor) = 14.7 min, Rt (major) = 16.4 min.

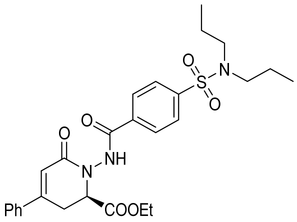

3n

Ehyl (R)-1-(4-(N,N-dipropylsulfamoyl)benzamido)-6-oxo-4-phenyl-1,2,3,6-tetrahydropyridine-2-carboxylate (3n): white solid; ^{1}H NMR (400 MHz, $CDCl_3$) δ 9.60 (s, 1H), 8.00 – 7.93 (m, 2H), 7.84 – 7.78 (m, 2H), 7.52 – 7.45 (m, 2H), 7.45 – 7.38 (m, 3H), 6.31 (d, *J* = 2.5 Hz, 1H), 4.74 (dd, *J* = 7.1, 2.4 Hz, 1H), 4.20 (q, *J* = 7.1 Hz, 2H), 3.58 (ddd, *J* = 17.6, 7.1, 2.6 Hz, 1H), 3.38 (dd, *J* = 17.6, 2.5 Hz, 1H), 3.11 – 2.96 (m, 4H), 1.52 (h, *J* = 7.4 Hz, 4H), 1.22 (t, *J* = 7.2 Hz, 3H), 0.85 (t, *J* = 7.4 Hz, 6H); ^{13}C NMR (100 MHz, $CDCl_3$) δ 170.5, 164.7, 164.3, 149.9, 143.5, 136.4, 135.0, 130.2, 128.9, 128.3, 127.1, 126.0, 118.2, 62.2, 61.0, 49.9, 30.1, 21.9, 14.1, 11.1; IR (film) ν_{max}: 3205, 2965, 1734, 1683, 1447, 1234, 1055, 868, 693; HRMS (ESI) for $C_{27}H_{34}N_3O_6S^+$ ($[M+H]^+$): calcd 528.2163, found 328.2155; UPCC analysis: 94% ee (Chiralcel OX-3, CO_2/CH_3OH, 1.0 mL/min = 80/20, detector: 290 nm), Rt (minor) = 14.4 min, Rt (major) = 19.5min.

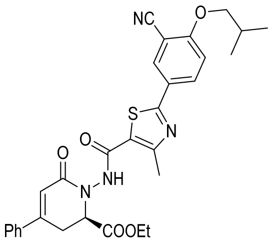

3o

Ethyl (R)-1-(2-(3-cyano-4-isobutoxyphenyl)-4-methylthiazole-5-carboxamido)-6-oxo-4-phenyl-1,2,3,6-tetrahydropyridine-2-carboxylate (3o): white solid; ^{1}H NMR (400 MHz, $CDCl_3$) δ 8.57 (s, 1H), 8.15 (d, *J* = 2.2 Hz, 1H), 8.06 (dd, *J* = 8.9, 2.3 Hz, 1H), 7.53 – 7.46 (m, 2H), 7.45 – 7.39 (m, 3H), 7.01 (d, *J* = 8.9 Hz, 1H), 6.34 (d, *J* = 2.4 Hz, 1H), 4.73 (dd, *J* = 7.1, 2.3 Hz, 1H), 4.24 (qq, *J* = 6.8, 3.7 Hz, 2H), 3.89 (d, *J* = 6.5 Hz, 2H), 3.57 (ddd, *J* = 17.6, 7.1, 2.6 Hz, 1H), 3.39 (dd, *J* = 17.6, 2.3 Hz, 1H), 2.76 (s, 3H), 2.20 (hept, *J* = 6.6 Hz, 1H), 1.26 (t, *J* = 7.1 Hz, 3H), 1.08 (d, *J* = 6.7 Hz, 6H); ^{13}C NMR (100 MHz, $CDCl_3$) δ 170.6, 165.8, 164.3, 162.5, 161.0, 158.8, 149.9, 136.5, 132.6, 132.0, 130.3, 128.9, 126.0, 125.7, 118.2, 115.3, 112.6, 102.9, 75.7, 62.4, 61.3, 30.0, 28.1, 19.0, 17.7, 14.2; IR (film) ν_{max}: 3214, 2976, 1732, 1636, 1508, 1427, 1221, 1011, 775, 694; HRMS (ESI) for $C_{30}H_{31}N_4O_5S^+$ ($[M+H]^+$): calcd 559.2010, found 559.1998; UPCC analysis: 94% ee (Chiralcel OX-3, CO_2/CH_3OH, 1.0 mL/min = 60/40, detector: 290 nm), Rt (minor) = 9.6 min, Rt (major) = 12.7 min.

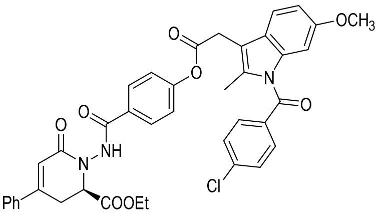

3p

Ethyl (R)-1-(4-(2-(1-(4-chlorobenzoyl)-6-methoxy-2-methyl-1H-indol-3-yl)acetoxy)benzamido)-6-oxo-4-phenyl-1,2,3,6-tetrahydropyridine-2-carboxylate (3p): white solid; ^{1}H NMR (400 MHz, $CDCl_3$) δ 9.49 (s, 1H), 7.87 – 7.80 (m, 2H), 7.71 – 7.65 (m, 2H), 7.53 – 7.45 (m, 4H), 7.45 – 7.38 (m, 3H), 7.13 – 7.08 (m, 2H), 7.04 (d, *J* = 2.5 Hz, 1H), 6.89 (d, *J* = 9.0 Hz, 1H), 6.70 (dd, *J* = 9.1, 2.5 Hz, 1H), 6.33 (d, *J* = 2.5 Hz, 1H), 4.73 (dd, *J* = 7.2, 2.3 Hz, 1H), 4.22 (q, *J* = 7.1 Hz, 2H), 3.92 (s, 2H), 3.83 (s, 3H), 3.58 (ddd, *J* = 17.6, 7.2, 2.7 Hz, 1H), 3.38 (dd, *J* = 17.6, 2.4 Hz, 1H), 2.46 (s, 3H), 1.24 (t, *J* = 7.1 Hz, 3H); ^{13}C NMR (100 MHz, $CDCl_3$) δ 170.6, 168.6, 168.2, 165.1, 164.2, 156.1, 153.6, 149.7, 139.3, 136.6, 136.3, 133.7, 131.2, 130.8, 130.4, 130.1, 129.2, 129.1, 129.0, 128.9, 126.0, 121.6, 118.4, 115.0, 111.8, 111.6, 101.1, 62.2, 61.0, 55.7, 30.5, 30.1, 14.2, 13.4; IR (film) ν_{max}: 3292, 2981, 1736, 1647, 1425, 1197, 1023, 763, 655; HRMS (ESI) for $C_{40}H_{34}ClN_3O_8Na^+$ ($[M+Na]^+$): calcd 742.1927, found 742.1907; UPCC analysis: 97% ee (Chiralcel OX-3, CO_2/CH_3OH = 60/40, 1.0 mL/min, detector: 290 nm), Rt (minor) = 10.8 min, Rt (major) = 23.7 min.

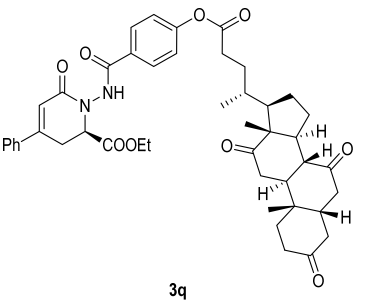

3q

Ehyl (R)-1-(4-(((R)-4-((5S,8R,9S,10S,13R,14S,17R)-10,13-dimethyl-3,7,12-trioxohexadecahydro-1H-cyclopenta[a]phenanthren-17-yl)pentanoyl)oxy)benzamido)-6-oxo-4-phenyl-1,2,3,6-tetrahy-

dropyridine-2-carboxylate (3q): white solid; ^{1}H NMR (400 MHz, $CDCl_3$) δ 9.09 (s, 1H), 7.88 (d, *J* = 8.7 Hz, 2H), 7.55 – 7.47 (m, 2H), 7.46 – 7.36 (m, 3H), 7.15 (d, *J* = 8.7 Hz, 2H), 6.33 (d, *J* = 2.6 Hz, 1H), 4.75 (dd, *J* = 7.1, 2.3 Hz, 1H), 4.21 (q, *J* = 7.1 Hz, 2H), 3.59 (ddd, *J* = 17.5, 7.1, 2.6 Hz, 1H), 3.37 (dd, *J* = 17.5, 2.4 Hz, 1H), 2.98 – 2.78 (m, 3H), 2.72 – 2.61 (m, 1H), 2.53 (ddd, *J* = 16.1, 8.9, 7.1 Hz, 1H), 2.40 – 2.27 (m, 3H), 2.27 – 2.11 (m, 4H), 2.10 – 1.92 (m, 5H), 1.86 (td, *J* = 11.4, 7.1 Hz, 1H), 1.66 – 1.47 (m, 2H), 1.46 – 1.15 (m, 10H), 1.09 (s, 3H), 0.91 (d, *J* = 6.6 Hz, 3H); ^{13}C NMR (100 MHz, $CDCl_3$) δ 211.9, 209.1, 208.7, 171.9, 170.7, 165.3, 164.2, 153.8, 149.6, 136.6, 130.1, 129.2, 129.0, 128.9, 126.0, 121.8, 118.4, 62.2, 61.1, 56.9, 51.7, 48.9, 46.8, 45.6, 45.5, 44.9, 42.7, 38.6, 36.4, 36.0, 35.4, 35.2, 31.5, 30.2, 30.1, 27.6, 25.1, 21.9, 18.6, 14.2, 11.8; IR (film) ν_{max}: 3253, 2952, 1709, 1655, 1338, 1196, 913, 732; HRMS (ESI) for $C_{45}H_{52}N_2O_9Na^+$ ($[M+Na]^+$): calcd 787.3565, found 787.3540; UPCC analysis: 98% de (Chiralcel OX-3, CO_2/CH_3OH = 50/50, 1.0 mL/min, detector: 290 nm), Rt (minor) = 26.0 min, Rt (major) = 48.8 min.

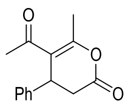

6a

5-acetyl-6-methyl-4-phenyl-3,4-dihydro-2H-pyran-2-one (6a)[7]: white solid; ^{1}H NMR (400 MHz, $CDCl_3$) δ 7.37 – 7.27 (m, 3H), 7.17 – 7.11 (m, 2H), 4.14 (d, *J* = 6.3 Hz, 1H), 2.97 (dd, *J* = 15.7, 7.2 Hz, 1H), 2.83 (dd, *J* = 15.6, 2.6 Hz, 1H), 2.43 (s, 3H), 2.12 (s, 3H); ^{13}C NMR (100 MHz, $CDCl_3$) δ 197.9, 165.6, 160.3, 139.7, 129.5, 128.0, 126.7, 117.3, 38.9, 37.2, 29.8, 19.1.

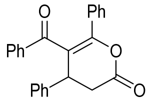

6b

5-benzoyl-4,6-diphenyl-3,4-dihydro-2H-pyran-2-one (6b)[7]**:** white solid; ^{1}H NMR (400 MHz, $CDCl_3$) δ 7.51 – 7.46 (m, 2H), 7.39 – 7.34 (m, 2H), 7.33 – 7.27 (m, 4H), 7.25 – 7.20 (m, 2H), 7.19 – 7.05 (m, 5H), 4.56 (dd, *J* = 7.9, 2.4 Hz, 1H), 3.21 (dd, *J* = 15.9, 7.8 Hz, 1H), 3.06 (dd, *J* = 15.9, 2.5 Hz, 1H).; ^{13}C NMR (100 MHz, $CDCl_3$) δ 195.9, 166.7, 154.9, 139.7, 137.1, 132.6, 132.0, 130.3, 129.3, 129.0, 128.1, 128.0, 127.8, 126.9, 118.3, 40.4, 36.0.

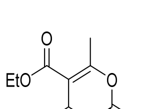

6c

Ethyl 6-methyl-2-oxo-4-phenyl-3,4-dihydro-2H-pyran-5-carboxylate (6c)[7]**:** white solid; ^{1}H NMR (400 MHz, $CDCl_3$) δ 7.33 – 7.27 (m, 2H), 7.25 – 7.20 (m, 1H), 7.16 – 7.11 (m, 2H), 4.25 (d, *J* = 7.2 Hz, 1H), 4.13 (q, *J* = 7.1 Hz, 2H), 2.95 (dd, *J* = 15.9, 7.6 Hz, 1H), 2.82 (dd, *J* = 15.8, 2.4 Hz, 1H), 2.47 (s, 3H), 1.19 (t, *J* = 7.1 Hz, 3H); ^{13}C NMR (100 MHz, $CDCl_3$) δ 166.2, 166.0, 161.3, 140.6, 129.0,

127.5, 126.6, 110.0, 60.9, 37.8, 36.4, 18.9, 14.1.

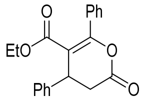

6d

Ethyl 2-oxo-4,6-diphenyl-3,4-dihydro-2H-pyran-5-carboxylate (6d)[7]: white solid; ^{1}H NMR (400 MHz, $CDCl_3$) δ 7.54 – 7.49 (m, 2H), 7.48 – 7.38 (m, 3H), 7.37 – 7.31 (m, 2H), 7.30 – 7.26 (m, 2H), 7.26 – 7.20 (m, 1H), 4.41 (dd, *J* = 7.7, 2.5 Hz, 1H), 3.98 – 3.89 (m, 2H), 3.11 (dd, *J* = 15.9, 7.7 Hz, 1H), 2.95 (dd, *J* = 15.9, 2.6 Hz, 1H), 0.88 (t, *J* = 7.1 Hz, 3H); ^{13}C NMR (100 MHz, $CDCl_3$) δ 166.4, 166.0, 158.5, 139.9, 133.1, 130.1, 129.2, 128.6, 128.0, 127.8, 126.8, 111.7, 61.0, 38.9, 36.3, 13.5.

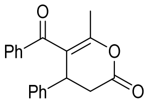

6e

5-benzoyl-6-methyl-4-phenyl-3,4-dihydro-2H-pyran-2-one (6e)[7]: yellow solid; ^{1}H NMR (400 MHz, $CDCl_3$) δ 7.67 – 7.61 (m, 2H), 7.56 – 7.50 (m, 1H), 7.41 (t, *J* = 7.6 Hz, 2H), 7.31 – 7.26 (m, 2H), 7.25 – 7.19 (m, 1H), 7.18 – 7.11 (m, 2H), 4.33 (dd, *J* = 7.9, 3.5 Hz, 1H), 3.08 (dd, *J* = 16.0, 7.5 Hz, 1H), 2.95 (dd, *J* = 16.0, 3.6 Hz, 1H), 1.91 (s, 3H); ^{13}C NMR (100 MHz, $CDCl_3$) δ 195.8, 166.5, 154.8, 140.0, 138.4, 133.1, 129.2, 128.8, 128.7, 127.6, 126.8, 117.8, 39.4, 36.2, 19.0.

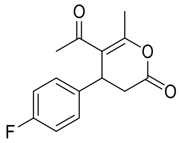

6f

5-acetyl-4-(4-fluorophenyl)-6-methyl-3,4-dihydro-2H-pyran-2-one (6f)[8]: yellow solid; ^{1}H NMR (400 MHz, $CDCl_3$) δ 7.15 – 7.08 (m, 2H), 7.05 – 6.98 (m, 2H), 4.15 (d, *J* = 6.1 Hz, 1H), 2.95 (dd, *J* = 15.7, 7.2 Hz, 1H), 2.80 (dd, *J* = 15.6, 2.6 Hz, 1H), 2.42 (d, *J* = 1.1 Hz, 3H), 2.13 (s, 3H); ^{13}C NMR (100 MHz, $CDCl_3$) δ 197.6, 165.4, 162.3 (*J* = 245 Hz), 160.4, 135.5 (*J* = 3 Hz), 128.4 (*J* = 8 Hz), 117.4, 116.4 (*J* = 22 Hz), 38.1, 37.2, 29.8, 19.2.

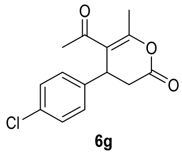

6g

5-acetyl-4-(4-chlorophenyl)-6-methyl-3,4-dihydro-2H-pyran-2-one (6g)[8]: white solid; ^{1}H NMR (400 MHz, $CDCl_3$) δ 7.31 (d, *J* = 8.1 Hz, 2H), 7.08 (d, *J* = 8.1 Hz, 2H), 4.15 (d, *J* = 7.2 Hz, 1H), 2.96 (dd, *J* = 15.7, 7.3 Hz, 1H), 2.80 (dd, *J* = 15.7, 2.5 Hz, 1H), 2.43 (s, 3H), 2.14 (s, 3H); ^{13}C NMR (100 MHz, $CDCl_3$) δ 197.4, 165.3, 160.6, 138.3, 133.9, 129.6, 128.1, 117.2, 38.2, 37.0, 29.9, 19.2.

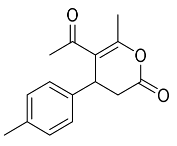

6h

5-acetyl-6-methyl-4-(p-tolyl)-3,4-dihydro-2H-pyran-2-one (6h)[9]: white solid; ^{1}H NMR (400 MHz, $CDCl_3$) δ 7.13 (d, *J* = 7.8 Hz, 2H), 7.02 (d, *J* = 7.9 Hz, 2H), 4.10 (dd, *J* = 7.3, 2.5 Hz, 1H), 2.95 (dd, *J* = 15.6, 7.1 Hz, 1H), 2.81 (dd, *J* = 15.6, 2.7 Hz, 1H), 2.42 (s, 3H), 2.31 (s, 3H), 2.11 (s, 3H); ^{13}C NMR (100 MHz, $CDCl_3$) δ 198.1, 165.7, 160.1, 137.7, 136.6, 130.1, 126.5, 117.4, 38.5, 37.3, 29.7, 21.0, 19.1.

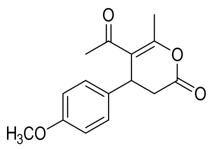

6i

5-acetyl-4-(4-methoxyphenyl)-6-methyl-3,4-dihydro-2H-pyran-2-one (6i)[7]: yellow solid; ^{1}H NMR (400 MHz, $CDCl_3$) δ 7.05 (d, *J* = 8.6 Hz, 2H), 6.85 (d, *J* = 8.6 Hz, 2H), 4.09 (d, *J* = 6.3 Hz, 1H), 3.78 (s, 3H), 2.93 (dd, *J* = 15.6, 7.1 Hz, 1H), 2.80 (dd, *J* = 15.6, 2.6 Hz, 1H), 2.41 (s, 3H), 2.12 (s, 3H); ^{3}C NMR (100 MHz, $CDCl_3$) δ 198.1, 165.8, 160.0, 159.2, 131.5, 127.8, 117.6, 114.8, 55.3, 38.1, 37.4, 29.7, 19.1.

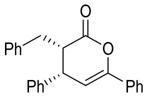

9a

(3*S*,4*S*)-3-benzyl-4,6-diphenyl-3,4-dihydro-2H-pyran-2-one (9a)[10]: white solid; ^{1}H NMR (400 MHz, $CDCl_3$) δ 7.69 – 7.62 (m, 2H), 7.42 – 7.28 (m, 8H), 7.26 – 7.20 (m, 1H), 7.17 – 7.06 (m, 4H), 6.03 (d, *J* = 6.7 Hz, 1H), 3.67 (t, *J* = 6.8 Hz, 1H), 3.40 (ddd, *J* = 9.2, 6.9, 5.2 Hz, 1H), 3.29 (dd, *J* = 14.6, 5.1 Hz, 1H), 2.45 (dd, *J* = 14.7, 9.2 Hz, 1H); ^{13}C NMR (100 MHz, $CDCl_3$) δ 170.2, 150.0, 138.7, 137.8, 132.2, 129.2, 129.0, 129.0, 128.6, 128.5, 128.5, 127.8, 126.5, 124.7, 105.2, 45.7, 40.8, 32.3; UPCC analysis: major diastereomer 99% ee (Chiralcel OD-3, CO_2/CH_3OH = 95/5, 1.0 mL/min, detector: 254 nm), Rt (major) = 12.8 min, Rt (minor) = 14.3 min.

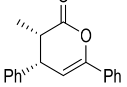

9b

(3*S*,4*S*)-3-methyl-4,6-diphenyl-3,4-dihydro-2H-pyran-2-one (9b)[10]: white solid; ^{1}H NMR (400 MHz, $CDCl_3$) δ 7.71 (dd, *J* = 7.7, 1.9 Hz, 2H), 7.45 – 7.27 (m, 6H), 7.20 – 7.13 (m, 2H), 6.08 (d, *J* =

6.3 Hz, 1H), 3.75 (t, J = 6.8 Hz, 1H), 3.16 (p, J = 7.0 Hz, 1H), 1.09 (d, J = 6.9 Hz, 3H); ^{13}C NMR (100 MHz, $CDCl_3$) δ 171.0, 150.4, 137.7, 132.3, 129.2, 128.9, 128.6, 128.3, 127.7, 124.7, 104.6, 43.3, 39.0, 12.5; UPCC analysis: major diastereomer 98% ee (Chiralcel OD-3, CO_2/CH_3OH = 97/3, 1.0 mL/min, detector: 254 nm), Rt (major) = 10.0 min, Rt (minor) = 10.7 min.

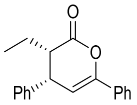

9c

(3*S*,4*S*)-3-ethyl-4,6-diphenyl-3,4-dihydro-2H-pyran-2-one (9c)[10]: white solid; ^{1}H NMR (400 MHz, $CDCl_3$) δ 7.71 – 7.65 (m, 2H), 7.42 – 7.35 (m, 3H), 7.35 – 7.26 (m, 3H), 7.20 – 7.15 (m, 2H), 6.07 (d, J = 6.3 Hz, 1H), 3.85 (t, J = 6.7 Hz, 1H), 2.86 (q, J = 7.0 Hz, 1H), 1.84 – 1.71 (m, 1H), 1.31 – 1.19 (m, 1H), 1.03 (t, J = 7.4 Hz, 3H); ^{13}C NMR (100 MHz, $CDCl_3$) δ 170.4, 150.2, 138.0, 132.3, 129.1, 128.9, 128.5, 128.1, 127.6, 124.7, 104.8, 46.0, 41.3, 19.8, 12.1; UPCC analysis: major diastereomer 98% ee (Chiralcel OD-3, CO_2/CH_3OH = 99.3/0.7, 1.0 mL/min, detector: 254 nm), Rt (major) = 24.6 min, Rt (minor) = 28.9 min.

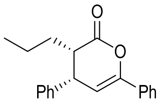

9d

(3*S*,4*S*)-4,6-diphenyl-3-propyl-3,4-dihydro-2H-pyran-2-one (9d)[11]: white solid; ^{1}H NMR (400 MHz, DCl_3) δ 7.68 (dd, J = 7.7, 1.9 Hz, 2H), 7.43 – 7.35 (m, 3H), 7.35 – 7.26 (m, 3H), 7.20 – 7.14 (m, 2H), 6.07 (d, J = 6.4 Hz, 1H), 3.82 (t, J = 6.7 Hz, 1H), 2.95 (q, J = 6.9 Hz, 1H), 1.76 – 1.64 (m, 1H), 1.53 – 1.38 (m, 2H), 1.27 – 1.12 (m, 1H), 0.89 (t, J = 7.3 Hz, 3H); ^{13}C NMR (100 MHz, $CDCl_3$) δ 170.6, 150.1, 138.1, 132.3, 129.1, 128.9, 128.5, 128.1, 127.6, 124.7, 104.8, 44.0, 41.6, 28.7, 20.6, 13.9; UPCC analysis: major diastereomer 99% ee (Chiralcel OD-3, CO_2/CH_3OH = 97/3, 1.0 mL/min, detector: 254 nm), Rt (major) = 7.9 min, Rt (minor) = 9.5 min.

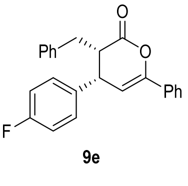

9e

(3*S*,4*S*)-3-benzyl-4-(4-fluorophenyl)-6-phenyl-3,4-dihydro-2H-pyran-2-one (9e): white solid; ^{1}H NMR (400 MHz, $CDCl_3$) δ 7.69 – 7.64 (m, 2H), 7.43 – 7.36 (m, 3H), 7.35 – 7.29 (m, 2H), 7.29 – 7.23 (m, 2H), 7.16 – 7.10 (m, 2H), 7.08 – 6.97 (m, 4H), 6.02 (d, J = 6.7 Hz, 1H), 3.66 (t, J = 6.8 Hz, 1H), 3.41 (ddd, J = 9.4, 6.9, 5.0 Hz, 1H), 3.30 (dd, J = 14.7, 5.1 Hz, 1H), 2.41 (dd, J = 14.7, 9.4 Hz, 1H); ^{13}C NMR (100 MHz, $CDCl_3$) δ 170.0, 162.3 (J = 246 Hz), 161.1, 150.2, 138.4, 133.5 (J = 3 Hz), 132.0, 130.1 (J = 8 Hz), 129.3, 128.9, 128.6, 128.6, 126.6, 124.7, 115.8 (J = 21 Hz), 105.0, 45.5, 40.0, 32.3; IR (film) ν_{max}: 3063, 2925, 1769, 1604, 1509, 1227, 1131, 1078, 836, 763, 540; HRMS (ESI) for

$C_{24}H_{19}FNaO_2^+$ ([M+Na]$^+$): calcd 381.1261, found 381.1254; UPCC analysis: major diastereomer 99% ee (Chiralcel OD-3, CO_2/CH_3OH = 95/5, 1.0 mL/min, detector: 254 nm), Rt (major) = 10.0 min, Rt (minor) = 12.8 min.

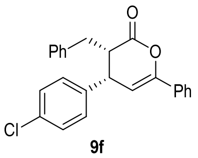

9f

(3*S*,4*S*)-3-benzyl-4-(4-chlorophenyl)-6-phenyl-3,4-dihydro-2H-pyran-2-one (9f)[10]: white solid; ^1H NMR (400 MHz, $CDCl_3$) δ 7.58 – 7.51 (m, 2H), 7.33 – 7.26 (m, 3H), 7.24 – 7.12 (m, 5H), 7.05 – 6.98 (m, 2H), 6.94 – 6.87 (m, 2H), 5.89 (d, *J* = 6.7 Hz, 1H), 3.53 (t, *J* = 6.8 Hz, 1H), 3.30 (ddd, *J* = 9.4, 6.9, 5.1 Hz, 1H), 3.20 (dd, *J* = 14.7, 5.1 Hz, 1H), 2.30 (dd, *J* = 14.7, 9.5 Hz, 1H); ^{13}C NMR (100 MHz, $CDCl_3$) δ 169.9, 150.3, 138.3, 136.3, 133.7, 131.9, 129.8, 129.4, 129.1, 128.9, 128.7, 128.6, 126.7, 124.7, 104.7, 45.3, 40.1, 32.3; UPCC analysis: major diastereomer 96% ee (Chiralcel OD-3, CO_2/CH_3OH = 95/5, 1.0 mL/min, detector: 254 nm), Rt (major) = 15.8 min, Rt (minor) = 21.4 min.

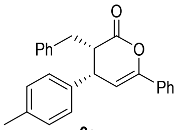

9g

(3*S*,4*S*)-3-benzyl-6-phenyl-4-(p-tolyl)-3,4-dihydro-2H-pyran-2-one (9g): white solid; ^1H NMR (400 MHz, $CDCl_3$) δ 7.69 – 7.62 (m, 2H), 7.43 – 7.28 (m, 5H), 7.26 – 7.21 (m, 1H), 7.14 (d, *J* = 8.1 Hz, 4H), 6.99 (d, *J* = 7.6 Hz, 2H), 6.02 (d, *J* = 6.6 Hz, 1H), 3.63 (t, *J* = 6.5 Hz, 1H), 3.41 – 3.33 (m, 1H), 3.28 (dd, *J* = 14.7, 5.0 Hz, 1H), 2.45 (dd, *J* = 14.3, 9.4 Hz, 1H), 2.35 (s, 3H); ^{13}C NMR (100 MHz, $CDCl_3$) δ 170.3, 149.9, 138.8, 137.6, 134.7, 132.2, 129.6, 129.1, 129.0, 128.5, 128.3, 126.5, 124.6, 105.4, 45.8, 40.4, 32.3, 21.1; IR (film) ν_{max}: 3061, 2924, 1768, 1662, 1496, 1130, 1077, 762, 692, 538; HRMS (ESI) for $C_{25}H_{22}NaO_2^+$ ([M+Na]$^+$): calcd 377.1512, found 377.1509; UPCC analysis: major diastereomer 99% ee (Chiralcel OD-3, CO_2/CH_3OH = 95/5, 1.0 mL/min, detector: 254 nm), Rt (major) = 12.4 min, Rt (minor) = 14.2 min.

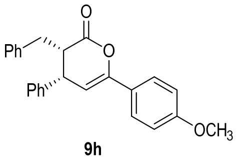

9h

(3*S*,4*S*)-3-benzyl-6-(4-methoxyphenyl)-4-phenyl-3,4-dihydro-2H-pyran-2-one (9h): white solid; ^1H NMR (400 MHz, $CDCl_3$) δ 7.59 (d, *J* = 8.4 Hz, 2H), 7.37 – 7.21 (m, 6H), 7.12 (dd, *J* = 14.0, 7.4 Hz, 4H), 6.91 (d, *J* = 8.4 Hz, 2H), 5.89 (d, *J* = 6.6 Hz, 1H), 3.83 (s, 3H), 3.64 (t, *J* = 6.8 Hz, 1H), 3.43 – 3.34 (m, 1H), 3.28 (dd, *J* = 14.7, 5.1 Hz, 1H), 2.44 (dd, *J* = 14.7, 9.2 Hz, 1H); ^{13}C NMR (100 MHz, $CDCl_3$) δ 170.4, 160.4, 149.8, 138.7, 138.1, 129.0, 128.9, 128.5, 128.4, 127.8, 126.5, 126.1, 124.8, 113.9, 103.4, 55.4, 45.7, 40.8, 32.4; IR (film) ν_{max}: 3029, 2924, 1765, 1609, 1513, 1455, 1254, 1178, 1078, 764, 698; HRMS (ESI) for $C_{25}H_{22}NaO_3^+$ ([M+Na]$^+$): calcd 393.1461, found 393.1457; UPCC

analysis: major diastereomer 99% ee (Chiralcel OD-3, CO_2/CH_3OH = 95/5, 1.0 mL/min, detector: 254 nm), Rt (major) = 22.1 min, Rt (minor) = 29.7 min.

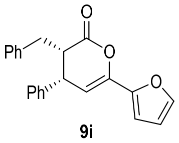

9i

(3*S*,4*S*)-3-benzyl-6-(furan-2-yl)-4-phenyl-3,4-dihydro-2H-pyran-2-one (9i)[10]**:** white solid; ^{1}H NMR (400 MHz, $CDCl_3$) δ 7.39 (d, *J* = 1.7 Hz, 1H), 7.37 – 7.18 (m, 8H), 7.14 – 7.07 (m, 4H), 6.66 (d, *J* = 3.4 Hz, 1H), 6.46 (dd, *J* = 3.4, 1.8 Hz, 1H), 5.98 (d, *J* = 6.7 Hz, 1H), 3.67 (t, *J* = 6.8 Hz, 1H), 3.39 (ddd, *J* = 9.2, 6.9, 5.1 Hz, 1H), 3.27 (dd, *J* = 14.7, 5.1 Hz, 1H), 2.43 (dd, *J* = 14.7, 9.2 Hz, 1H); ^{13}C NMR (100 MHz, $CDCl_3$) δ 169.7, 146.8, 143.2, 142.9, 138.5, 137.8, 129.0, 128.5, 128.4, 127.8, 126.6, 111.5, 107.9, 103.6, 45.9, 40.5, 32.4; UPCC analysis: major diastereomer 99% ee (Chiralcel OD-3, CO_2/CH_3OH = 97/3, 1.0 mL/min, detector: 235 nm), Rt (major) = 12.2 min, Rt (minor) = 17.0 min.

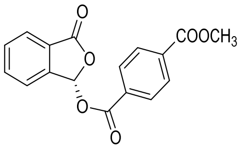

12a

(*S*)-methyl (3-oxo-1,3-dihydroisobenzofuran-1-yl) terephthalate (12a)[12]**:** white solid; ^{1}H NMR (400 MHz, $CDCl_3$) δ 8.10 (s, 4H), 7.98 (d, *J* = 7.8 Hz, 1H), 7.78 (t, *J* = 7.5 Hz, 1H), 7.72 – 7.65 (m, 3H), 3.94 (s, 3H); ^{13}C NMR (100 MHz, $CDCl_3$) δ 167.7, 166.0, 164.4, 144.2, 135.0, 134.9, 132.1, 131.5, 130.1, 129.7, 126.6, 125.9, 123.8, 93.4, 52.5; UPCC analysis: 95% ee (Chiralpak AD-3, CO_2/CH_3OH = 90/10, 1.0 mL/min, detector: 254 nm), Rt (major) = 6.2 min, Rt (minor) = 12.2 min.

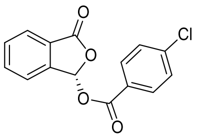

12b

(*S*)-3-oxo-1,3-dihydroisobenzofuran-1-yl 4-chlorobenzoate (12b)[12]**:** white solid; ^{1}H NMR (400 MHz, $CDCl_3$) δ 8.04 – 7.95 (m, 3H), 7.78 (t, *J* = 7.5 Hz, 1H), 7.74 – 7.63 (m, 3H), 7.43 (d, *J* = 8.2 Hz, 2H); ^{13}C NMR (100 MHz, $CDCl_3$) δ 167.8, 164.3, 144.3, 140.7, 134.9, 131.5, 131.4, 129.0, 126.9, 126.6, 125.9, 123.7, 93.3; UPCC analysis: 96% ee (Chiralpak AD-3, CO_2/CH_3OH = 90/10, 1.0 mL/min, detector: 254 nm), Rt (major) = 4.8 min, Rt (minor) = 11.9 min.

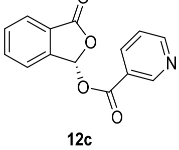

12c

(*R*)-3-oxo-1,3-dihydroisobenzofuran-1-yl nicotinate (12c)[13]: white solid; ^{1}H NMR (400 MHz, $CDCl_3$) δ 9.23 (s, 1H), 8.82 (dd, *J* = 4.9, 1.8 Hz, 1H), 8.35 – 8.27 (m, 1H), 7.98 (d, *J* = 7.5 Hz, 1H), 7.79 (t, *J* = 7.6 Hz, 1H), 7.73 – 7.65 (m, 3H), 7.41 (dd, *J* = 7.9, 5.1 Hz, 1H); ^{13}C NMR (100 MHz, $CDCl_3$) δ 167.6, 163.9, 154.4, 151.3, 144.0, 137.5, 135.0, 131.5, 126.5, 126.0, 124.6, 123.7, 123.5, 93.3; UPCC analysis: 92% ee (Chiralpak AD-3, CO_2/CH_3OH = 85/15, 1.0 mL/min, detector: 254 nm), Rt (major) = 5.5 min, Rt (minor) = 19.0 min.

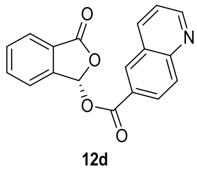

12d

(*S*)-3-oxo-1,3-dihydroisobenzofuran-1-yl quinoline-6-carboxylate (12d): white solid; ^{1}H NMR (400 MHz, $CDCl_3$) δ 9.01 (d, *J* = 4.1 Hz, 1H), 8.60 (s, 1H), 8.26 (dd, *J* = 17.4, 8.6 Hz, 2H), 8.14 (d, *J* = 8.9 Hz, 1H), 7.98 (d, *J* = 7.6 Hz, 1H), 7.83 – 7.65 (m, 4H), 7.47 (dd, *J* = 8.4, 4.2 Hz, 1H); ^{13}C NMR (100 MHz, $CDCl_3$) δ 167.8, 164.6, 153.1, 150.4, 144.3, 137.4, 135.0, 132.1, 131.5, 130.2, 128.9, 127.4, 126.6, 126.3, 125.9, 123.8, 122.1, 93.5; IR (film) ν_{max}: 3055, 1783, 1735, 1624, 1266, 1177, 970, 751, 688; HRMS (ESI) for $C_{18}H_{12}NO_4^+$ ($[M+H]^+$): calcd 306.0761, found 306.0758; UPCC analysis: 95% ee (Chiralpak AD-3, CO_2/CH_3OH = 85/15, 1.0 mL/min, detector: 254 nm), Rt (major) = 8.8 min, Rt (minor) = 18.1 min.

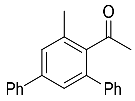

14

1-(5'-methyl-[1,1':3',1''-terphenyl]-4'-yl)ethan-1-one (14)[14]: yellow oil; ^{1}H NMR (400 MHz, $CDCl_3$) δ 7.68 – 7.60 (m, 2H), 7.50 – 7.34 (m, 10H), 2.41 (s, 3H), 1.97 (s, 3H); ^{13}C NMR (100 MHz, $CDCl_3$) δ 207.6, 141.8, 140.5, 140.2, 139.3, 134.5, 129.0, 128.9, 128.7, 128.4, 127.9, 127.8, 127.2, 126.2, 32.2, 19.8.

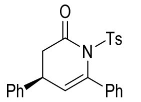

16

(*S*)-4,6-diphenyl-1-tosyl-3,4-dihydropyridin-2(1H)-one (16)[15]: white solid; ^{1}H NMR (400 MHz, $CDCl_3$) δ 7.78 (d, *J* = 7.9 Hz, 2H), 7.45 – 7.27 (m, 9H), 7.25 – 7.14 (m, 3H), 5.99 (d, *J* = 4.3 Hz, 1H), 3.87 (q, *J* = 7.0 Hz, 1H), 2.83 (d, *J* = 8.0 Hz, 2H), 2.44 (s, 3H); ^{13}C NMR (100 MHz, $CDCl_3$) δ 171.7, 145.0, 141.0, 140.5, 137.2, 136.5, 129.2, 129.1, 129.0, 128.4, 128.4, 127.4, 127.1, 126.0, 123.2, 42.9, 37.2, 21.7; UPCC analysis: 97% ee (Chiralcel OX-3, CO_2/CH_3OH = 80/20, 1.0 mL/min, detector: 254 nm), Rt (major) = 6.7min, Rt (minor) = 9.9 min.

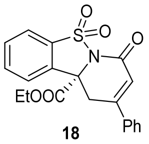

18

Ethyl (*S*)-7-oxo-9-phenyl-7,10-dihydro-10aH-benzo[4,5]isothiazolo[2,3-a]pyridine-10a carbo-xyl-ate 5,5-dioxide (18)[16]: white solid; ^{1}H NMR (400 MHz, $CDCl_3$) δ 7.93 (d, *J* = 7.7 Hz, 1H), 7.81 – 7.76 (m, 2H), 7.74 – 7.67 (m, 1H), 7.58 – 7.53 (m, 2H), 7.50 – 7.44 (m, 3H), 6.40 (d, *J* = 2.6 Hz, 1H), 4.27 – 4.09 (m, 3H), 3.05 (dd, *J* = 16.8, 2.7 Hz, 1H), 1.18 (t, *J* = 7.1 Hz, 3H); ^{13}C NMR (100 MHz, $CDCl_3$) δ 169.0, 160.5, 152.8, 136.2, 134.9, 134.4, 132.4, 131.3, 130.9, 129.2, 126.5, 124.0, 122.2, 119.7, 68.1, 63.8, 36.9, 13.9; UPCC analysis: 88% ee (Trefoil CEL2, CO_2/CH_3OH = 80/20, 1.0 mL/min, detector: 290 nm), Rt (major) = 9.1min, Rt (minor) = 10.6 min.

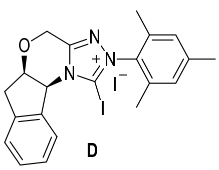

D

(5a*R*,10b*S*)-1-iodo-2-mesityl-5a,10b-dihydro-4H,6H-indeno[2,1-b][1,2,4]triazolo[4,3-d][1,4] oxazin-2-ium iodide (D)[17]: brown solid; ^{1}H NMR (400 MHz, DMSO-d_6) δ 7.48 (t, *J* = 6.8 Hz, 2H), 7.41 (t, *J* = 7.4 Hz, 1H), 7.35 (t, *J* = 7.4 Hz, 1H), 7.25 (s, 2H), 6.03 (d, *J* = 3.3 Hz, 1H), 5.27 (d, *J* = 16.3 Hz, 1H), 4.99 (d, *J* = 16.4 Hz, 1H), 4.91 (t, *J* = 3.8 Hz, 1H), 3.48 (dd, *J* = 16.8, 4.2 Hz, 1H), 3.12 (d, *J* = 16.7 Hz, 1H), 2.40 (s, 3H), 2.11 (s, 3H), 2.01 (s, 3H); ^{13}C NMR (100 MHz, DMSO-d_6) δ 154.0, 142.5, 141.6, 136.8, 136.0, 135.4, 132.1, 130.2, 130.0, 129.6, 127.6, 126.4, 125.0, 78.4, 63.3, 60.6, 37.2, 21.3, 17.5, 17.3; HRMS (ESI) for $C_{21}H_{21}IN_3O^+$ (M^+): calcd 458.0724, found 458.0719.

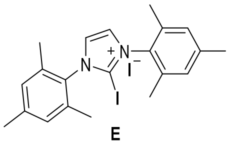

E

2-iodo-1,3-dimesityl-1H-imidazol-3-ium iodide (E)[18]: white solid; ^{1}H NMR (400 MHz, $CDCl_3$) δ 7.43 (s, 1H), 7.03 (s, 2H), 2.37 (s, 3H), 2.00 (s, 6H); ^{13}C NMR (100MHz, $CDCl_3$) δ 141.7, 134.2, 132.2, 130.1, 125.0, 118.9, 21.2, 17.6; HRMS (ESI) for $C_{21}H_{24}IN_2^+$ (M^+): calcd 431.0979, found 431.0974.

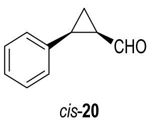

cis-**20**

***cis*-2-phenylcyclopropane-1-carbaldehyde (*cis*-20)[1]:** colorless oil; ^{1}H NMR (400 MHz, $CDCl_3$) δ 8.67 (d, *J* = 6.7 Hz, 1H), 7.36 – 7.19 (m, 5H), 2.83 (q, *J* = 8.2 Hz, 1H), 2.14 (ddd, *J* = 14.7, 8.1, 5.8 Hz, 1H), 1.88 (q, *J* = 5.6 Hz, 1H), 1.63 – 1.55 (m, 1H); ^{13}C NMR (100 MHz, $CDCl_3$) δ 201.5, 136.0, 129.4, 128.8, 127.4, 29.9, 26.6, 11.8.

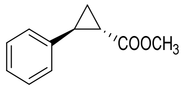

trans-**21**

methyl *trans*-2-phenylcyclopropane-1-carboxylate (*trans*-21)[2]: pale yellow oil; ^{1}H NMR (400 MHz, $CDCl_3$) δ 7.31 – 7.24 (m, 2H), 7.23 – 7.17 (m, 1H), 7.12 – 7.05 (m, 2H), 3.72 (s, 3H), 2.57 – 2.48 (m, 1H), 1.91 (dt, *J* = 9.1, 4.8 Hz, 1H), 1.61 (dt, *J* = 9.7, 4.9 Hz, 1H), 1.32 (ddd, *J* = 8.3, 6.5, 4.7 Hz, 1H); ^{13}C NMR (100 MHz, $CDCl_3$) δ 174.0, 140.2, 128.7, 126.7, 126.4, 52.1, 26.5, 24.1, 17.2.

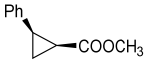

cis-**21**

methyl *cis*-2-phenylcyclopropane-1-carboxylate (*cis*-21)[2] : pale yellow oil; ^{1}H NMR (400 MHz, $CDCl_3$) δ 7.31 – 7.19 (m, 5H), 3.45 (s, 3H), 2.60 (q, *J* = 8.8 Hz, 1H), 2.11 (ddd, *J* = 9.2, 7.9, 5.6 Hz, 1H), 1.73 (dt, *J* = 7.5, 5.4 Hz, 1H), 1.36 (td, *J* = 8.0, 5.1 Hz, 1H); ^{13}C NMR (100 MHz, $CDCl_3$) δ 171.6, 136.6, 129.4, 128.1, 126.9, 51.6, 25.8, 21.8, 11.6.

3. Supplementary Figures

3.1 Cyclic Voltammetry

Supplementary Figure 1. Cyclic voltammetry in the mixture solvent of anhydrous CH_2Cl_2 (9 mL) containing 0.1 M *n*-Bu_4NBF_4.

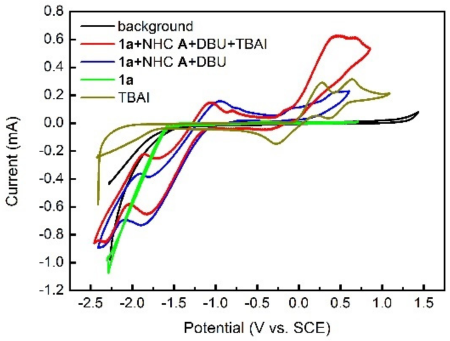

Note: background (black line); 0.3 mmol **1a** (green line); 0.1 mmol *n*-Bu_4NI (brown line); **1a** (0.3 mmol), NHC **A** (0.15 mmol, 50%), and DBU (0.15 mmol, 50%) were added to the mixture (blue line); **1a** (0.3 mmol), NHC **A** (0.15 mmol, 50%), DBU (0.15 mmol, 50%) and *n*-Bu_4NI (0.1 mmol) were added to the mixture (red line).

Supplementary Figure 2. Cyclic voltammetry in the mixture solvent of anhydrous CH_2Cl_2 (6 mL) and *t*-BuOH (3 mL) containing 0.1 M *n*-Bu_4NBF_4.

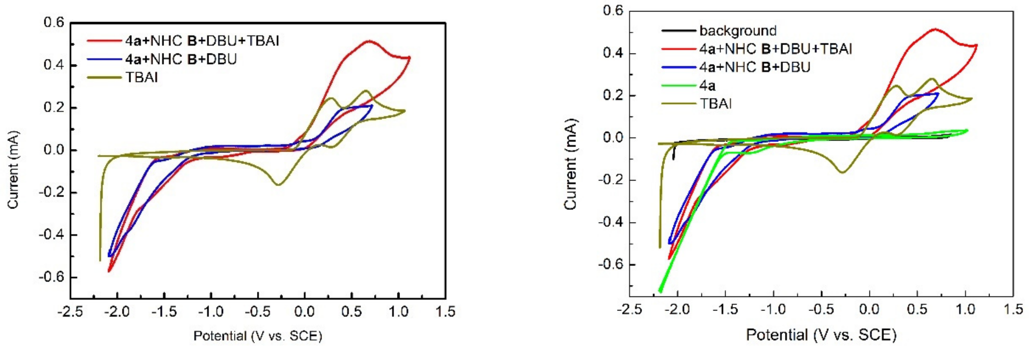

Note: background (black line); 0.3 mmol **4a** (green line); 0.1 mmol *n*-Bu_4NI (brown line); **4a** (0.3 mmol), NHC **B** (0.15 mmol, 50%), and DBU (0.15 mmol, 50%) were added to the mixture (blue line); **4a** (0.3 mmol), NHC **B** (0.15 mmol, 50%), DBU (0.15 mmol, 50%) and *n*-Bu_4NI (0.1 mmol) were added to the mixture (red line).

Supplementary Figure 3. Cyclic voltammetry in the mixture solvent of anhydrous CH_3CN (4.5 mL) and *t*-BuOH (4.5 mL) containing 0.1 M *n*-Bu_4NBF_4.

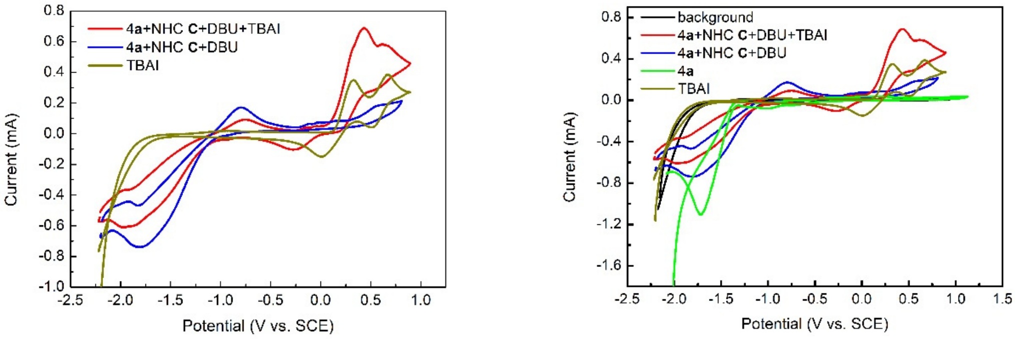

Note: background (black line); 0.3 mmol **4a** (green line); 0.1 mmol *n*-Bu_4NI (brown line); **4a** (0.3 mmol), NHC **C** (0.15 mmol, 50%), and DBU (0.15 mmol, 50%) were added to the mixture (blue line); **4a** (0.3 mmol), NHC **C** (0.15 mmol, 50%), DBU (0.15 mmol, 50%) and *n*-Bu_4NI (0.1 mmol) were added to the mixture (red line).

Supplementary Figure 4. Cyclic voltammetry in the mixture solvent of anhydrous DMF (6 mL) and DCE (3 mL) containing 0.1 M *n*-Bu_4NBF_4.

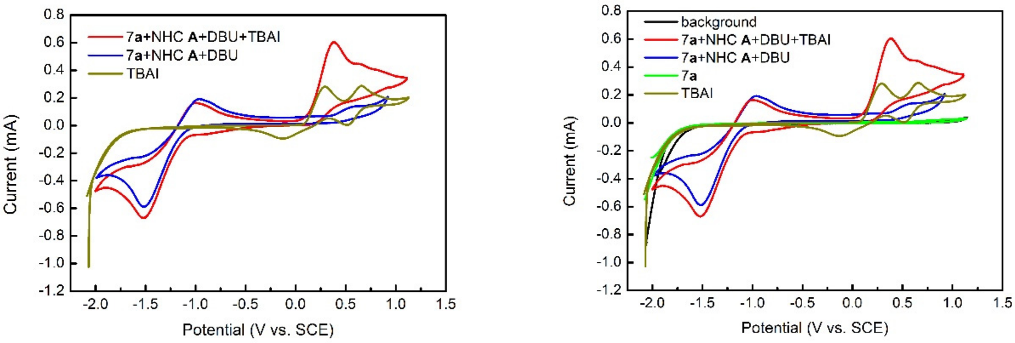

Note: background (black line); 0.3 mmol **7a** (green line); 0.1 mmol *n*-Bu_4NI (brown line); **7a** (0.3 mmol), NHC **A** (0.15 mmol, 50%), and DBU (0.15 mmol, 50%) were added to the mixture (blue line); **7a** (0.3 mmol), NHC **A** (0.15 mmol, 50%), DBU (0.15 mmol, 50%) and *n*-Bu_4NI (0.1 mmol) were added to the mixture (red line).

Supplementary Figure 5. Cyclic voltammetry in the mixture solvent of anhydrous THF (9 mL) containing 0.1 M *n*-Bu_4NBF_4.

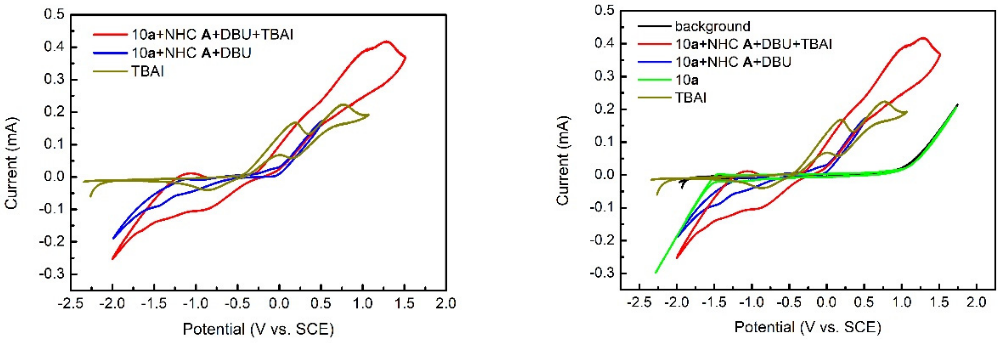

Note: background (black line); 0.3 mmol **10a** (green line); 0.1 mmol *n*-Bu_4NI (brown line); **10a** (0.3 mmol), NHC **A** (0.15 mmol, 50%), and DBU (0.15 mmol, 50%) were added to the mixture (blue line); **10a** (0.3 mmol), NHC **A** (0.15 mmol, 50%), DBU (0.15 mmol, 50%) and *n*-Bu_4NI (0.1 mmol) were added to the mixture (red line).

3.2 NMR spectra

400 MHz, 298 K, in $CDCl_3$

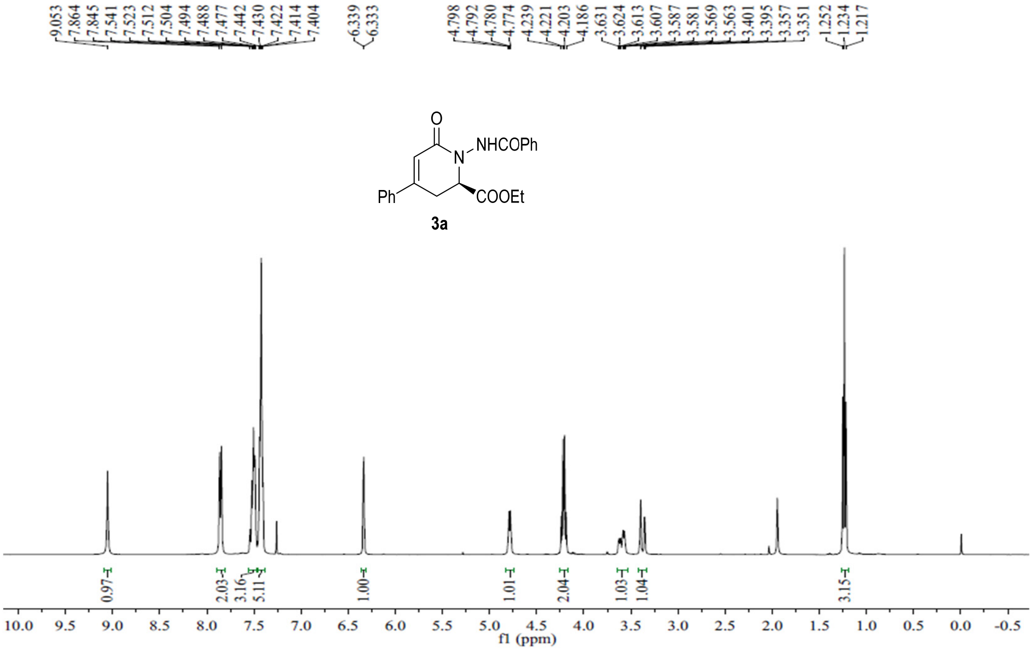

100 MHz, 298 K, in $CDCl_3$

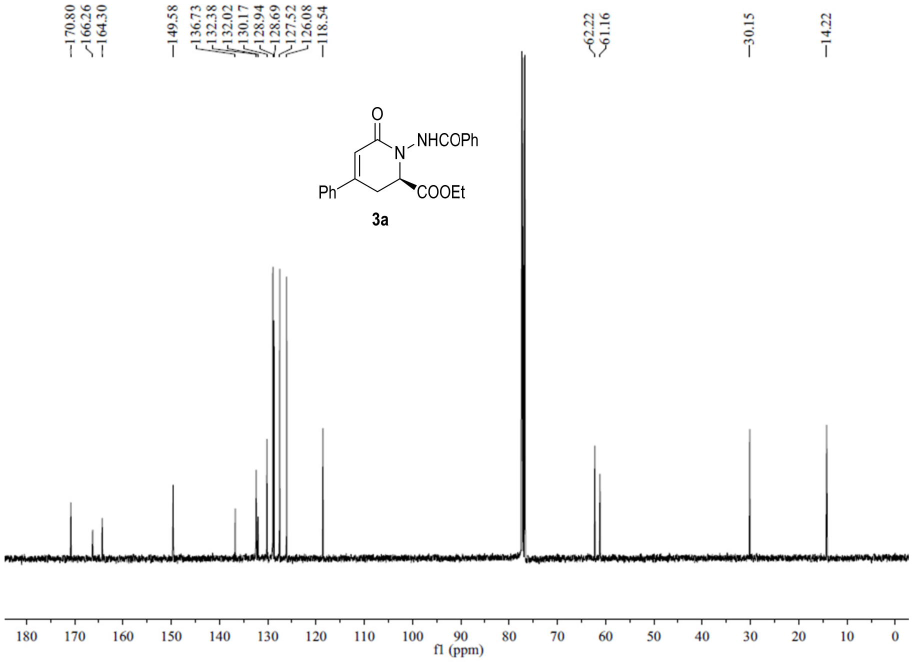

Supplementary Figure 6. ^{1}H and ^{13}C NMR spectra for compound **3a**.

400 MHz, 298 K, in $CDCl_3$

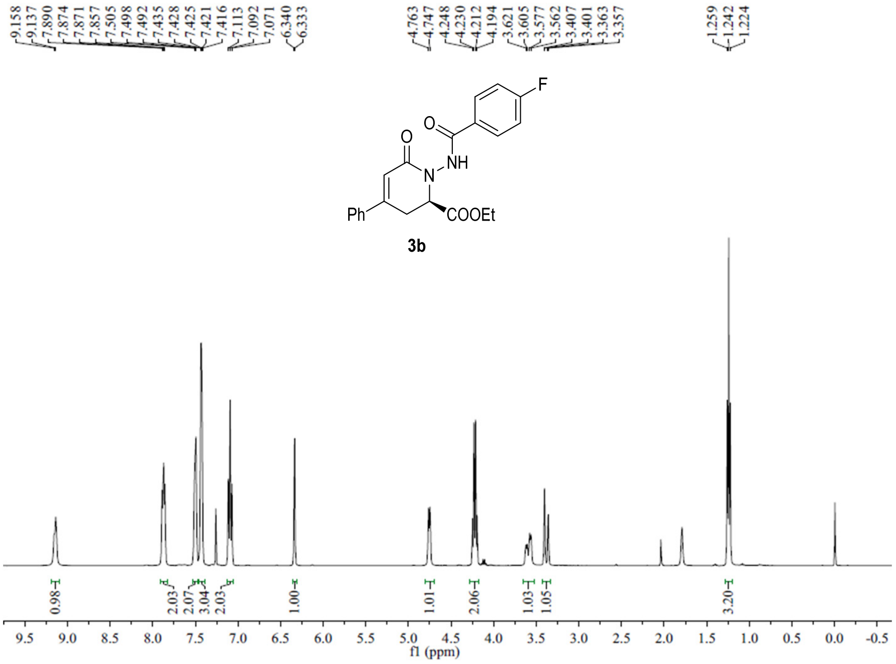

100 MHz, 298 K, in $CDCl_3$

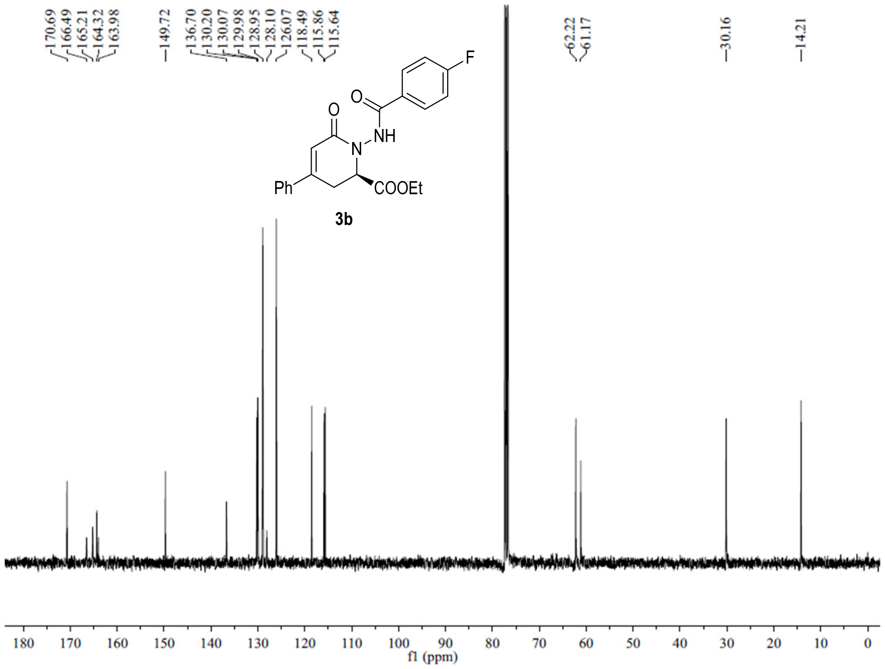

376 MHz, 298 K, in $CDCl_3$

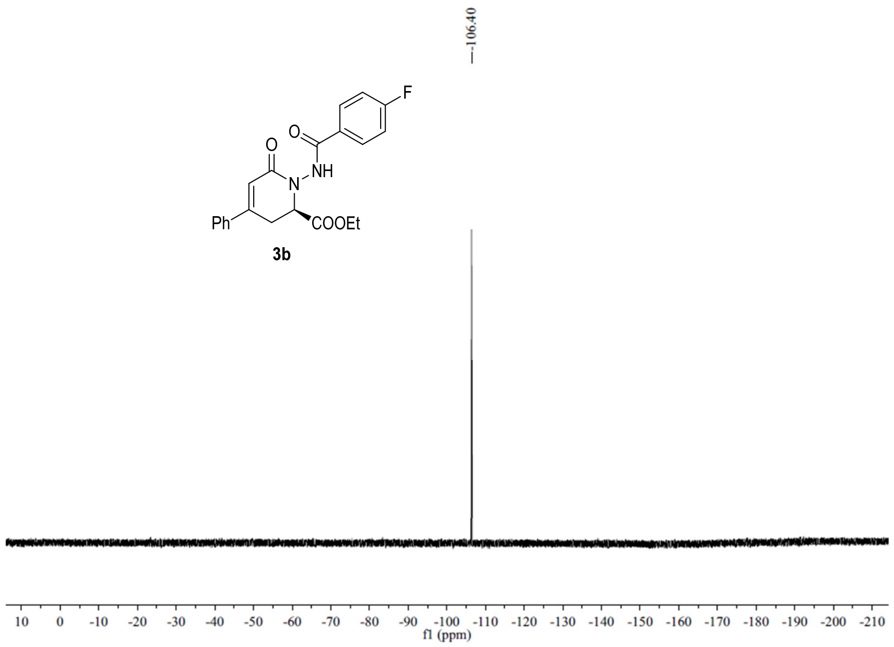

Supplementary Figure 7. ^{1}H and ^{13}C and ^{19}F NMR spectra for compound **3b**.

400 MHz, 298 K, in $CDCl_3$

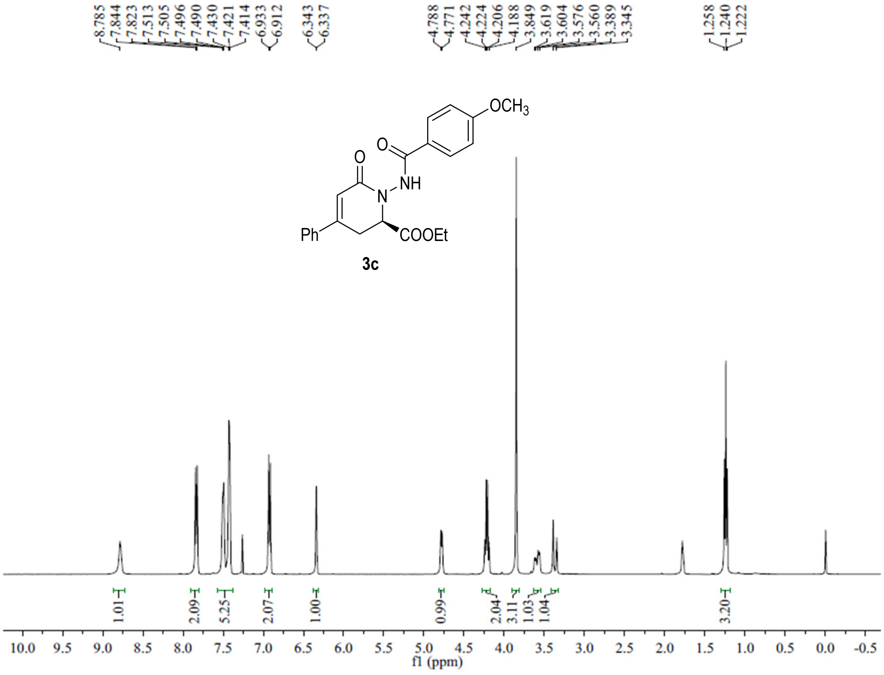

100 MHz, 298 K, in $CDCl_3$

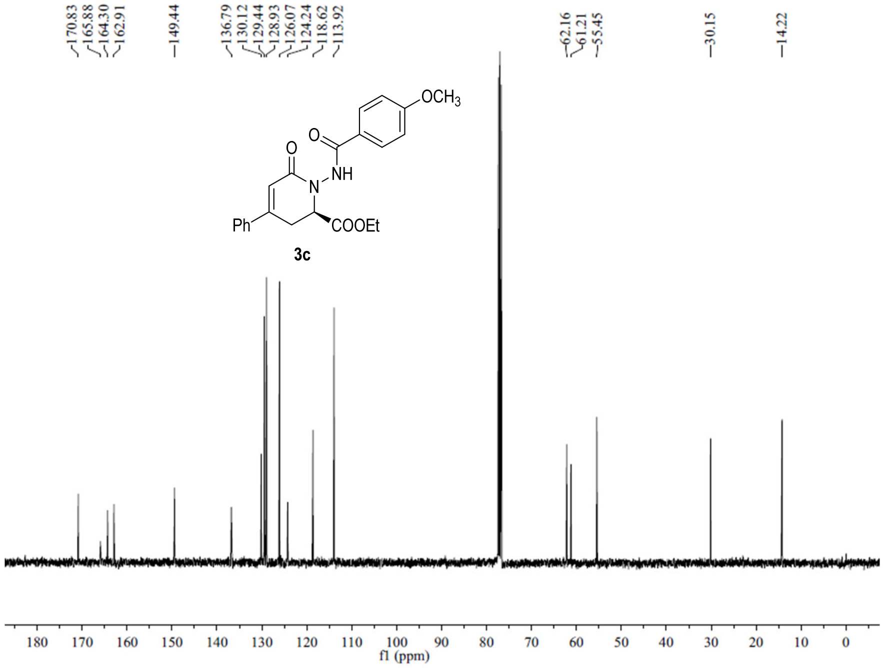

Supplementary Figure 8. ^{1}H and ^{13}C NMR spectra for compound **3c**.

400 MHz, 298 K, in CDCl$_3$

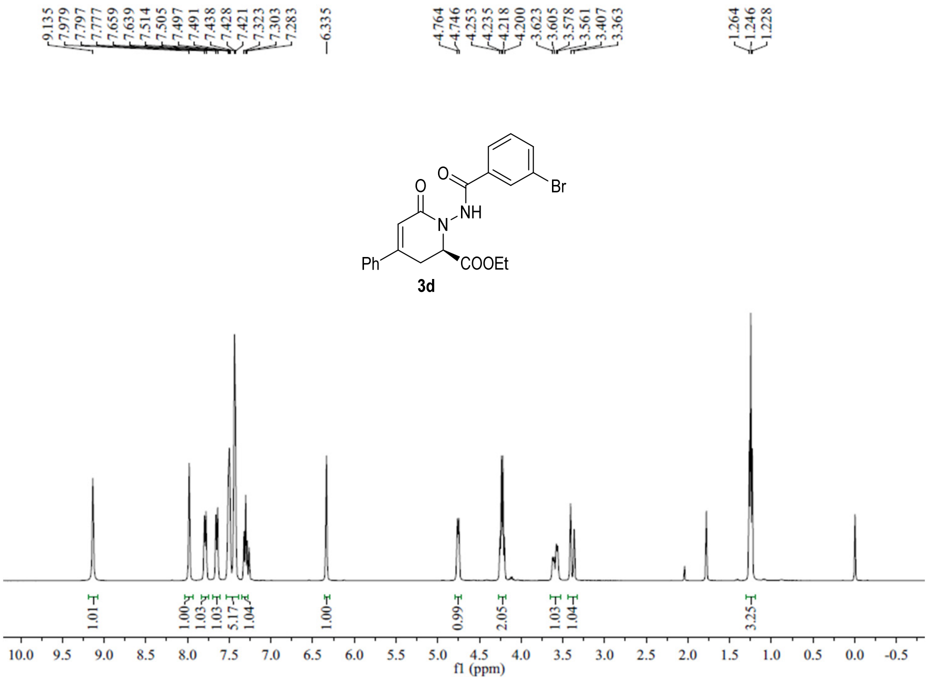

100 MHz, 298 K, in CDCl$_3$

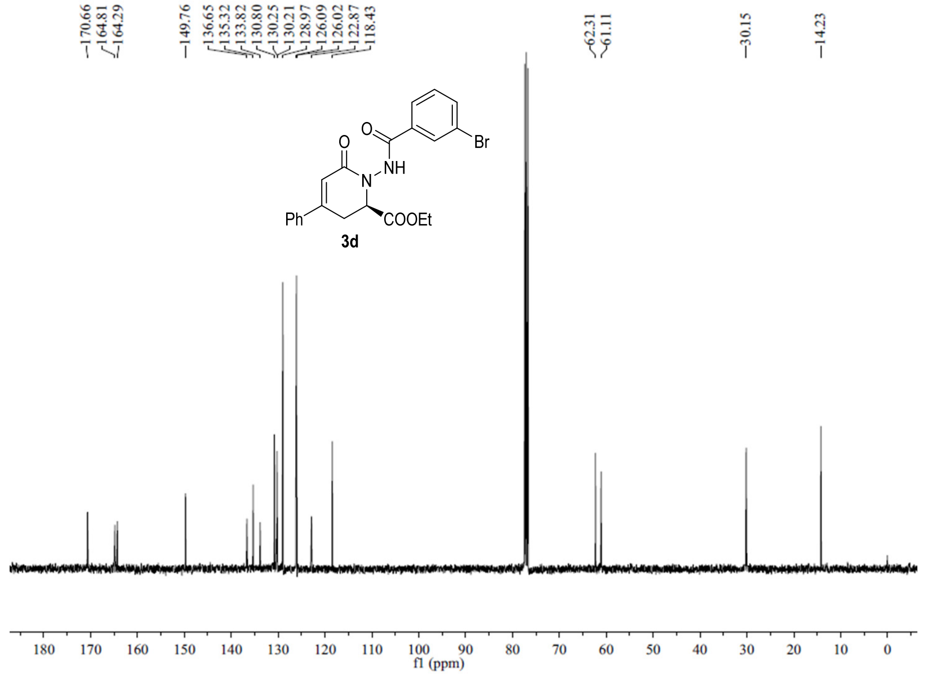

Supplementary Figure 9. ^{1}H and ^{13}C NMR spectra for compound **3d**.

400 MHz, 298 K, in $CDCl_3$

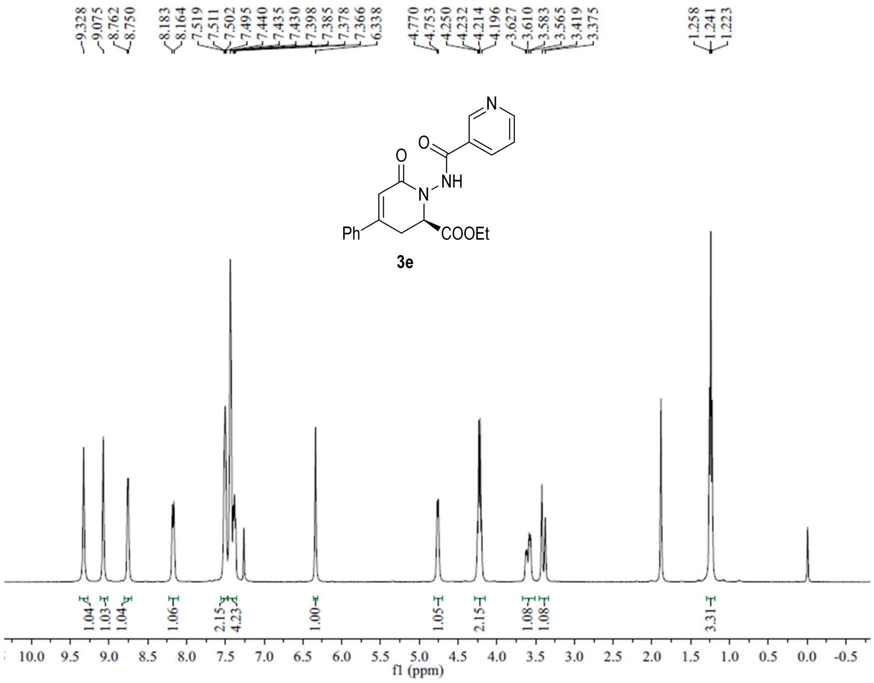

100 MHz, 298 K, in $CDCl_3$

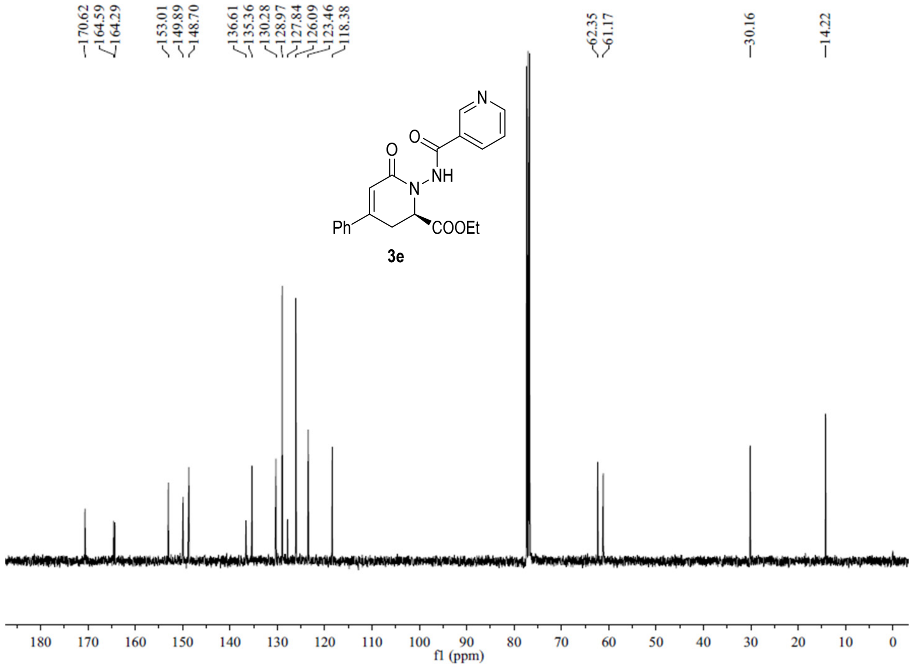

Supplementary Figure 10. ^{1}H and ^{13}C NMR spectra for compound **3e**.

400 MHz, 298 K, in $CDCl_3$

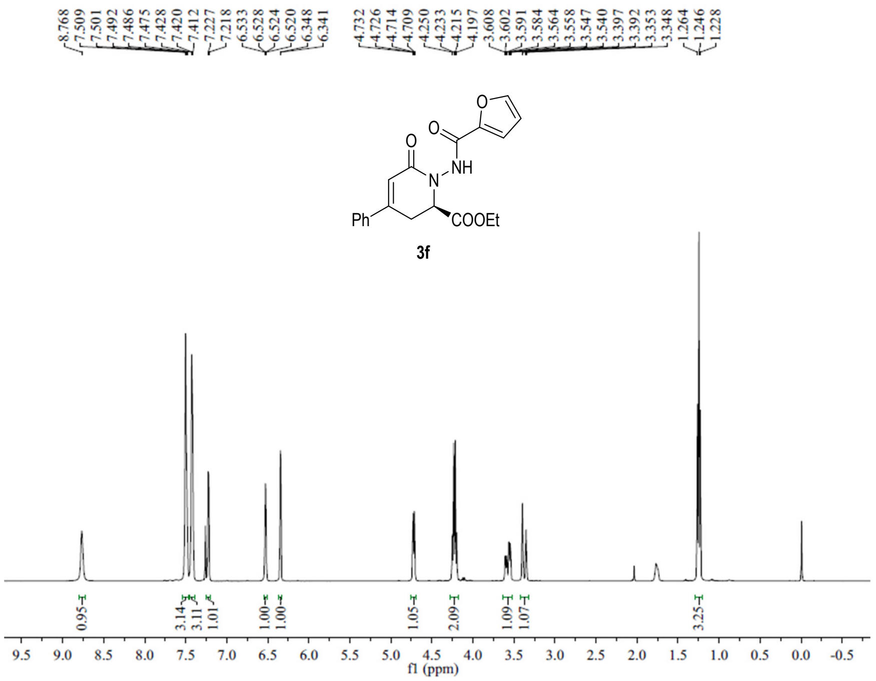

100 MHz, 298 K, in $CDCl_3$

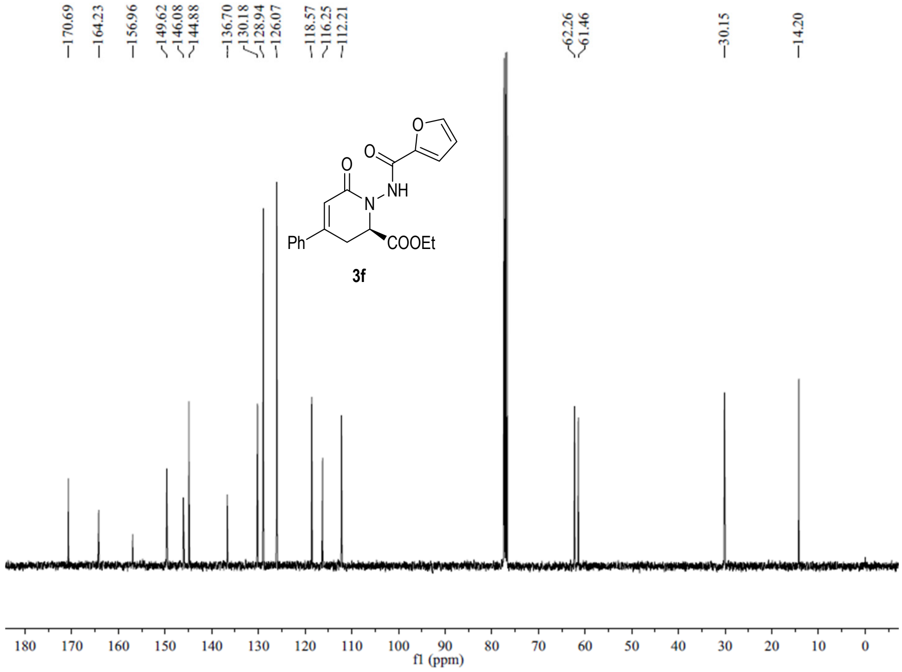

Supplementary Figure 11. ^{1}H and ^{13}C NMR spectra for compound **3f**.

400 MHz, 298 K, in CDCl$_3$

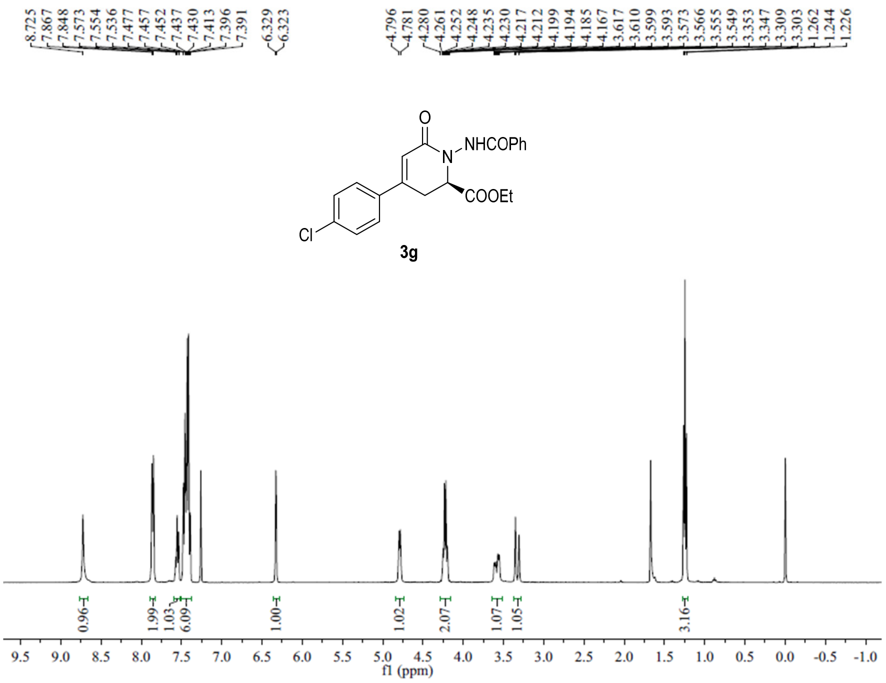

100 MHz, 298 K, in CDCl$_3$

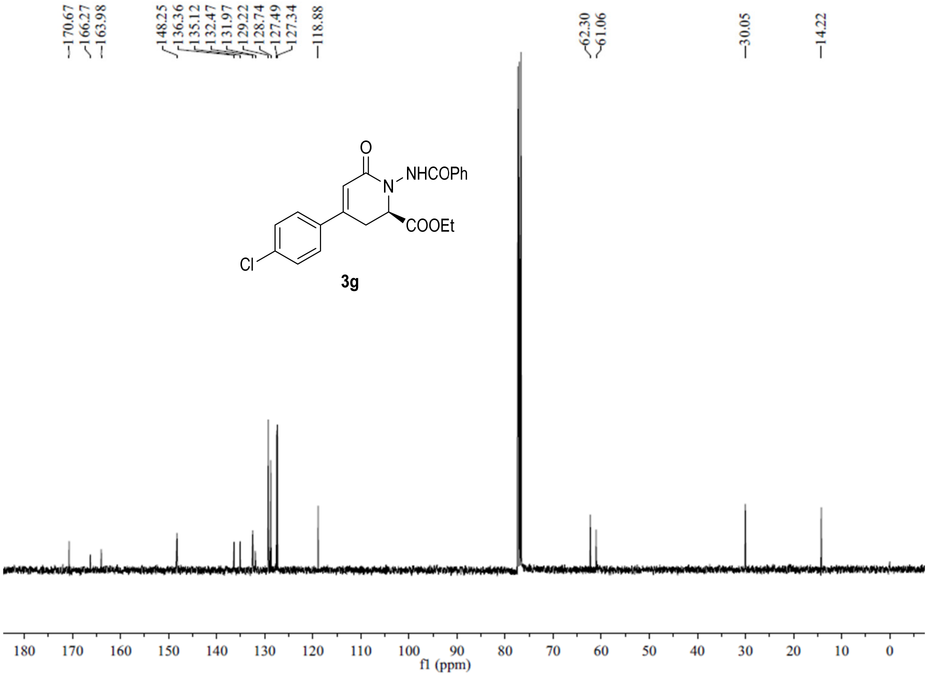

Supplementary Figure 12. ^{1}H and ^{13}C NMR spectra for compound **3g**.

400 MHz, 298 K, in $CDCl_3$

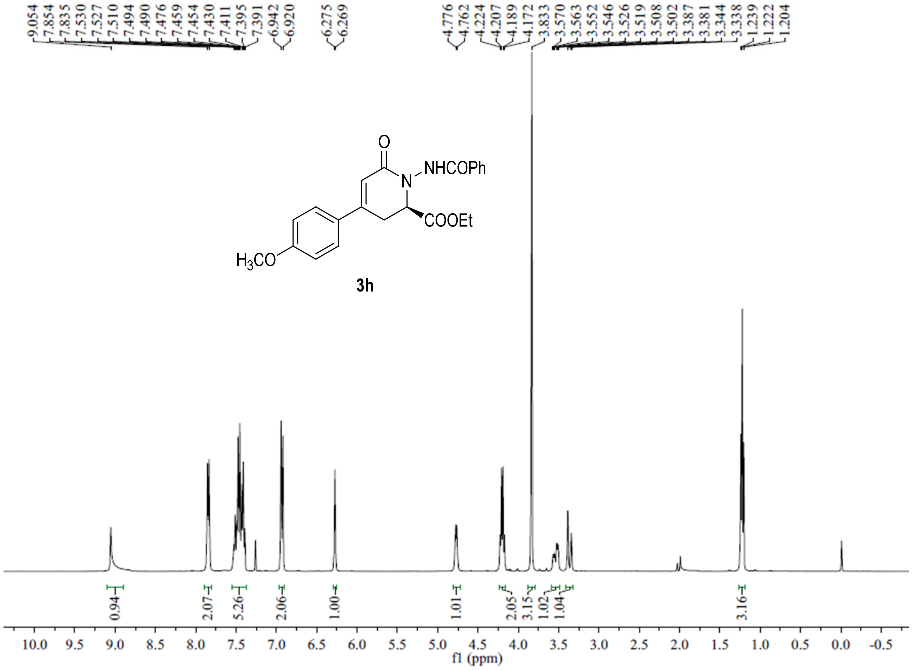

100 MHz, 298 K, in $CDCl_3$

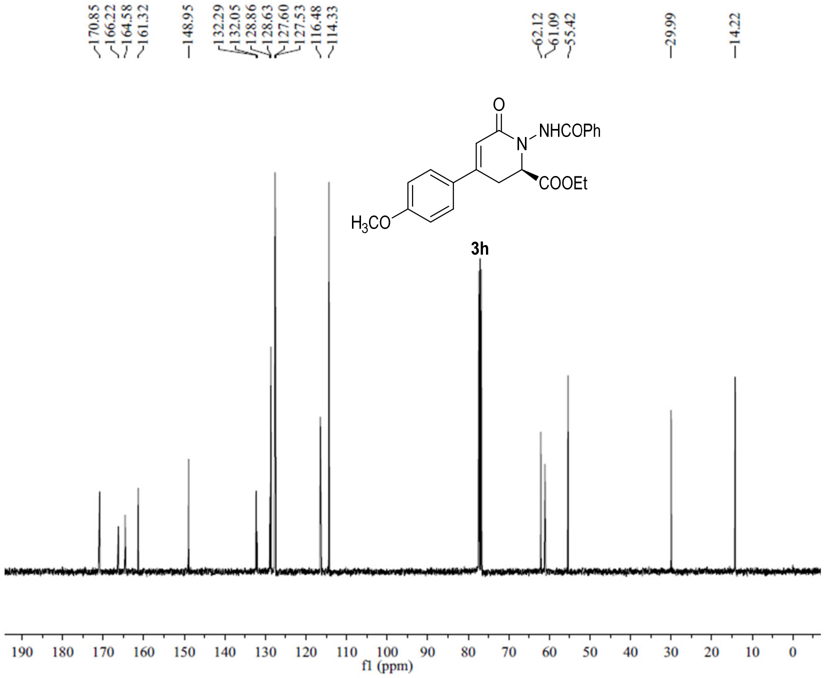

Supplementary Figure 13. ^{1}H and ^{13}C NMR spectra for compound **3h**.

400 MHz, 298 K, in $CDCl_3$

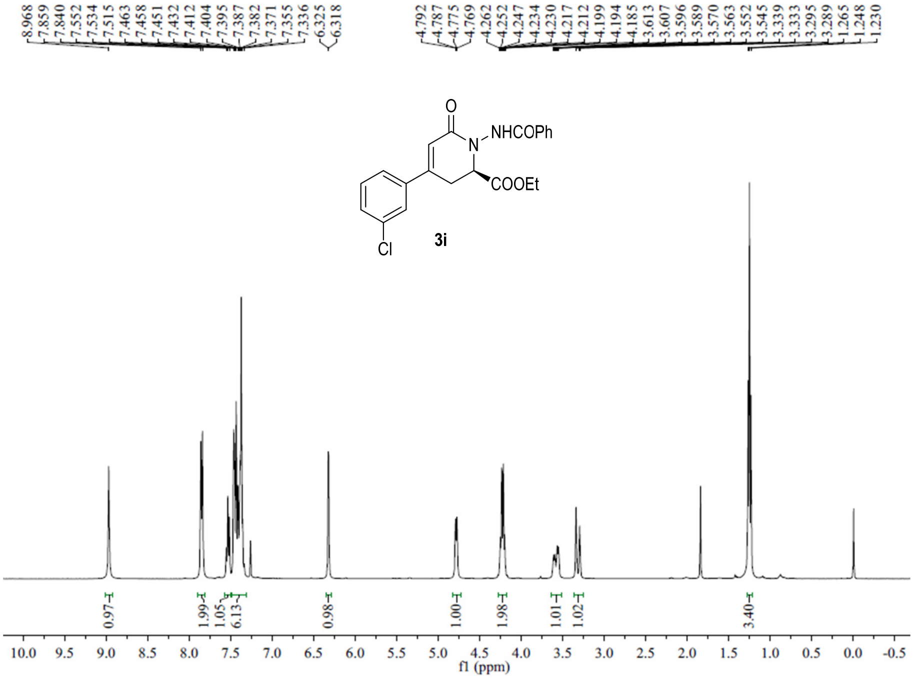

100 MHz, 298 K, in $CDCl_3$

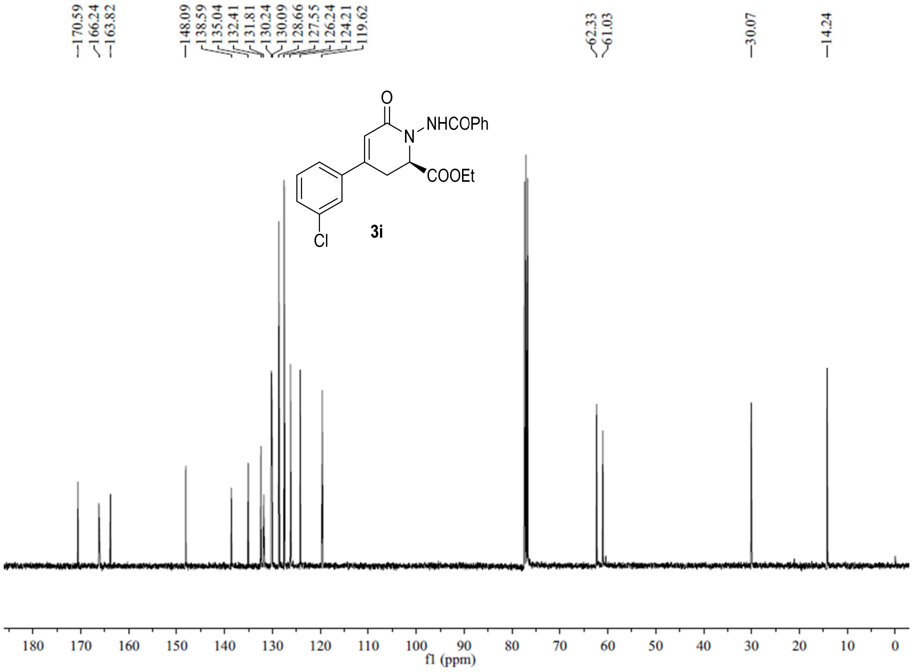

Supplementary Figure 14. ^{1}H and ^{13}C NMR spectra for compound **3i**.

400 MHz, 298 K, in $CDCl_3$

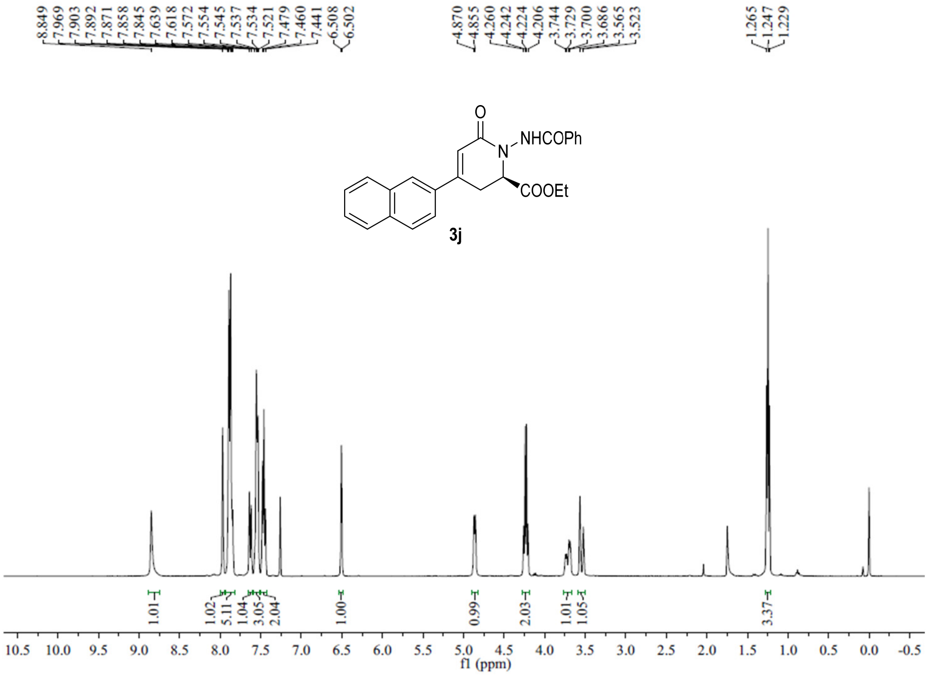

100 MHz, 298 K, in $CDCl_3$

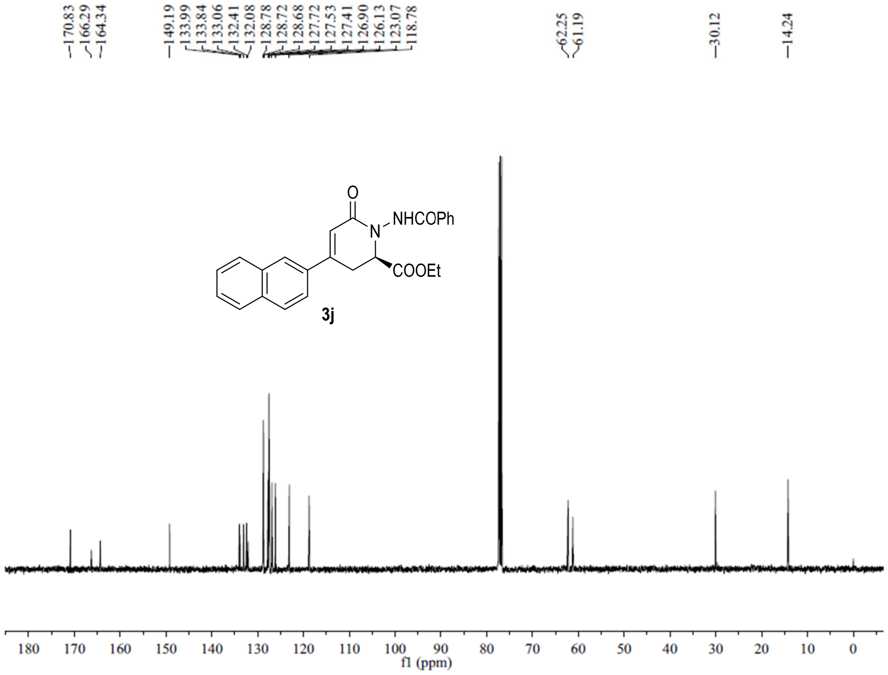

Supplementary Figure 15. ^{1}H and ^{13}C NMR spectra for compound **3j**.

400 MHz, 298 K, in $CDCl_3$

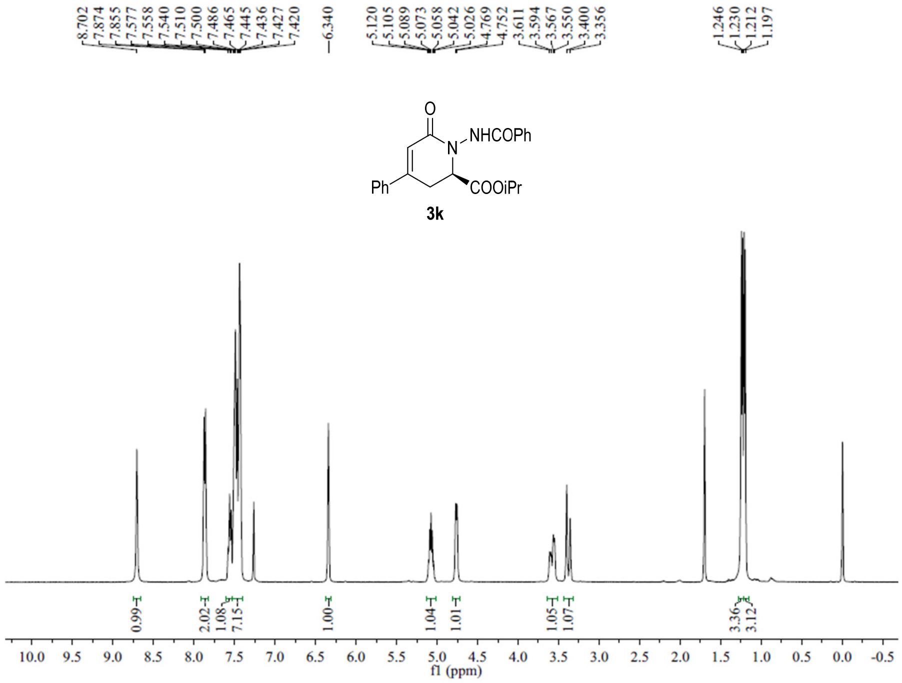

100 MHz, 298 K, in $CDCl_3$

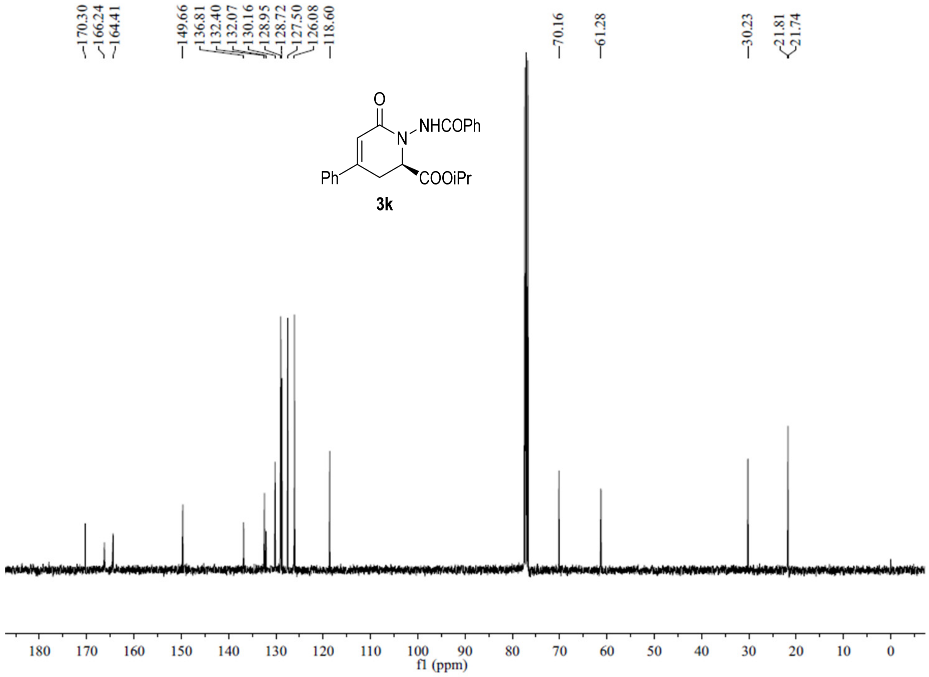

Supplementary Figure 16. ^{1}H and ^{13}C NMR spectra for compound **3k**.

400 MHz, 298 K, in $CDCl_3$

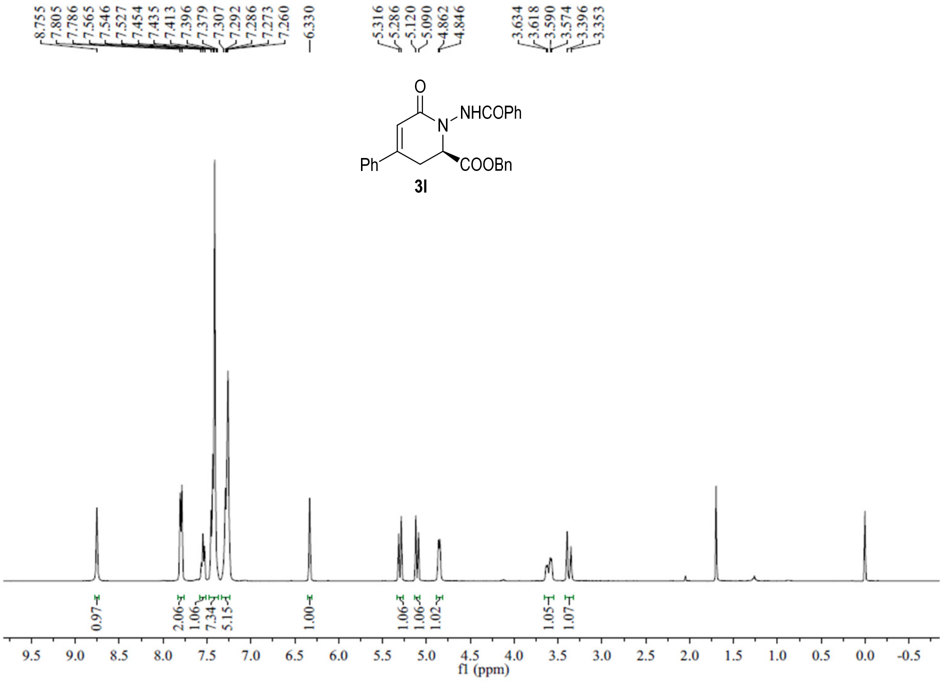

100 MHz, 298 K, in $CDCl_3$

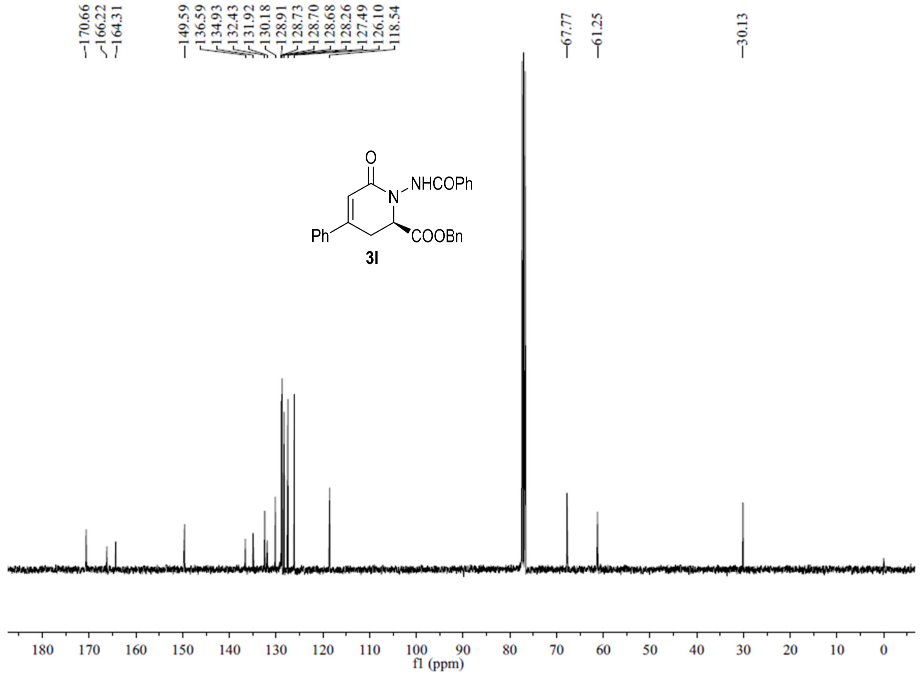

Supplementary Figure 17. ^{1}H and ^{13}C NMR spectra for compound **3l**.

400 MHz, 298 K, in $CDCl_3$

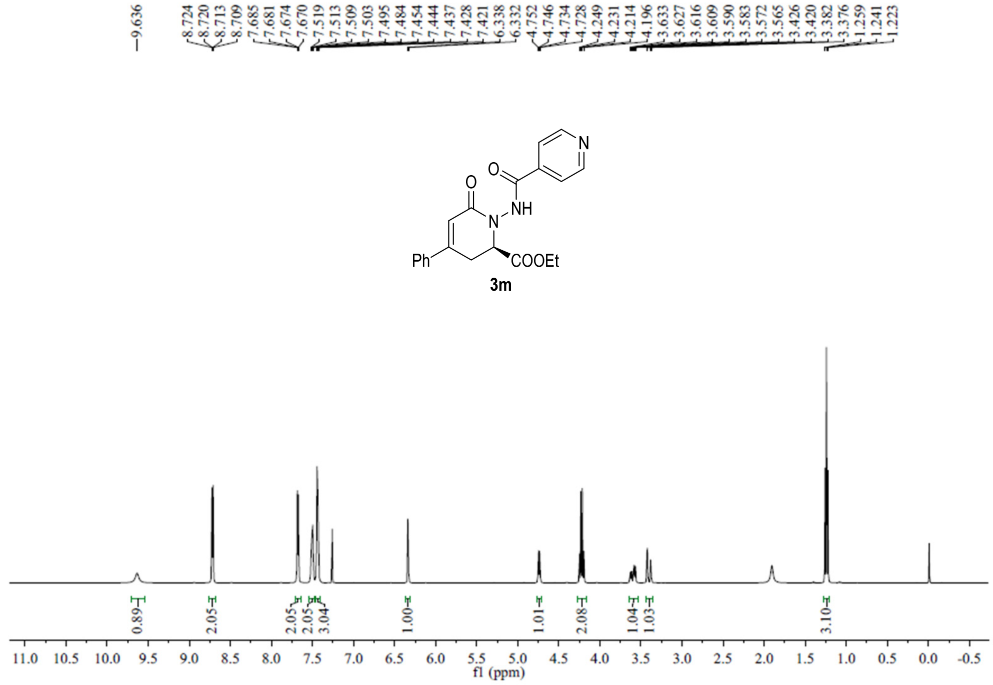

100 MHz, 298 K, in $CDCl_3$

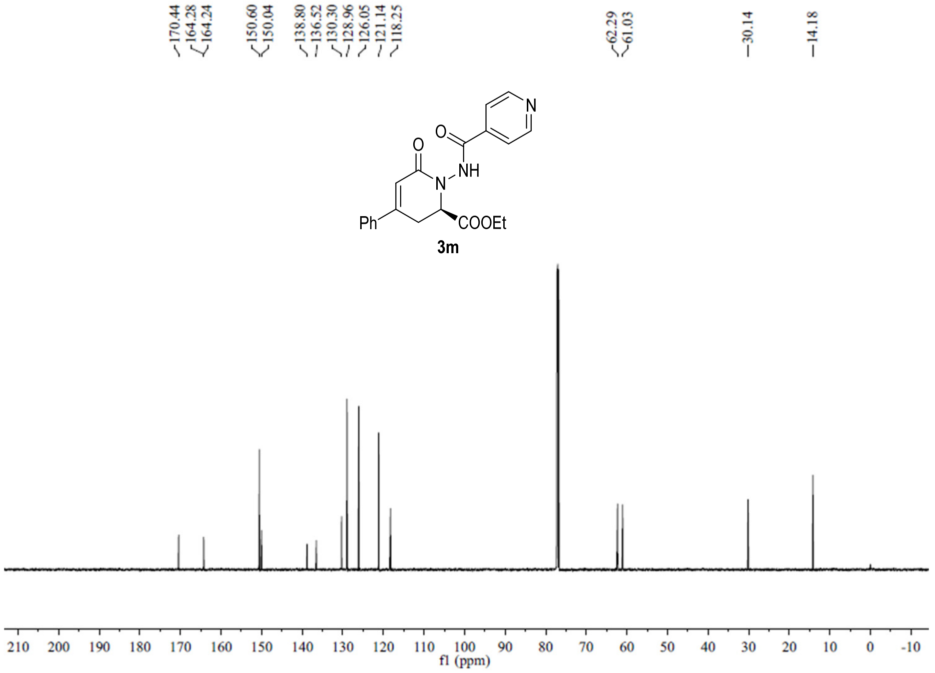

Supplementary Figure 18. ^{1}H and ^{13}C NMR spectra for compound **3m**.

400 MHz, 298 K, in $CDCl_3$

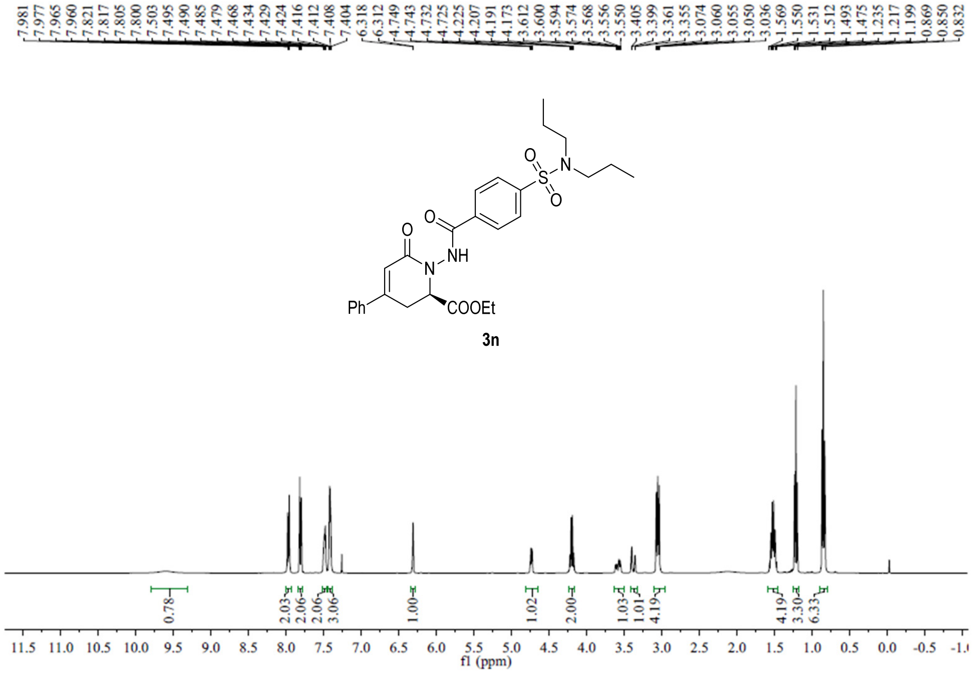

100 MHz, 298 K, in $CDCl_3$

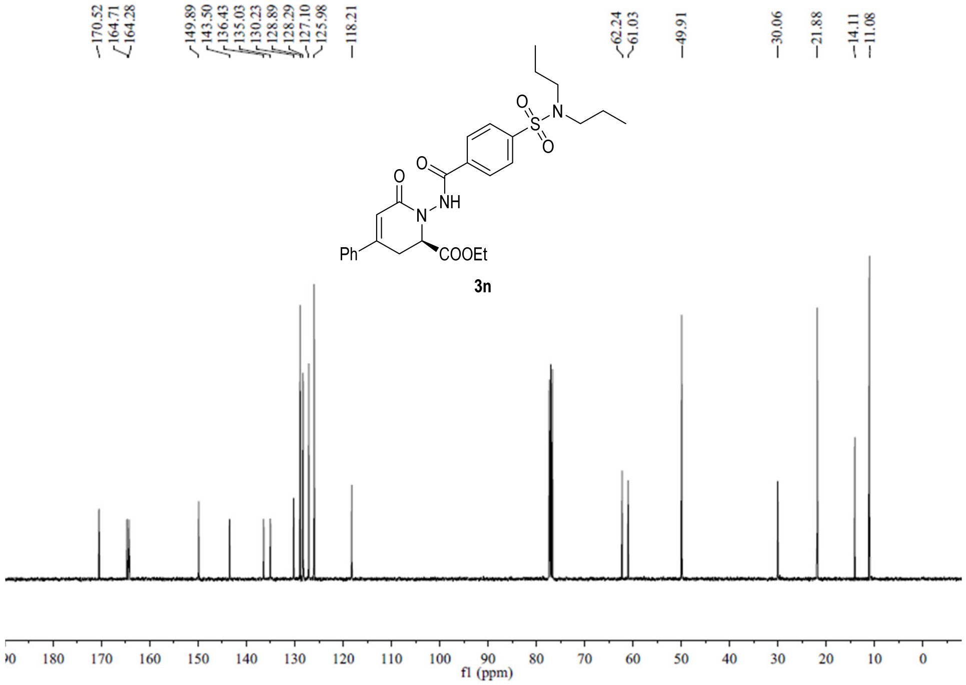

Supplementary Figure 19. ^{1}H and ^{13}C NMR spectra for compound **3n**.

400 MHz, 298 K, in $CDCl_3$

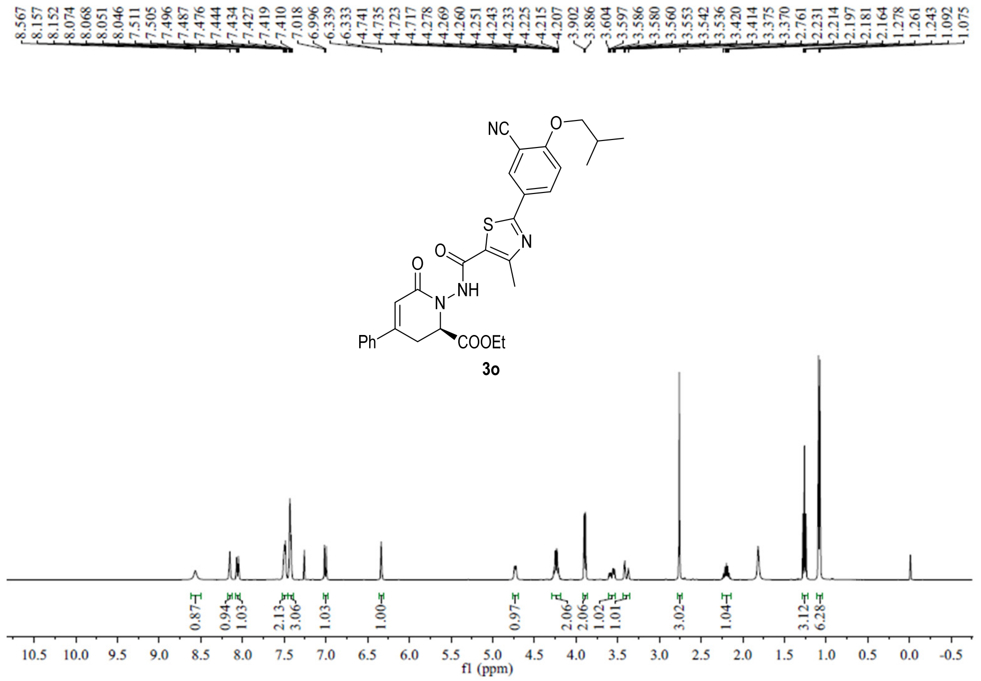

100 MHz, 298 K, in $CDCl_3$

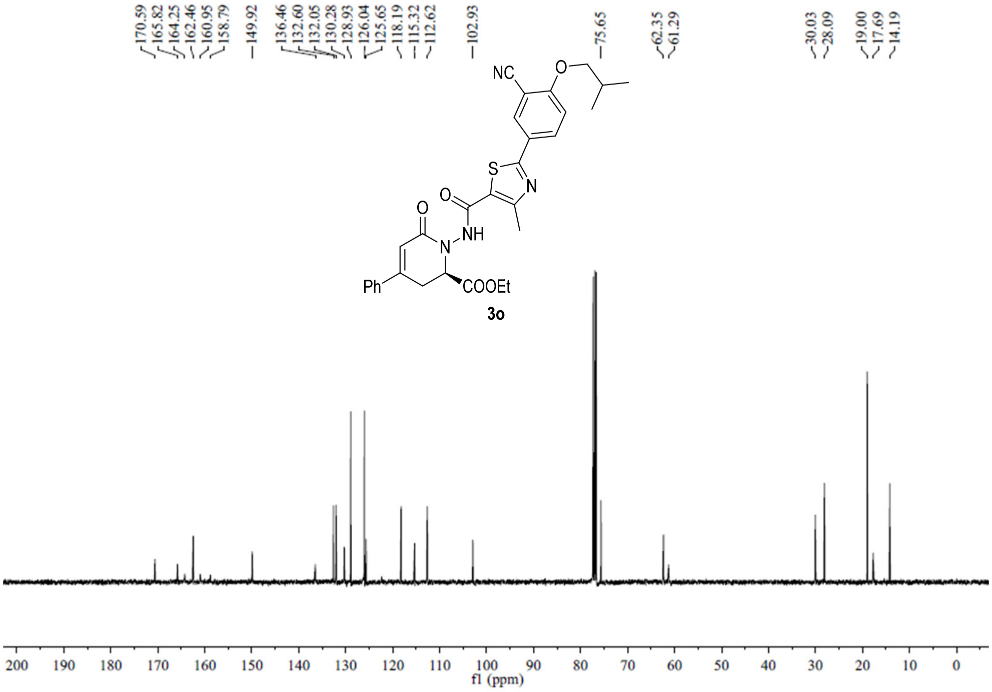

Supplementary Figure 20. ^{1}H and ^{13}C NMR spectra for compound **3o**.

400 MHz, 298 K, in $CDCl_3$

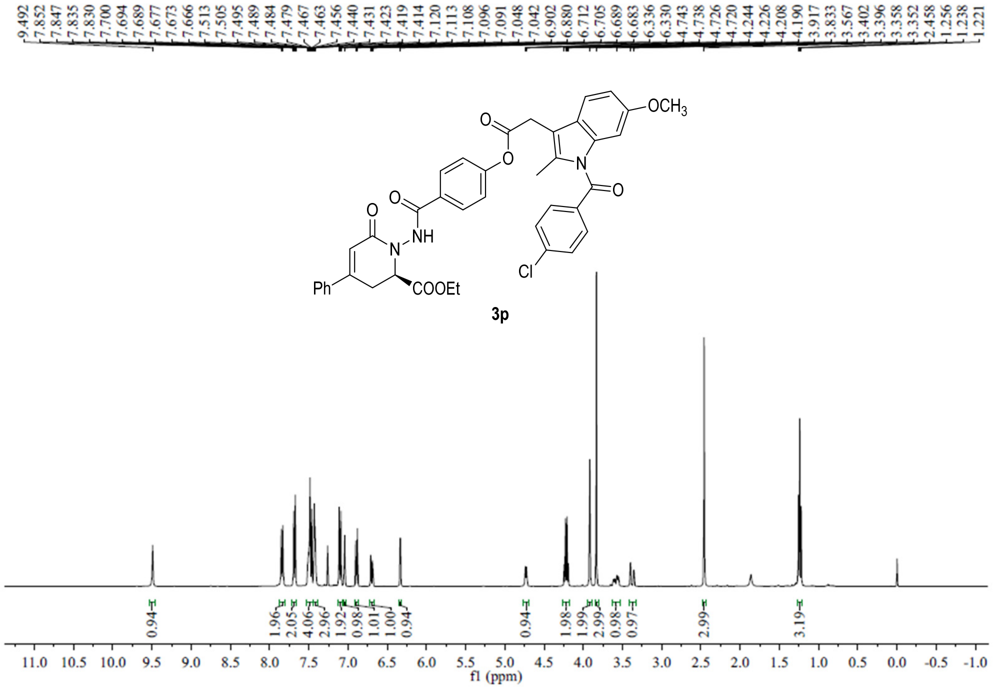

100 MHz, 298 K, in $CDCl_3$

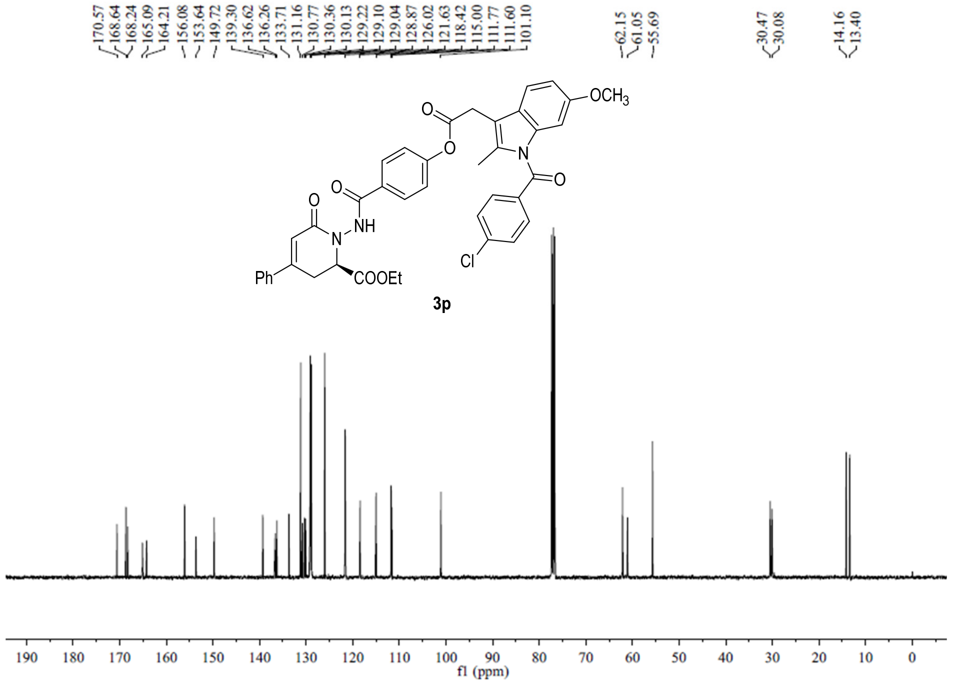

Supplementary Figure 21. ^{1}H and ^{13}C NMR spectra for compound **3p**.

400 MHz, 298 K, in $CDCl_3$

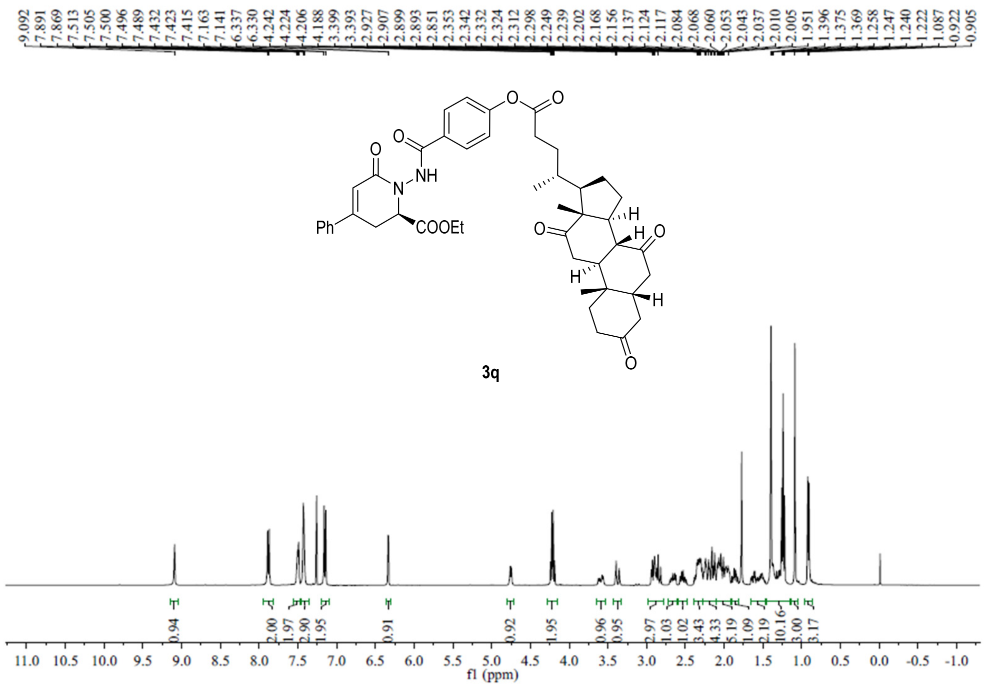

100 MHz, 298 K, in $CDCl_3$

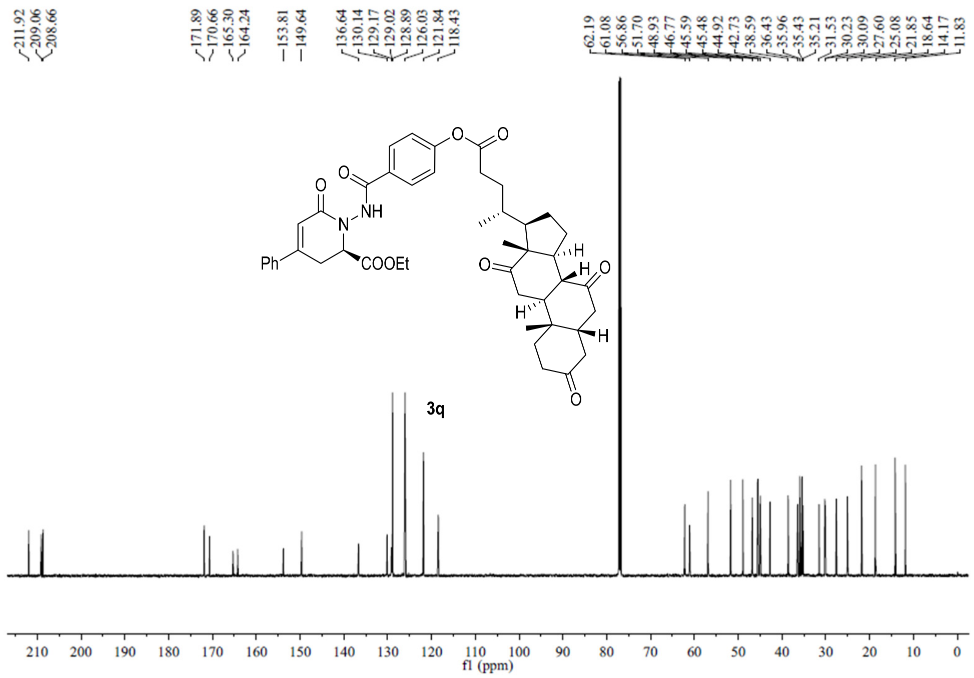

Supplementary Figure 22. ^{1}H and ^{13}C NMR spectra for compound **3q**.

400 MHz, 298 K, in $CDCl_3$

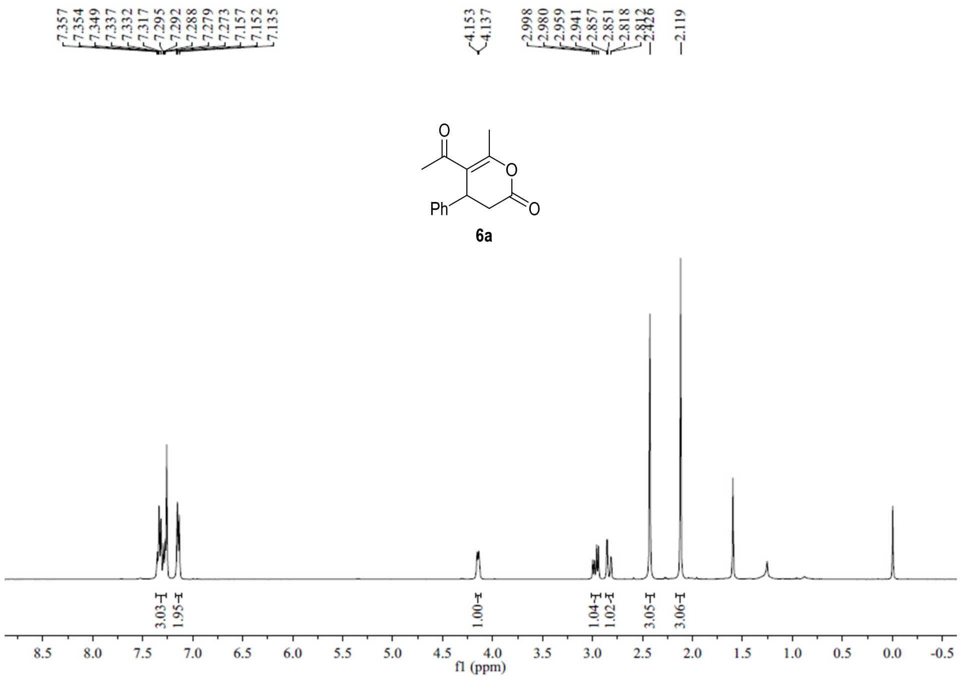

100 MHz, 298 K, in $CDCl_3$

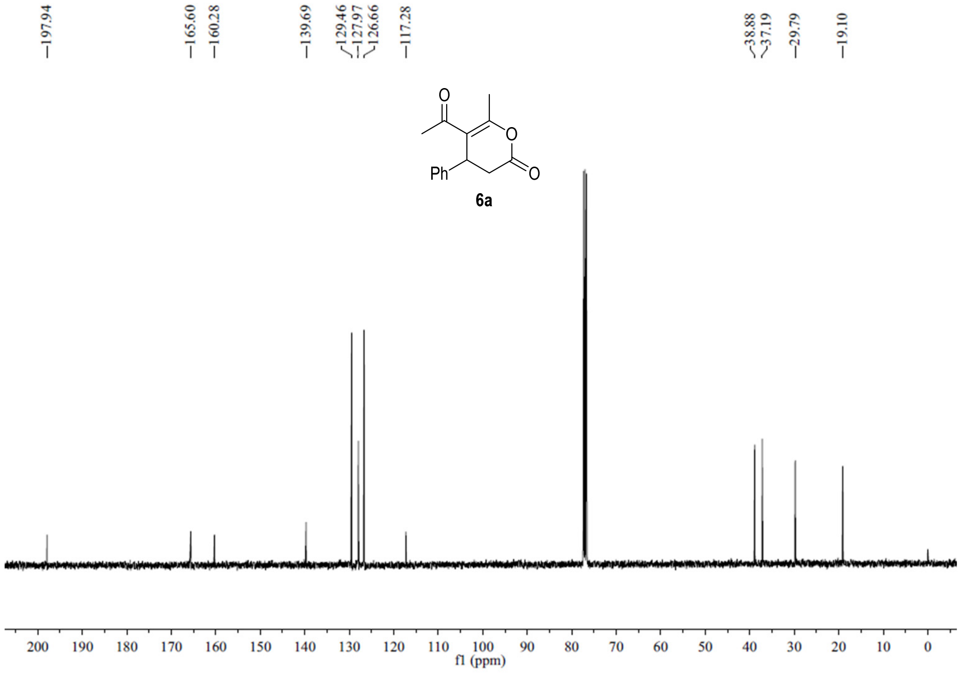

Supplementary Figure 23. ^{1}H and ^{13}C NMR spectra for compound **6a**.

400 MHz, 298 K, in $CDCl_3$

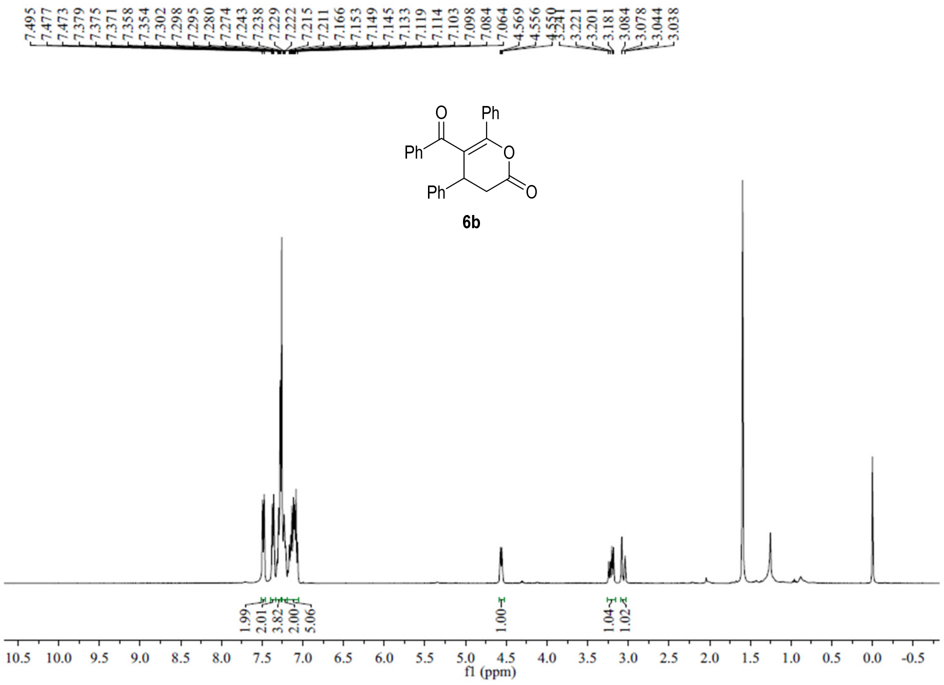

100 MHz, 298 K, in $CDCl_3$

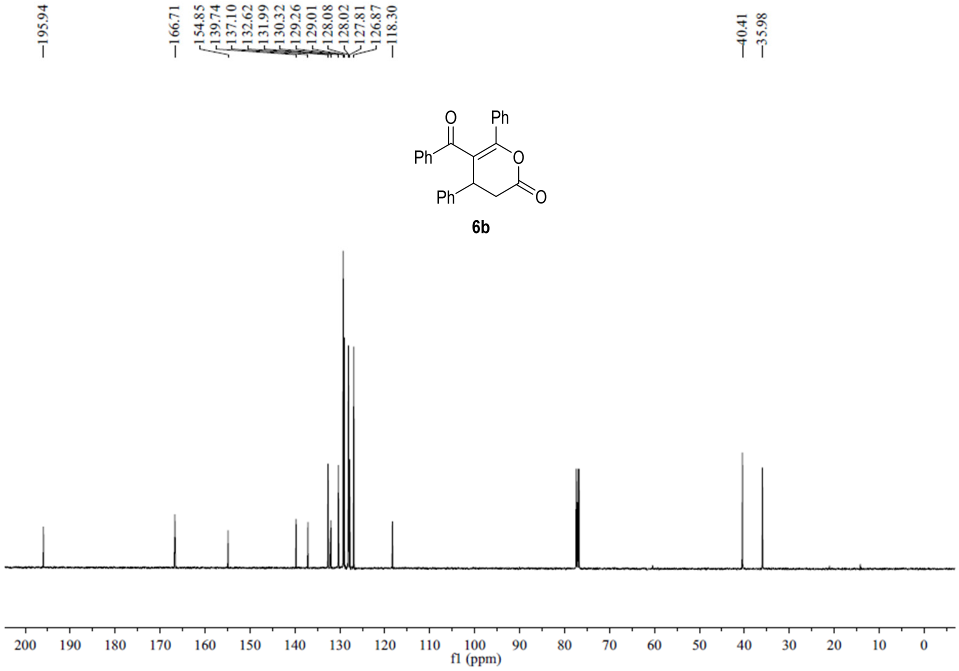

Supplementary Figure 24. ^{1}H and ^{13}C NMR spectra for compound **6b**.

400 MHz, 298 K, in $CDCl_3$

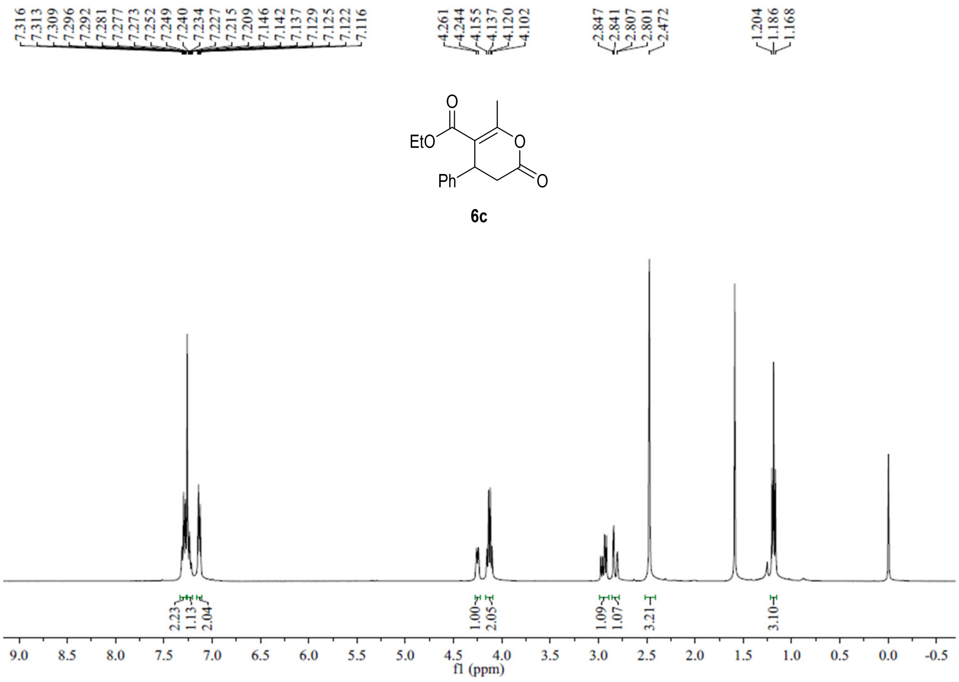

100 MHz, 298 K, in $CDCl_3$

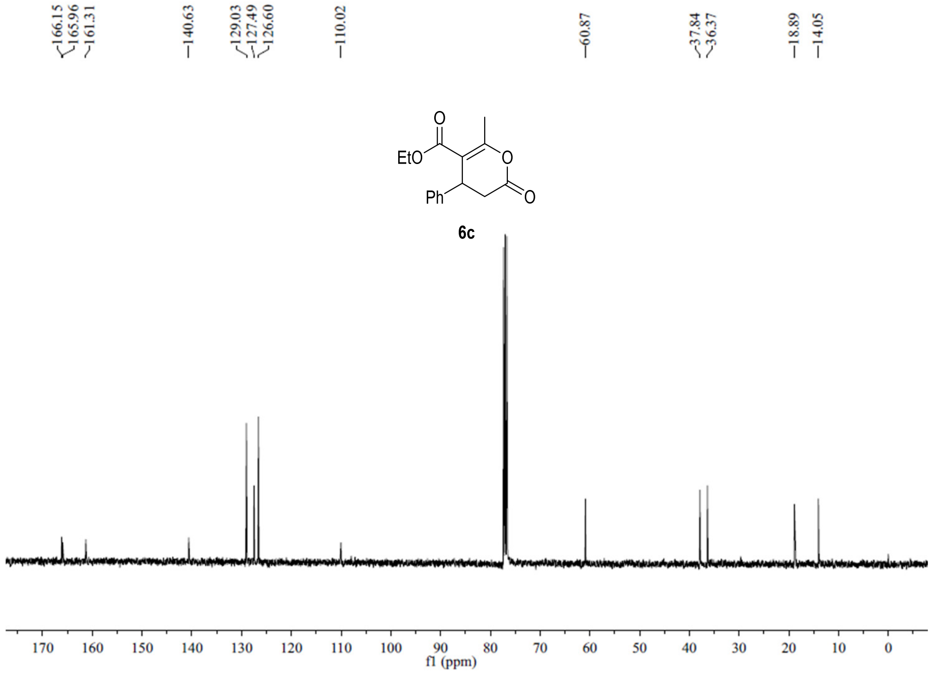

Supplementary Figure 25. ^{1}H and ^{13}C NMR spectra for compound **6c**.

400 MHz, 298 K, in $CDCl_3$

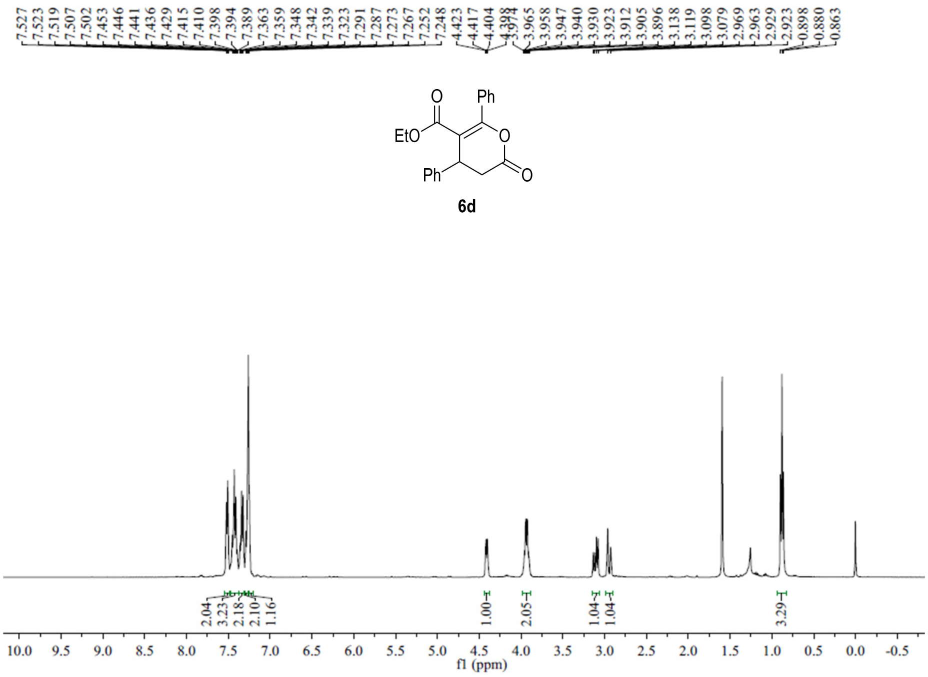

100 MHz, 298 K, in $CDCl_3$

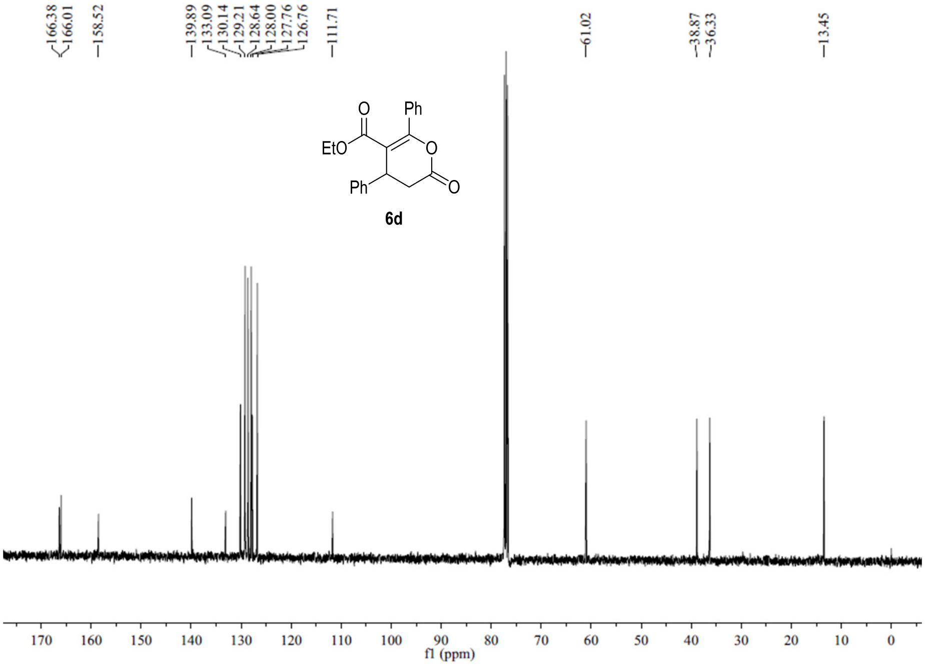

Supplementary Figure 26. ^{1}H and ^{13}C NMR spectra for compound **6d**.

400 MHz, 298 K, in CDCl3

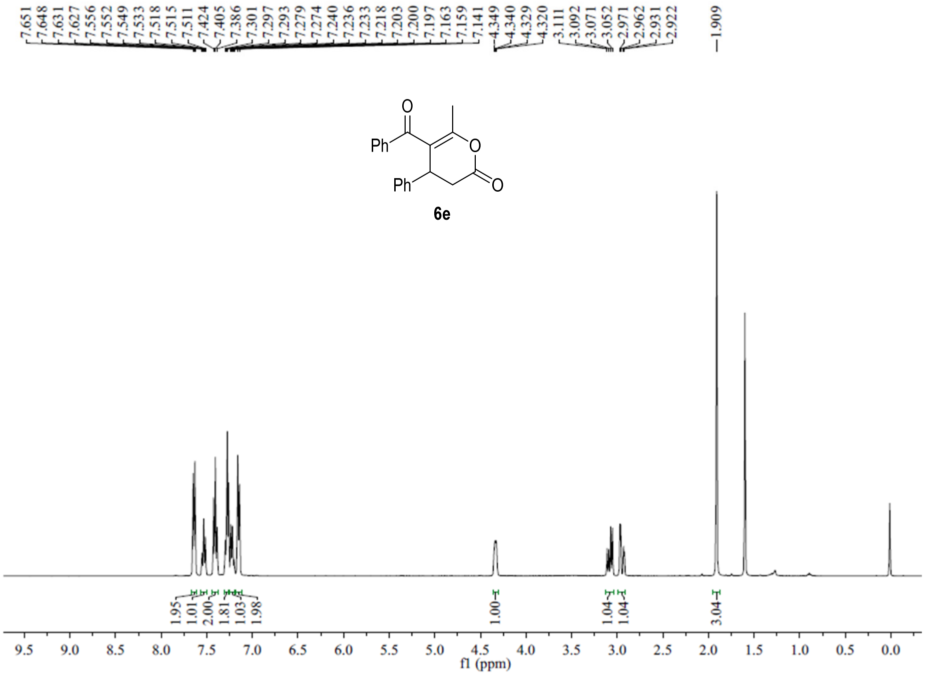

100 MHz, 298 K, in CDCl3

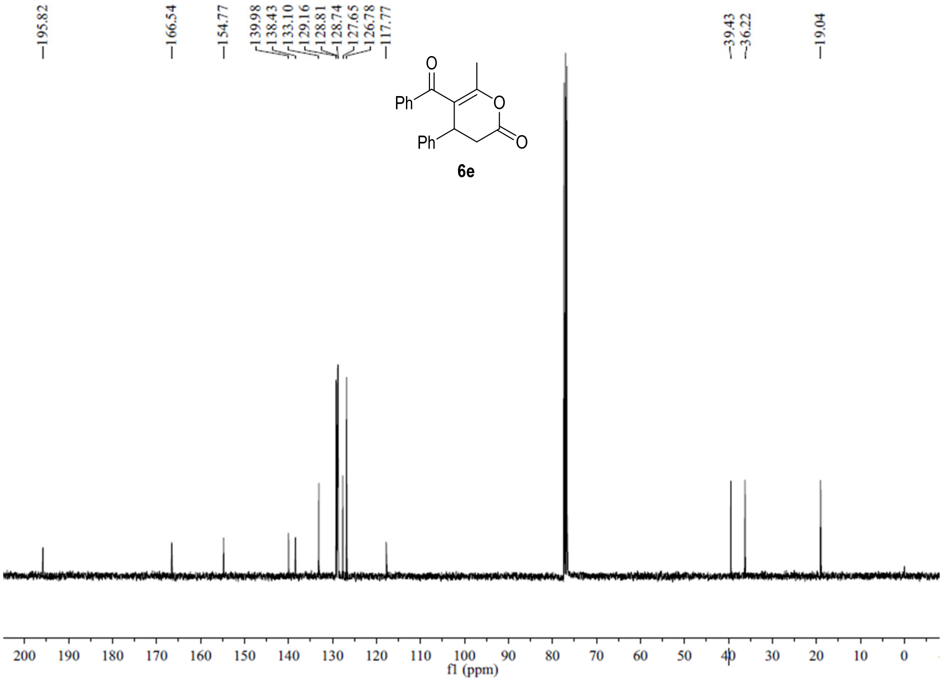

Supplementary Figure 27. ^{1}H and ^{13}C NMR spectra for compound **6e**.

400 MHz, 298 K, in CDCl3

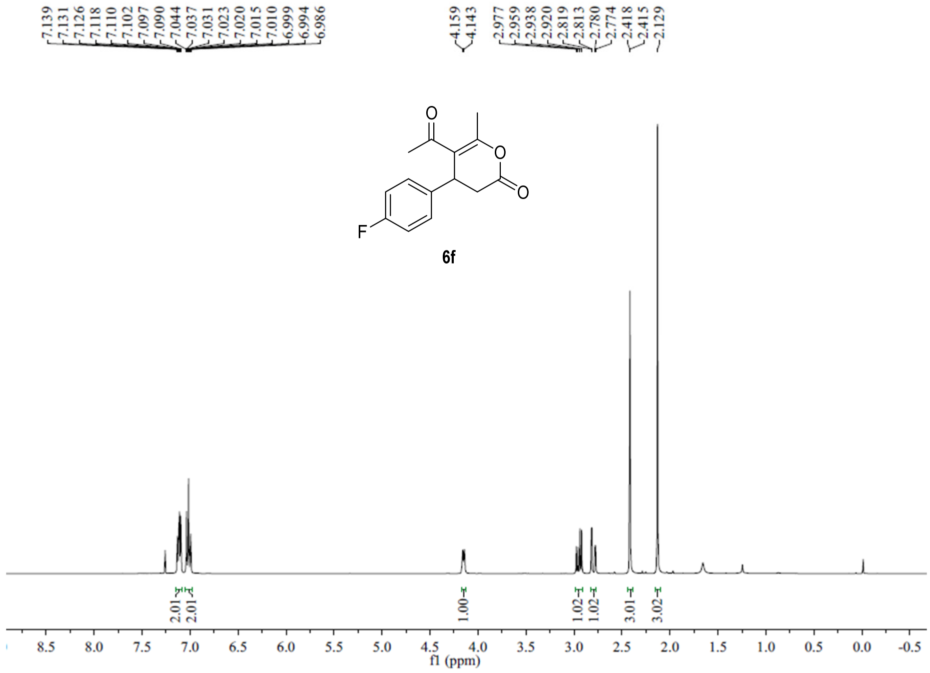

100 MHz, 298 K, in CDCl3

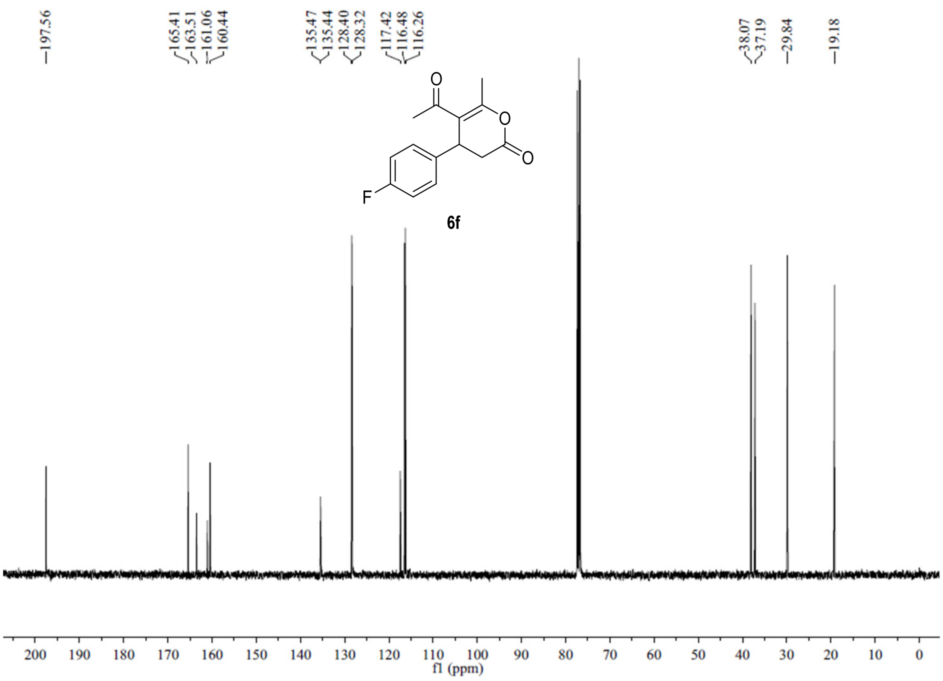

Supplementary Figure 28. ^{1}H and ^{13}C NMR spectra for compound **6f**.

400 MHz, 298 K, in CDCl$_3$

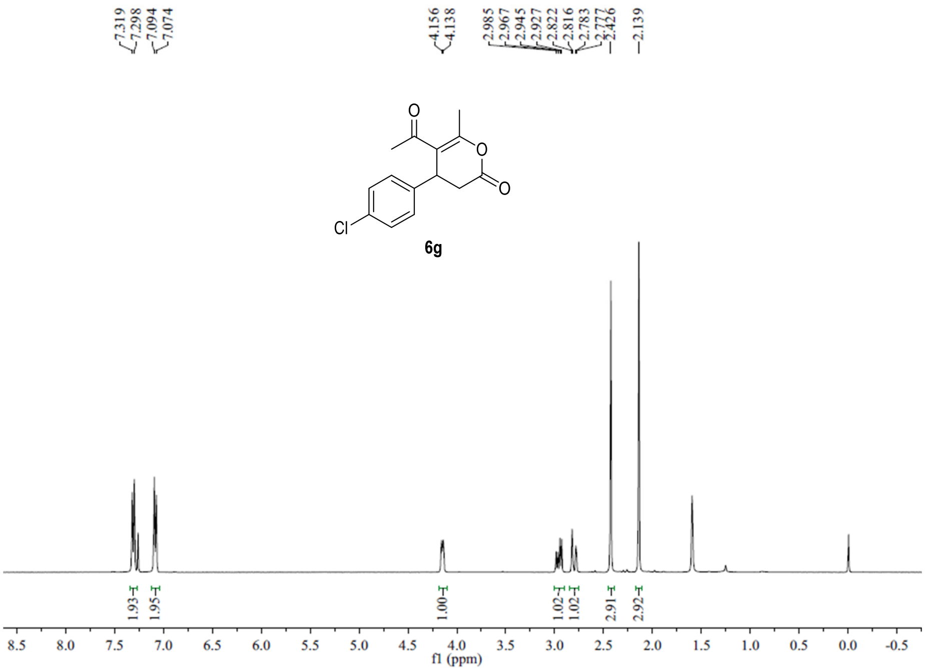

100 MHz, 298 K, in CDCl$_3$

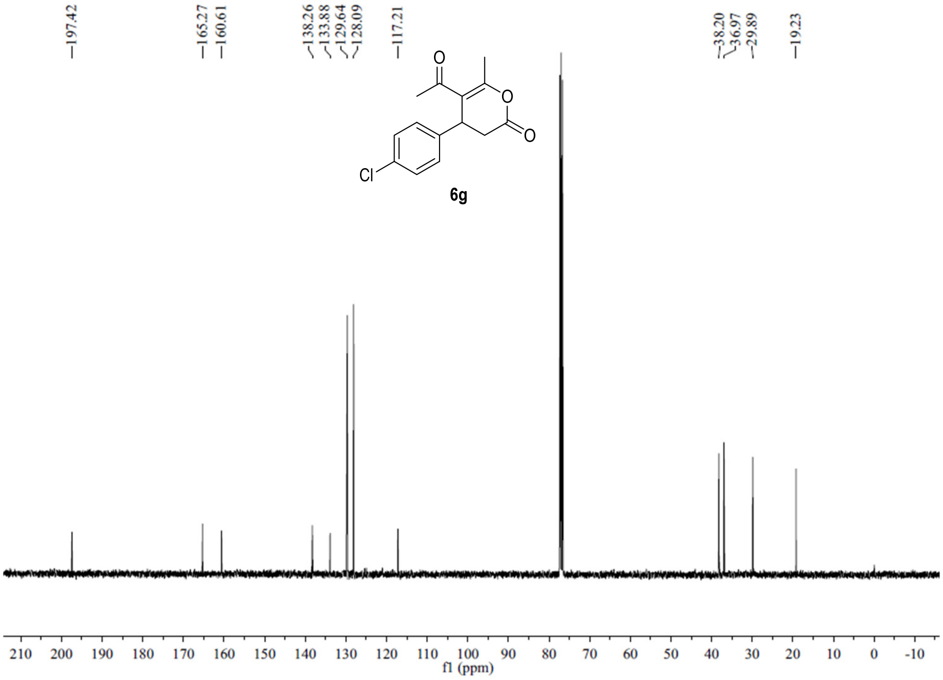

Supplementary Figure 29. ^{1}H and ^{13}C NMR spectra for compound **6g**.

400 MHz, 298 K, in $CDCl_3$

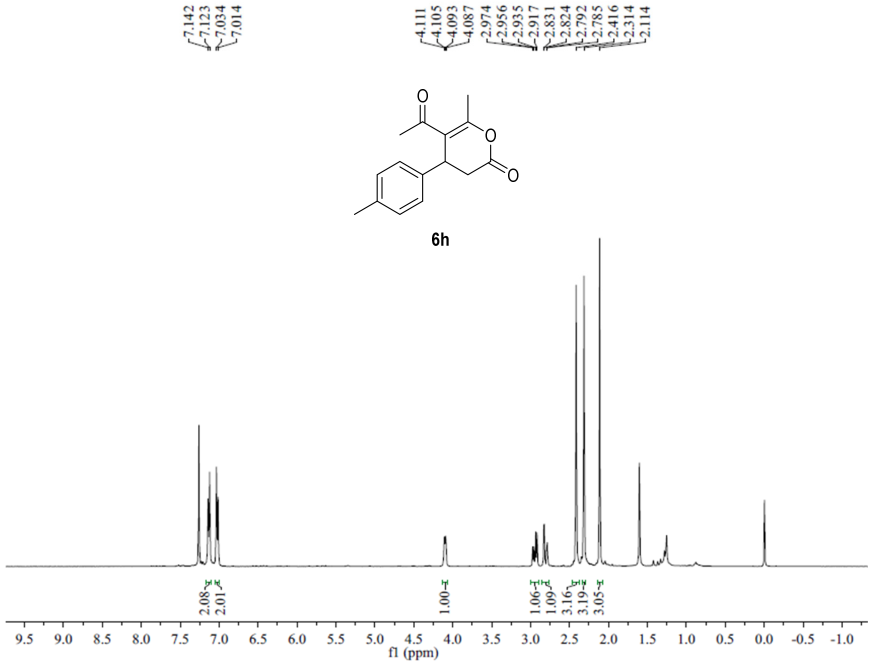

100 MHz, 298 K, in $CDCl_3$

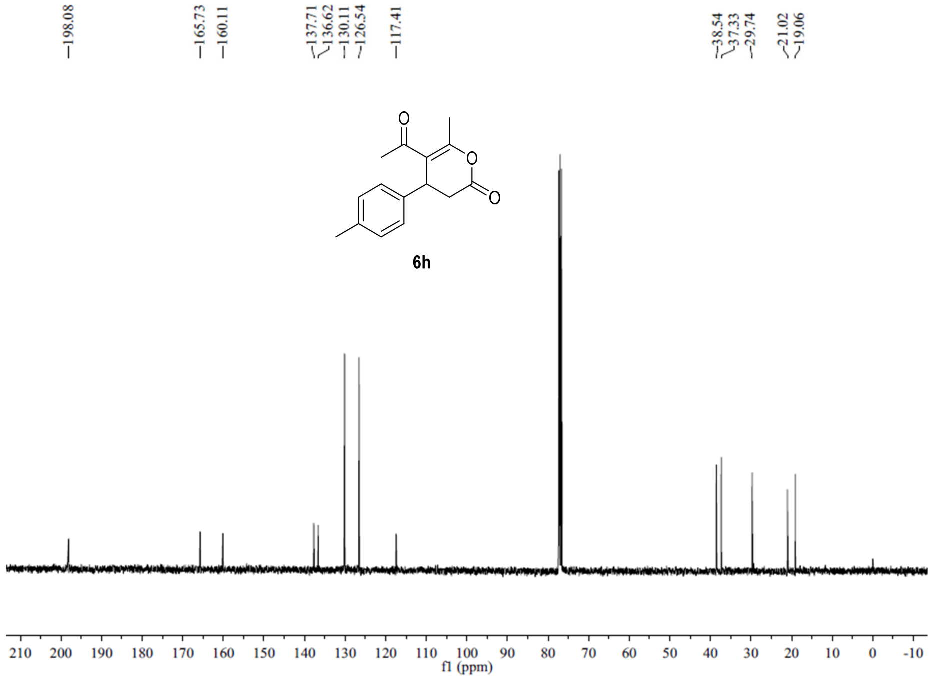

Supplementary Figure 30. ^{1}H and ^{13}C NMR spectra for compound **6h**.

400 MHz, 298 K, in CDCl$_3$

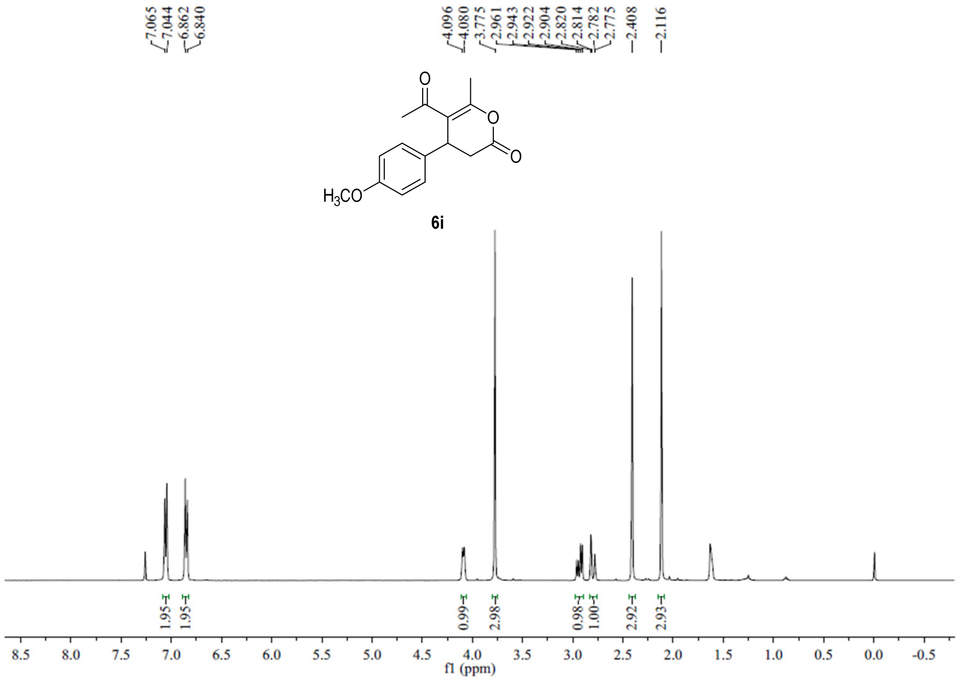

100 MHz, 298 K, in CDCl$_3$

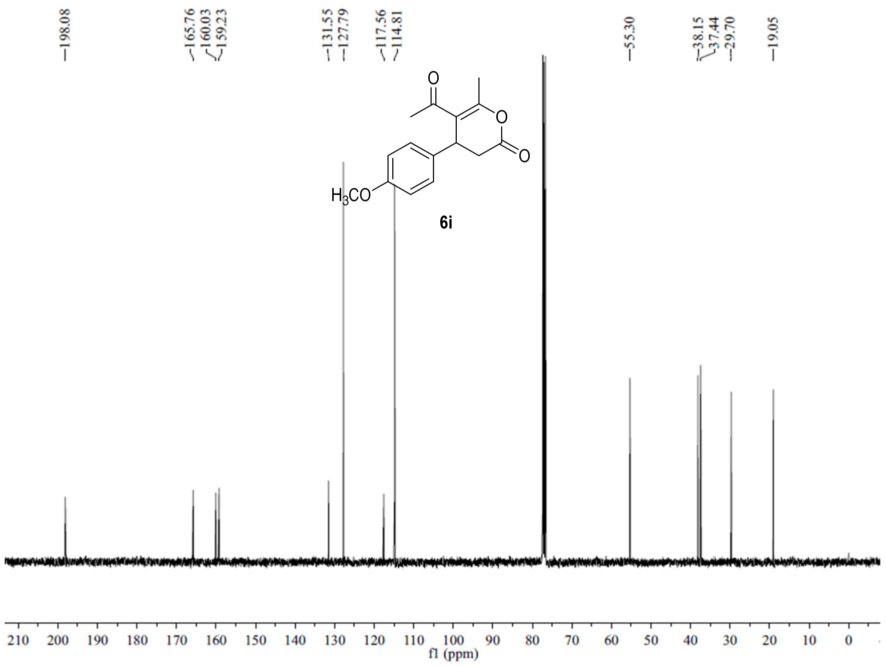

Supplementary Figure 31. ^{1}H and ^{13}C NMR spectra for compound **6i.**

400 MHz, 298 K, in $CDCl_3$

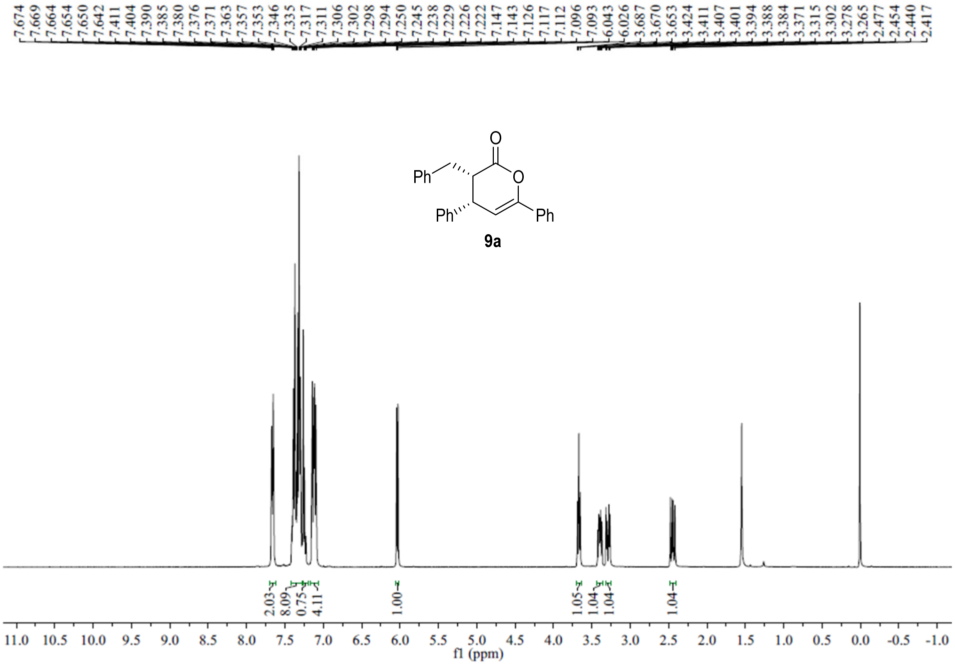

100 MHz, 298 K, in $CDCl_3$

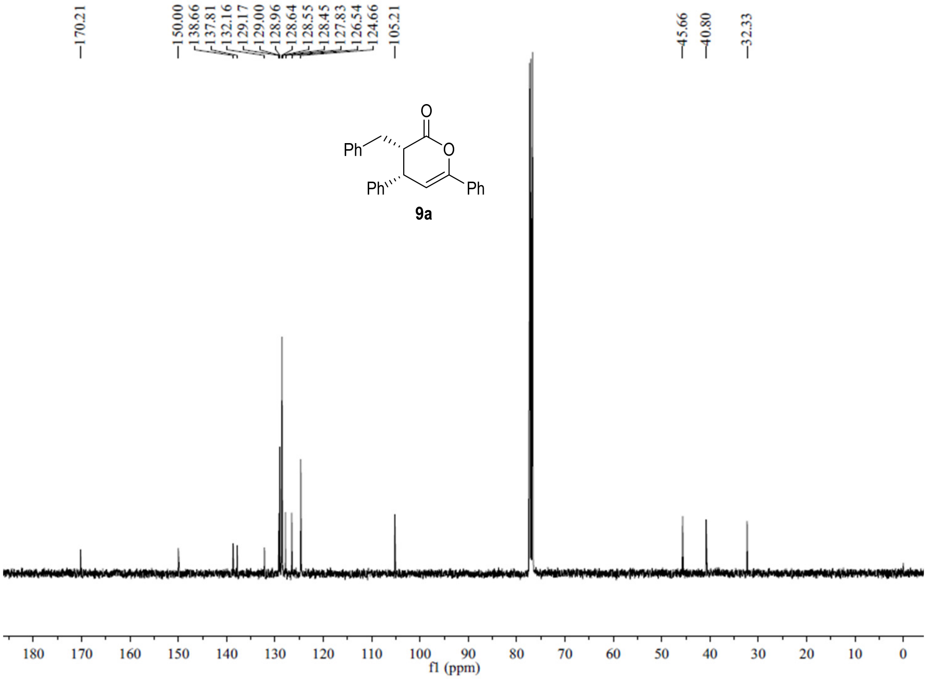

Supplementary Figure 32. ^{1}H and ^{13}C NMR spectra for compound **9a**.

400 MHz, 298 K, in $CDCl_3$

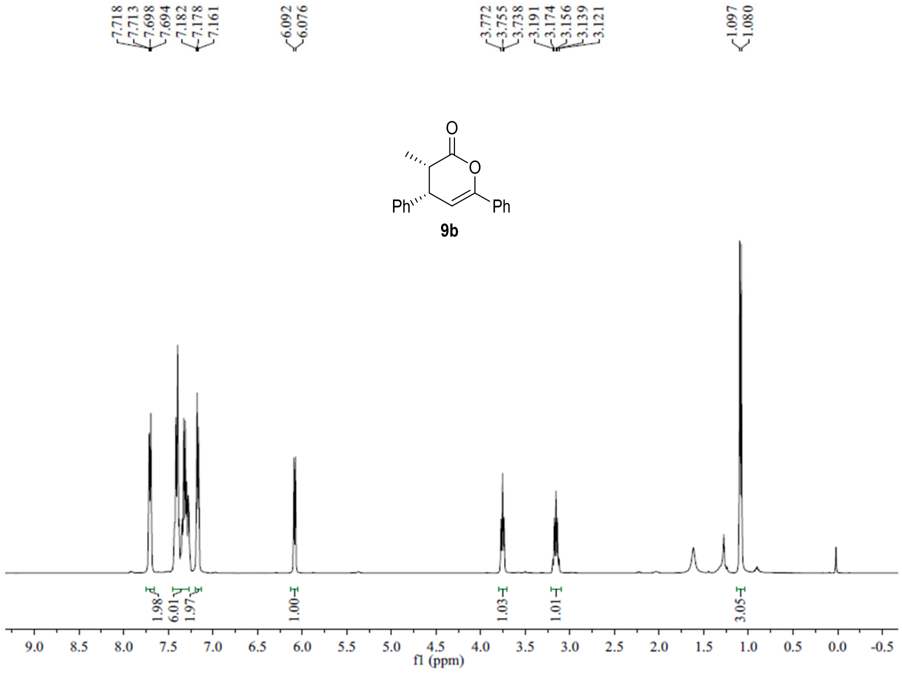

100 MHz, 298 K, in $CDCl_3$

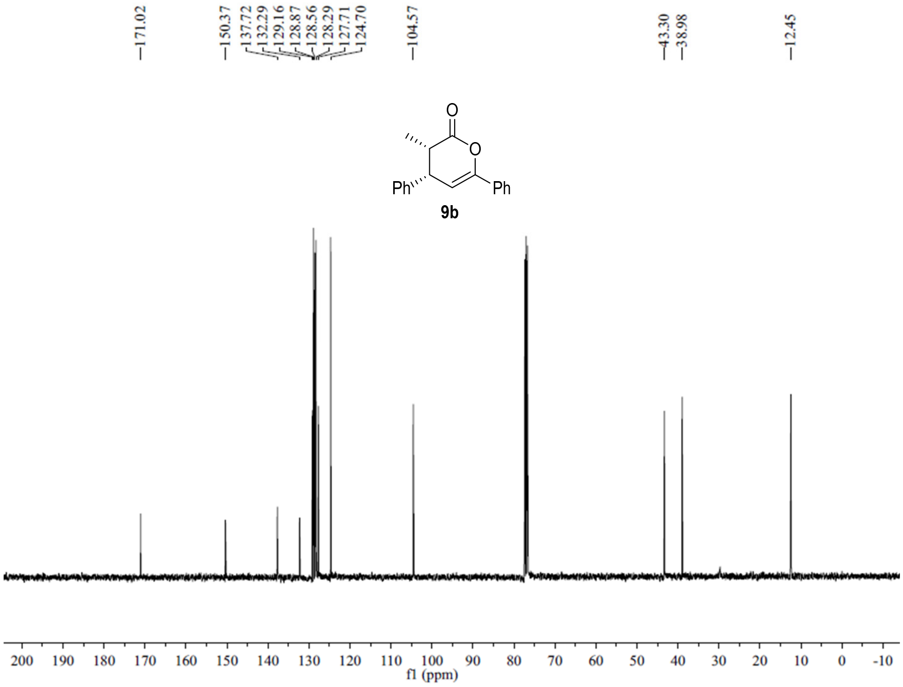

Supplementary Figure 33. ^{1}H and ^{13}C NMR spectra for compound **9b**.

400 MHz, 298 K, in $CDCl_3$

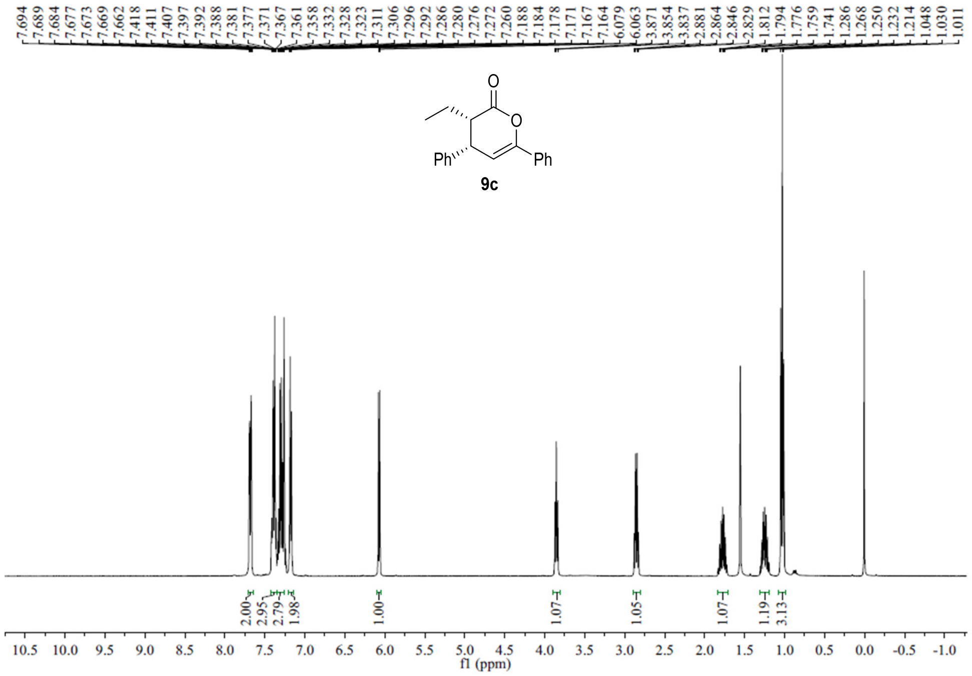

100 MHz, 298 K, in $CDCl_3$

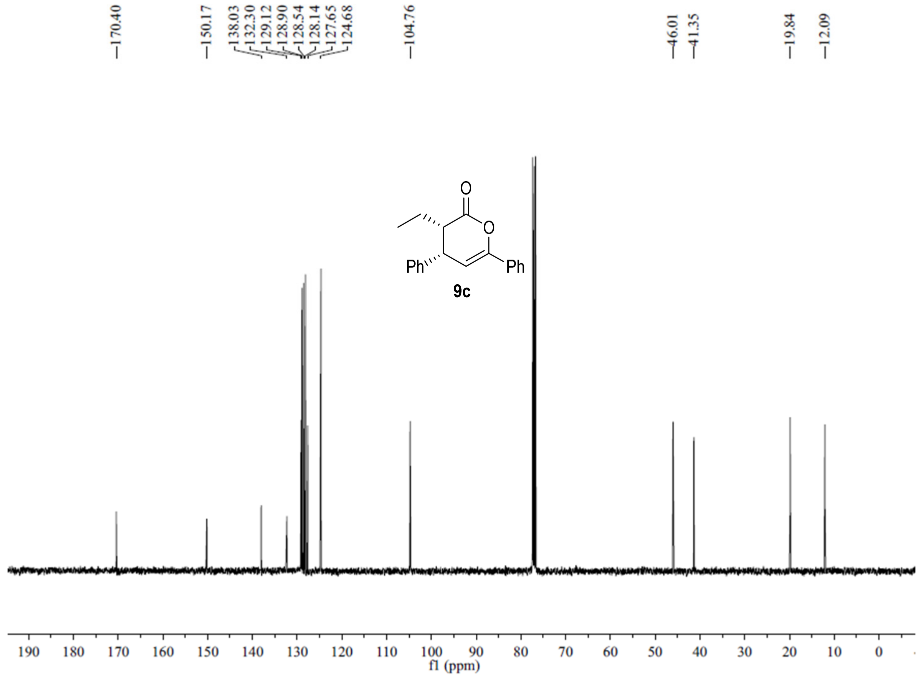

Supplementary Figure 34. ^{1}H and ^{13}C NMR spectra for compound **9c**.

400 MHz, 298 K, in CDCl$_3$

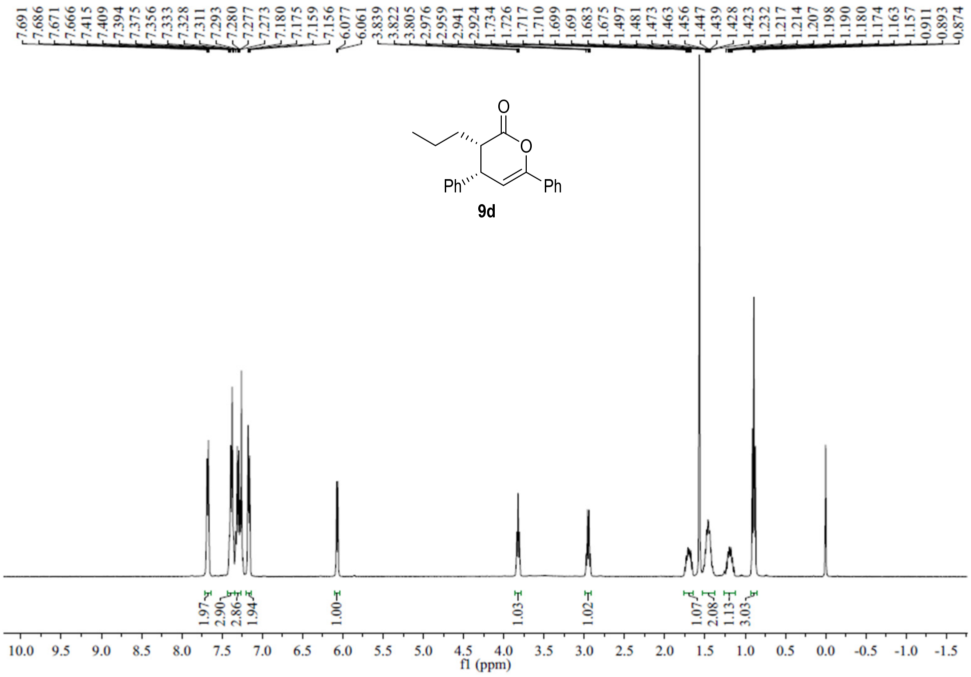

100 MHz, 298 K, in CDCl$_3$

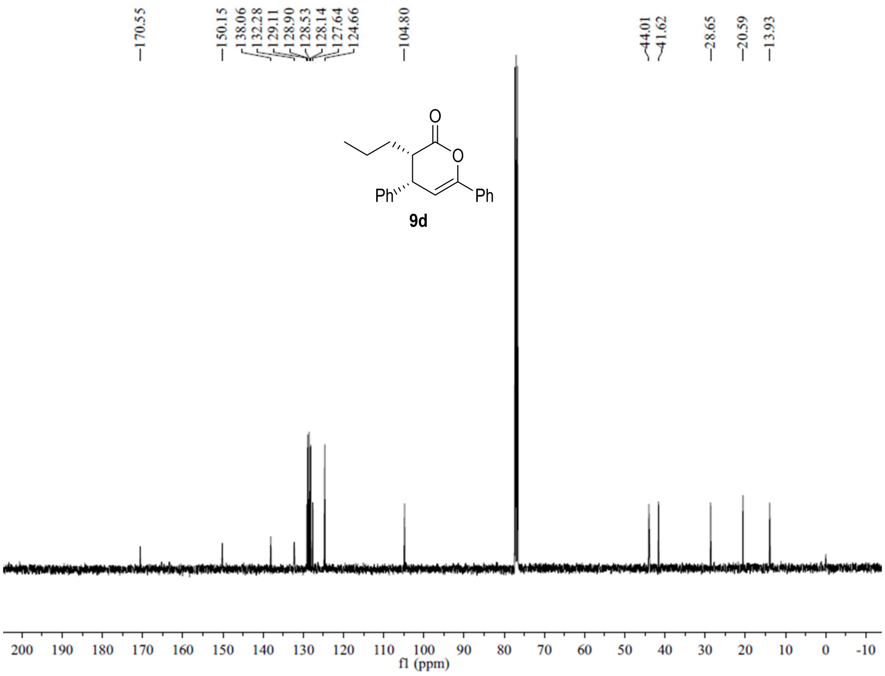

Supplementary Figure 35. ^{1}H and ^{13}C NMR spectra for compound **9d**.

400 MHz, 298 K, in $CDCl_3$

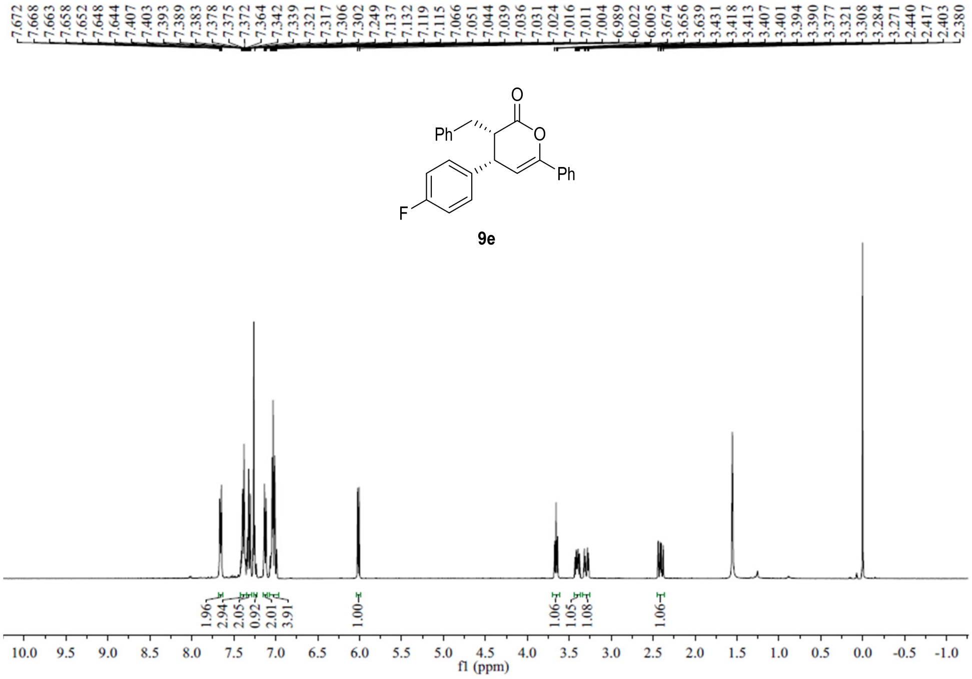

100 MHz, 298 K, in $CDCl_3$

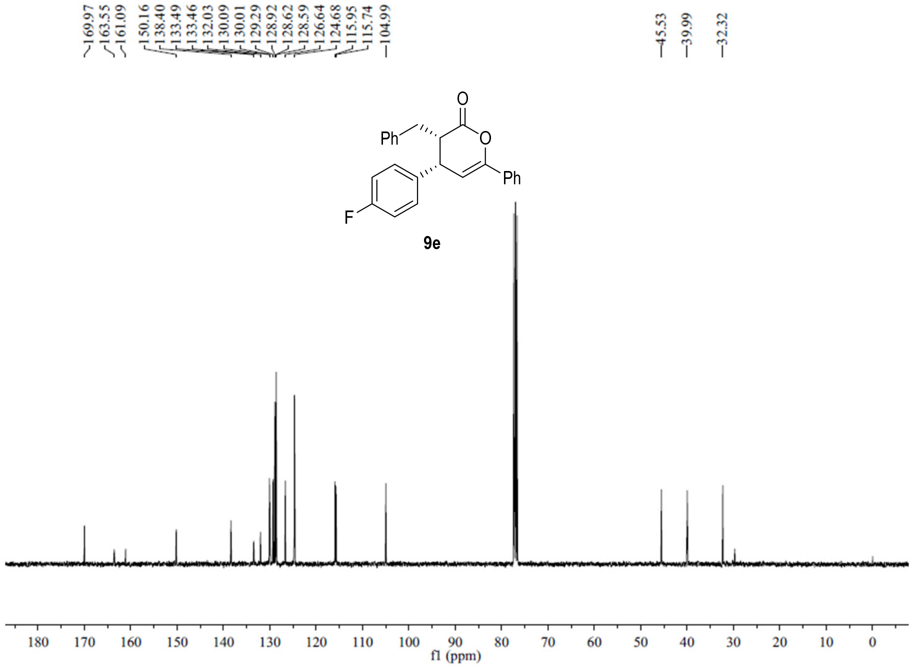

Supplementary Figure 36. ^{1}H and ^{13}C NMR spectra for compound **9e**.

400 MHz, 298 K, in $CDCl_3$

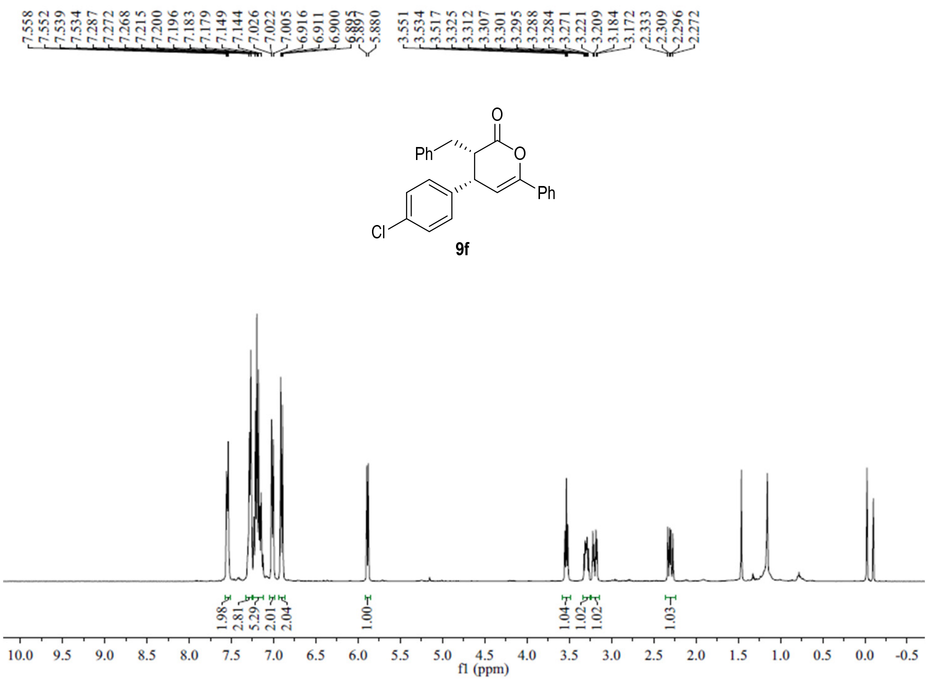

100 MHz, 298 K, in $CDCl_3$

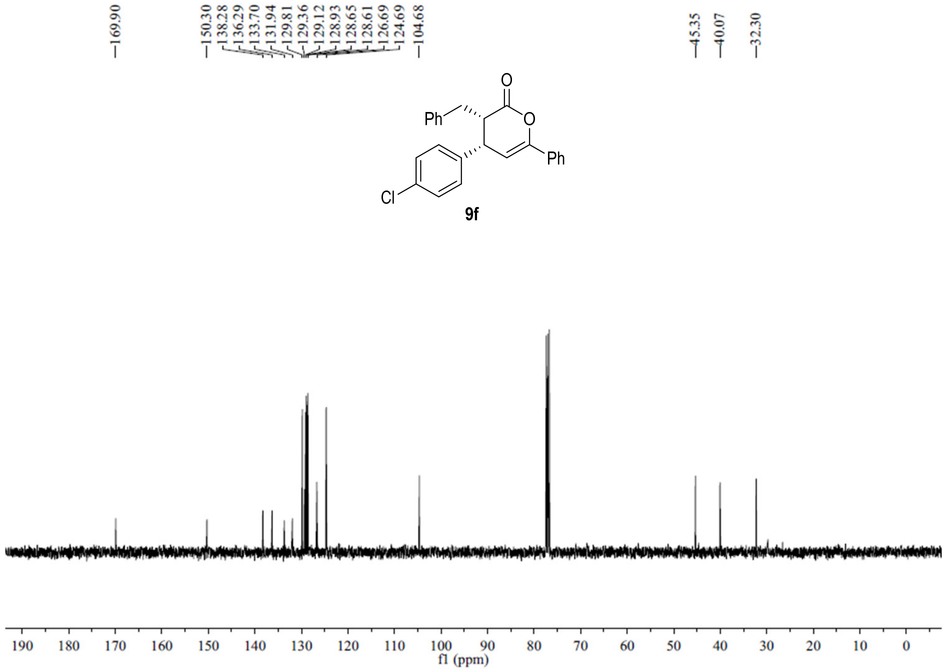

Supplementary Figure 37. ^{1}H and ^{13}C NMR spectra for compound **9f**.

400 MHz, 298 K, in $CDCl_3$

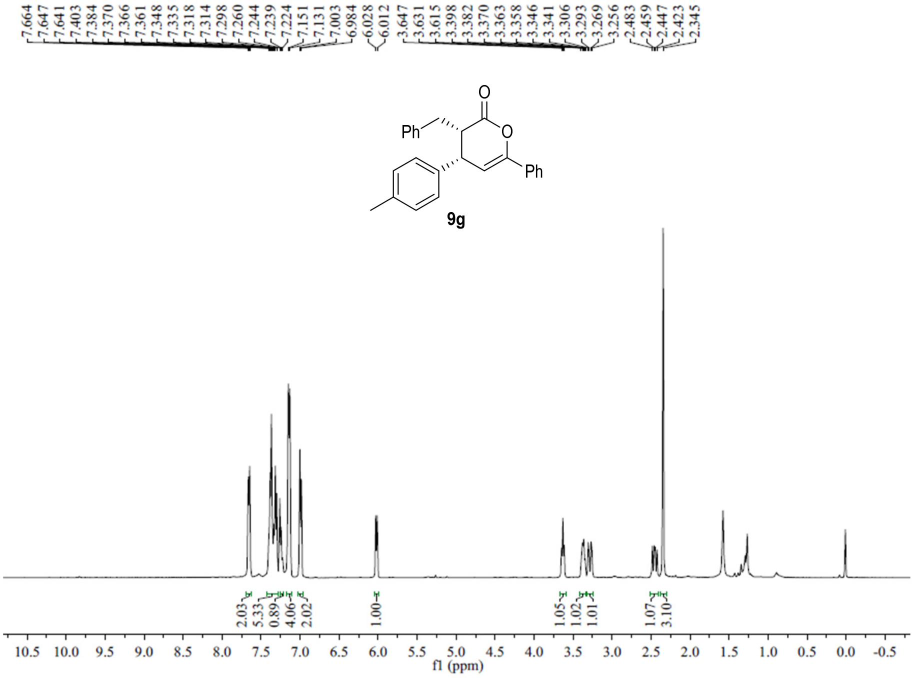

100 MHz, 298 K, in $CDCl_3$

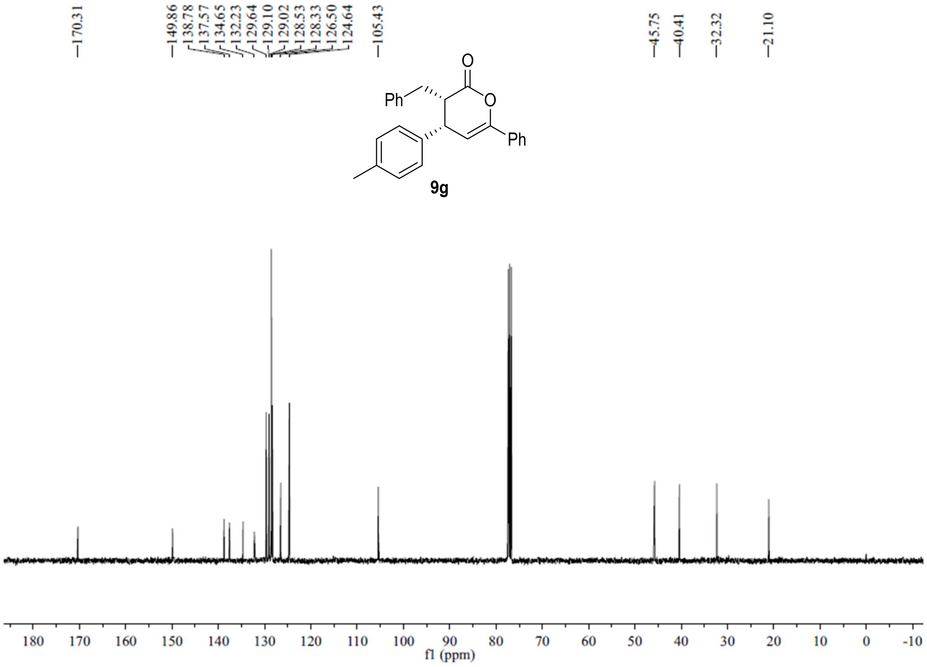

Supplementary Figure 38. ^{1}H and ^{13}C NMR spectra for compound **9g**.

400 MHz, 298 K, in $CDCl_3$

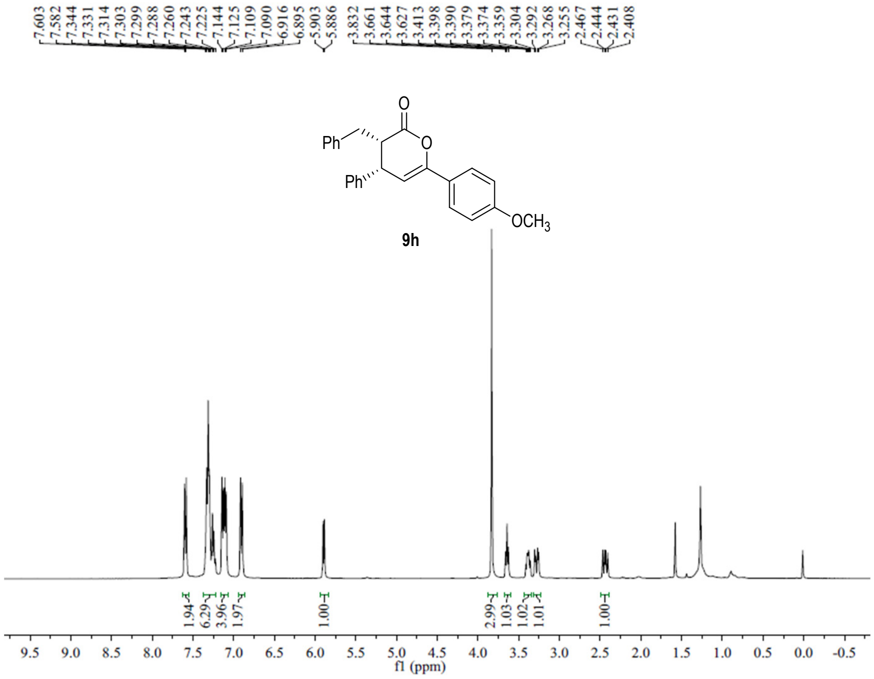

100 MHz, 298 K, in $CDCl_3$

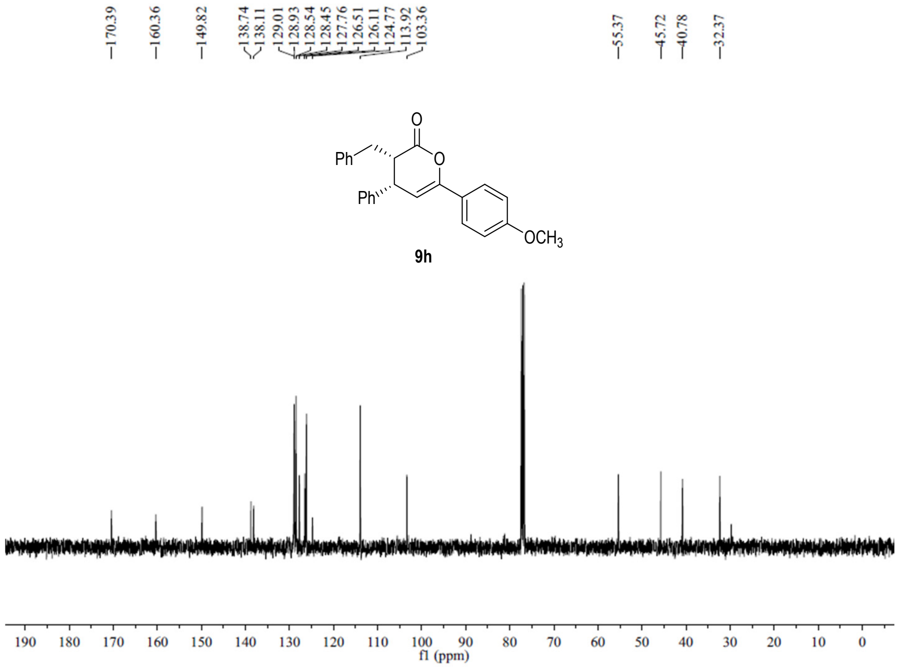

Supplementary Figure 39. ^{1}H and ^{13}C NMR spectra for compound **9h**.

400 MHz, 298 K, in $CDCl_3$

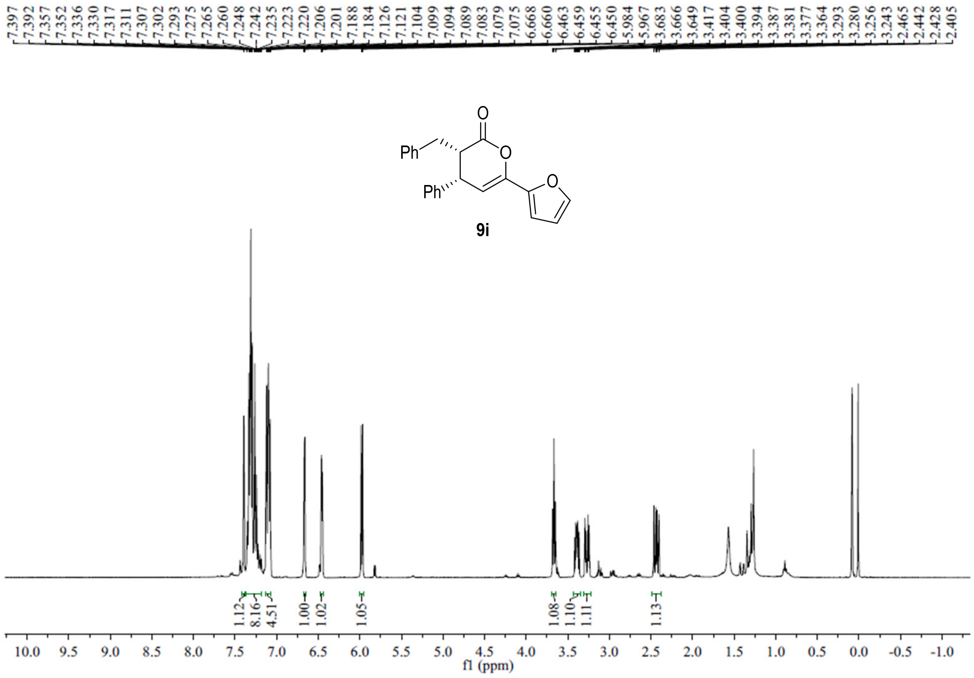

100 MHz, 298 K, in $CDCl_3$

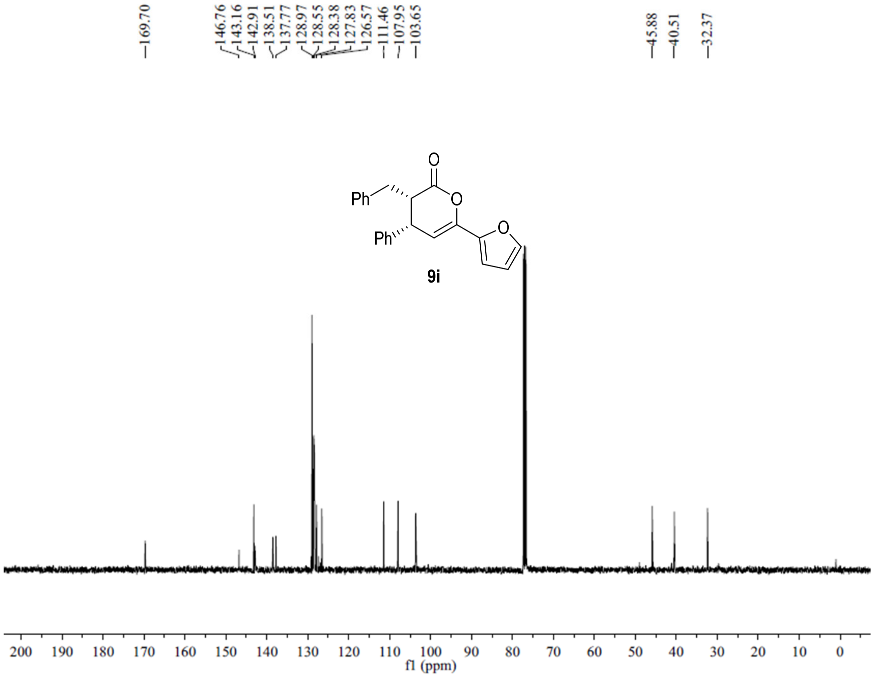

Supplementary Figure 40. ^{1}H and ^{13}C NMR spectra for compound **9i**.

400 MHz, 298 K, in $CDCl_3$

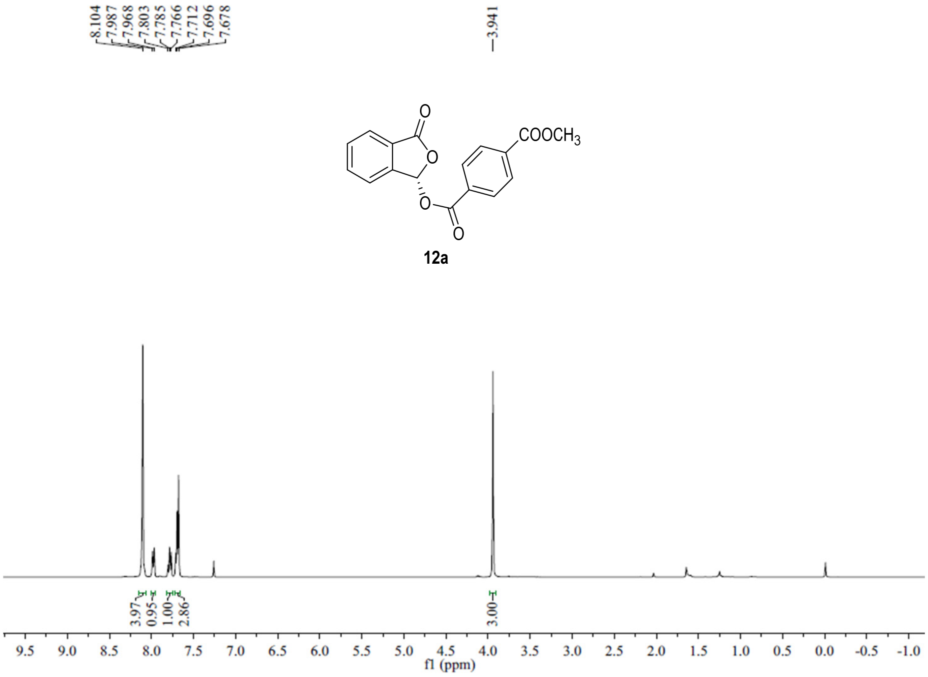

100 MHz, 298 K, in $CDCl_3$

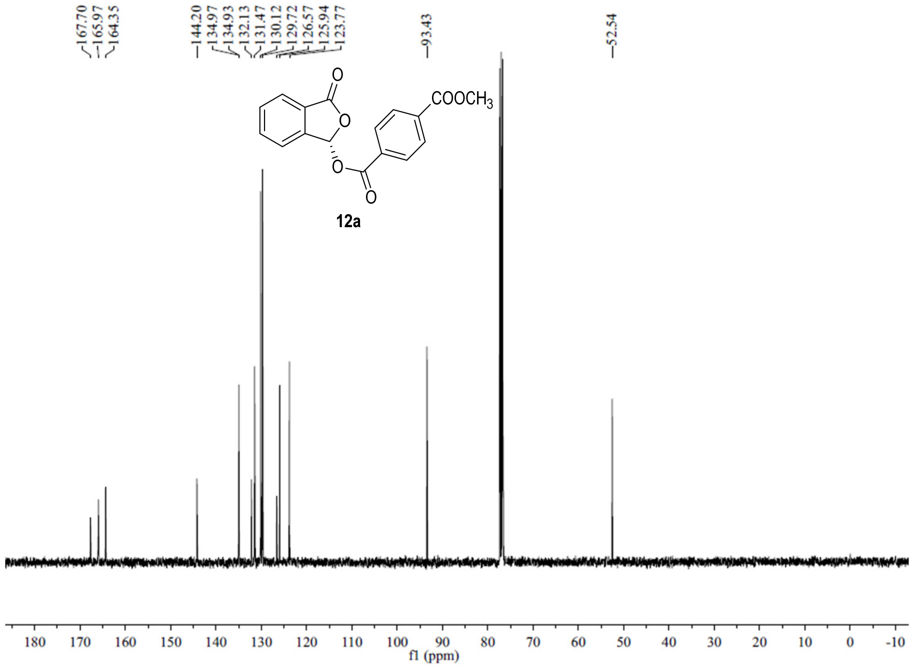

Supplementary Figure 41. ^{1}H and ^{13}C NMR spectra for compound **12a**.

400 MHz, 298 K, in $CDCl_3$

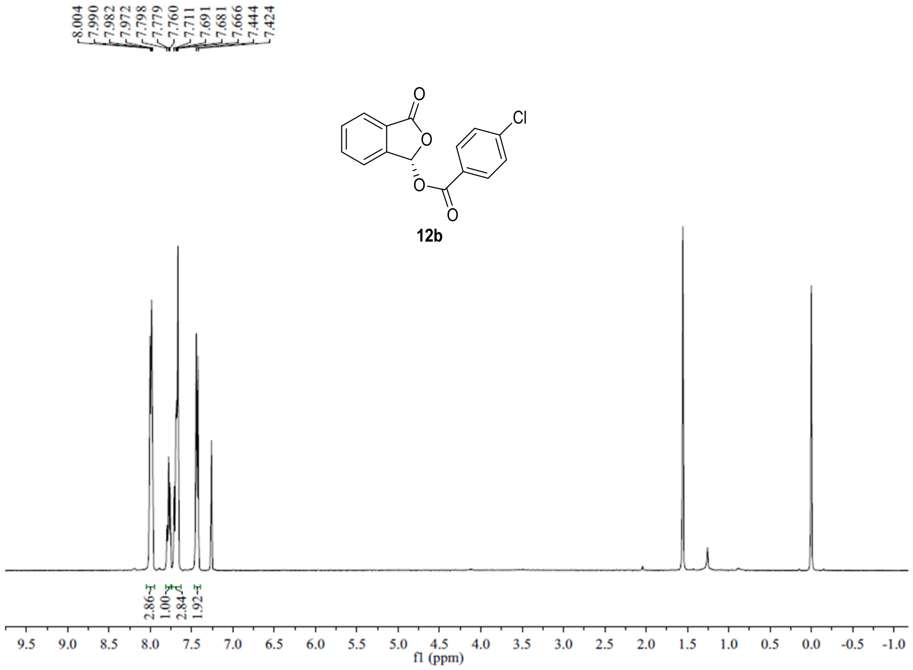

100 MHz, 298 K, in $CDCl_3$

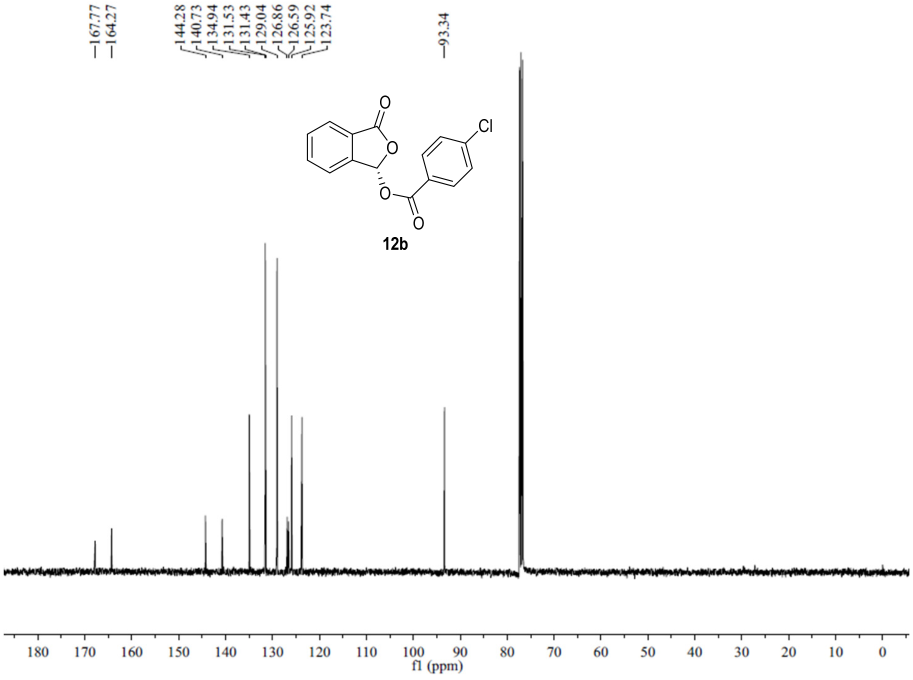

Supplementary Figure 42. ^{1}H and ^{13}C NMR spectra for compound **12b**.

400 MHz, 298 K, in $CDCl_3$

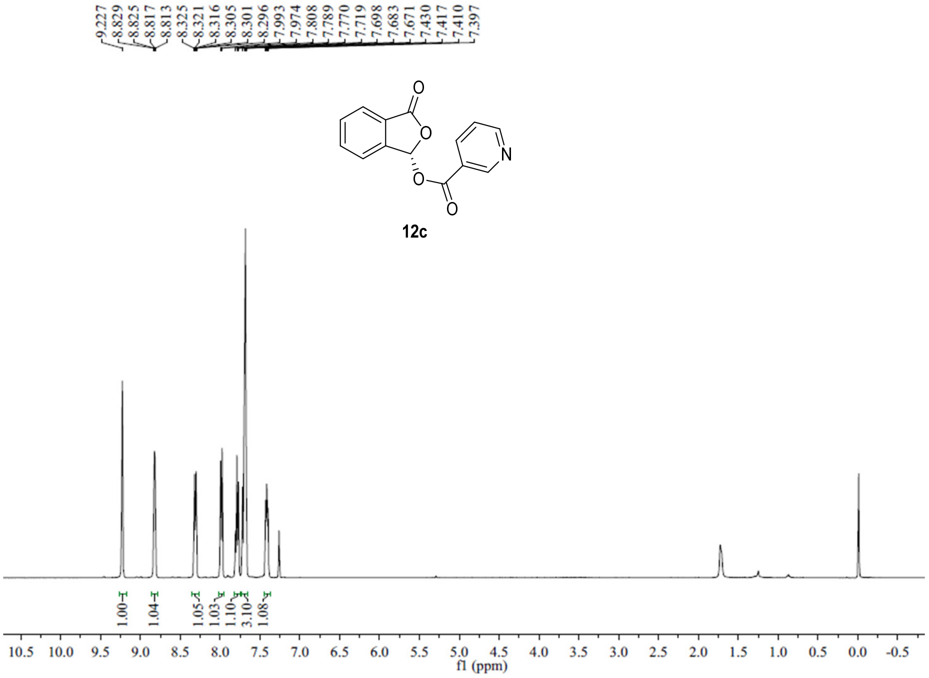

100 MHz, 298 K, in $CDCl_3$

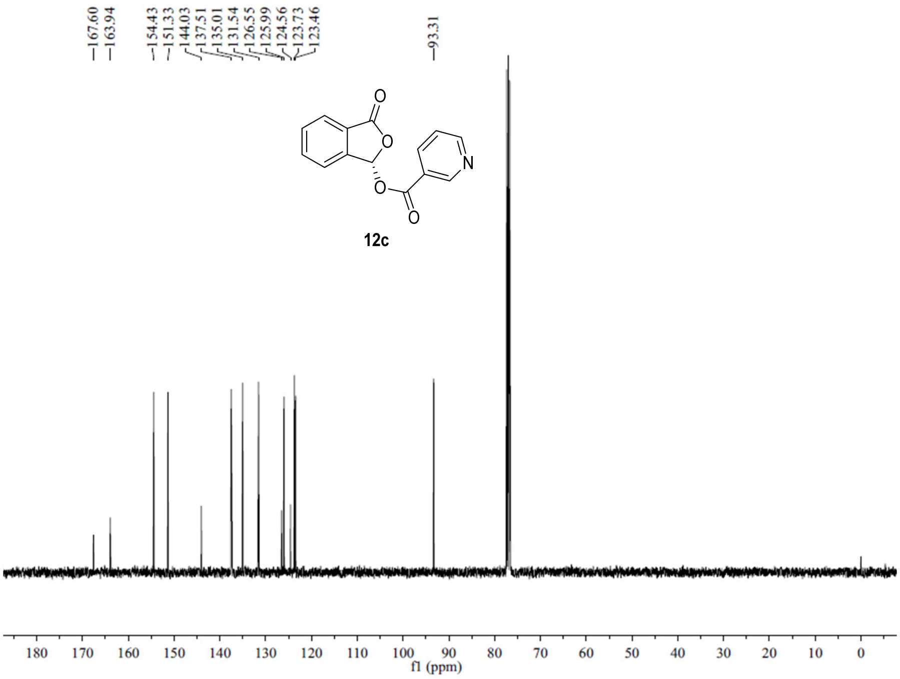

Supplementary Figure 43. ^{1}H and ^{13}C NMR spectra for compound **12c**.

400 MHz, 298 K, in $CDCl_3$

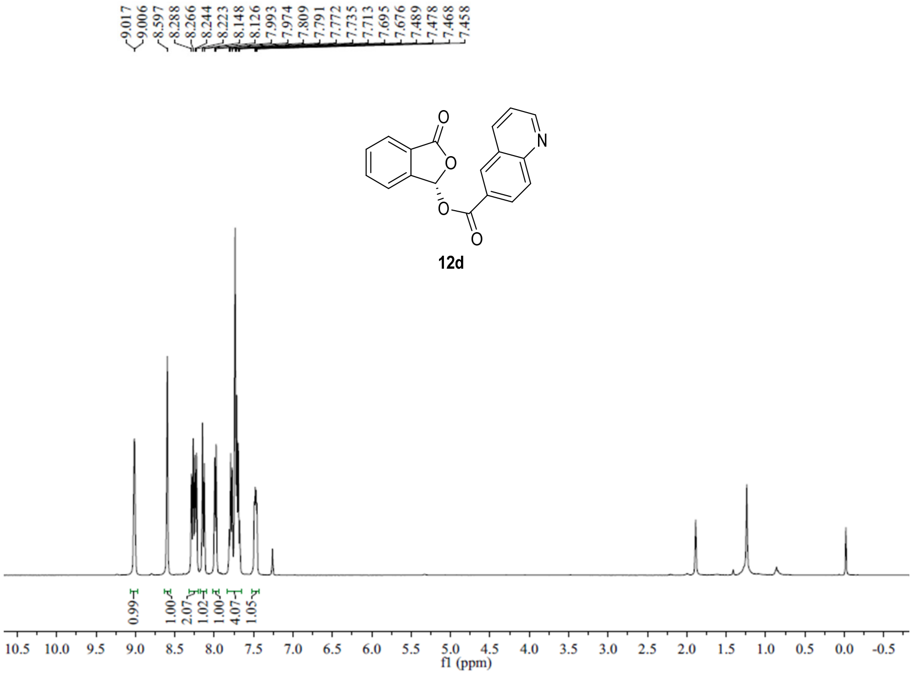

100 MHz, 298 K, in $CDCl_3$

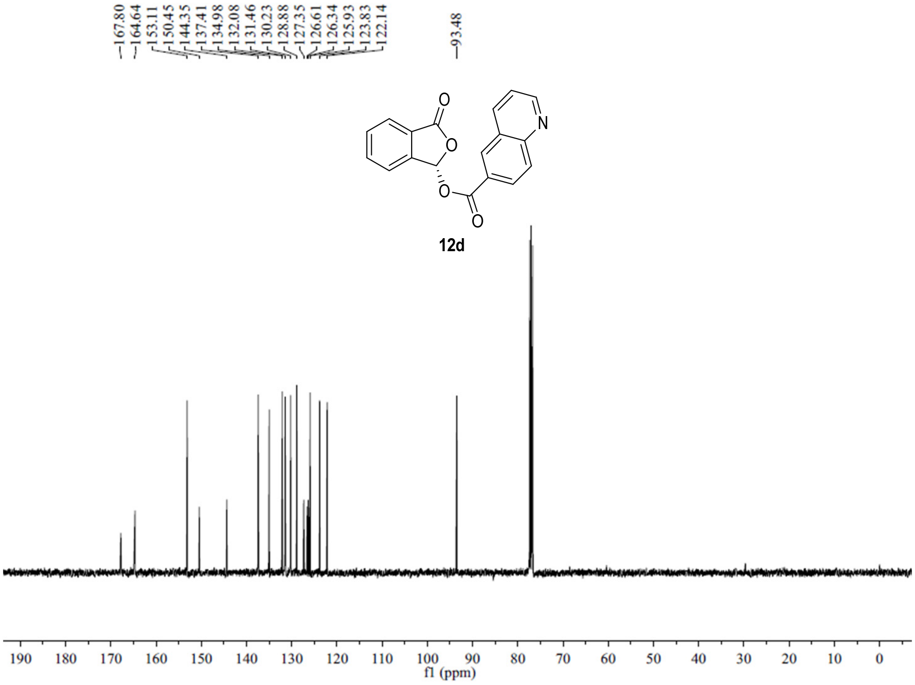

Supplementary Figure 44. ^{1}H and ^{13}C NMR spectra for compound **12d**.

400 MHz, 298 K, in $CDCl_3$

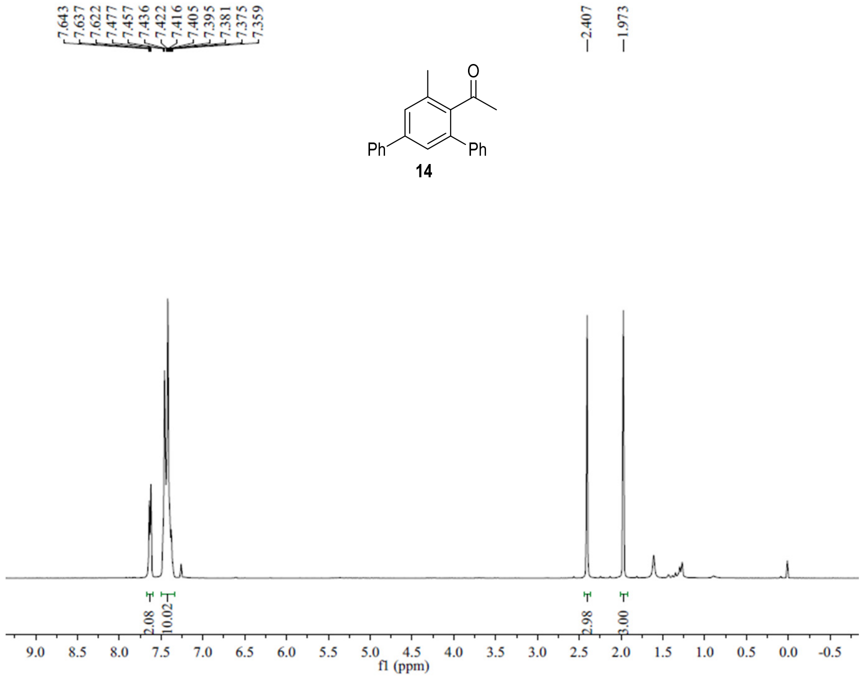

100 MHz, 298 K, in $CDCl_3$

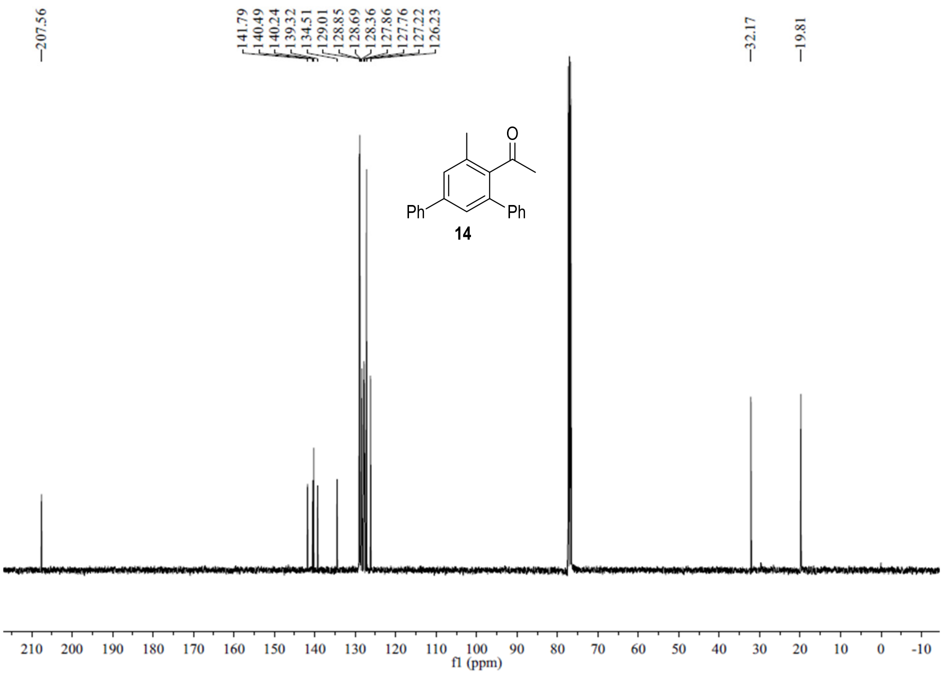

Supplementary Figure 45. ^{1}H and ^{13}C NMR spectra for compound **14**.

400 MHz, 298 K, in $CDCl_3$

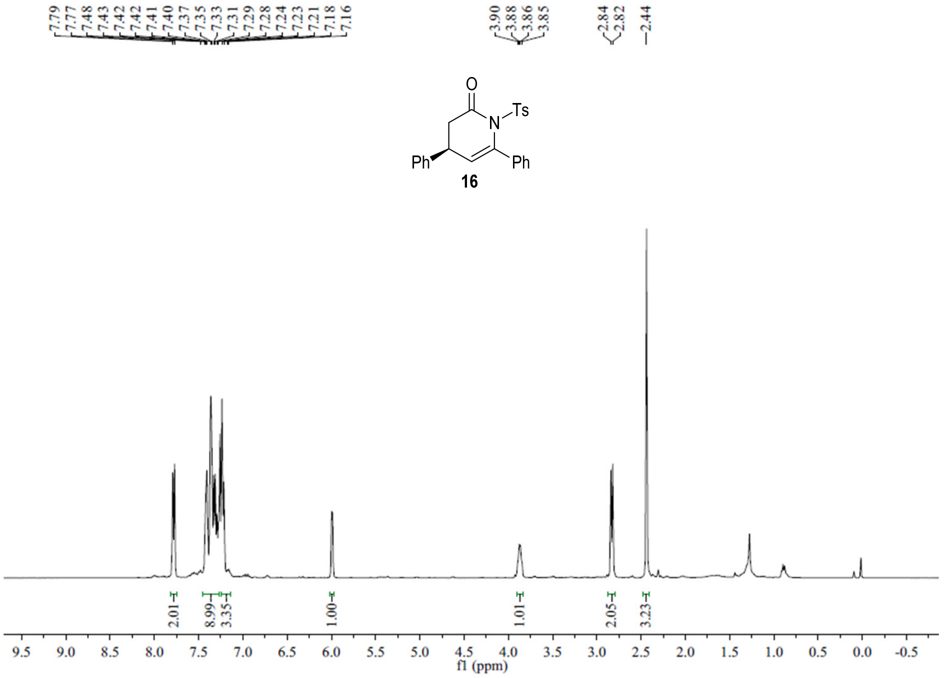

100 MHz, 298 K, in $CDCl_3$

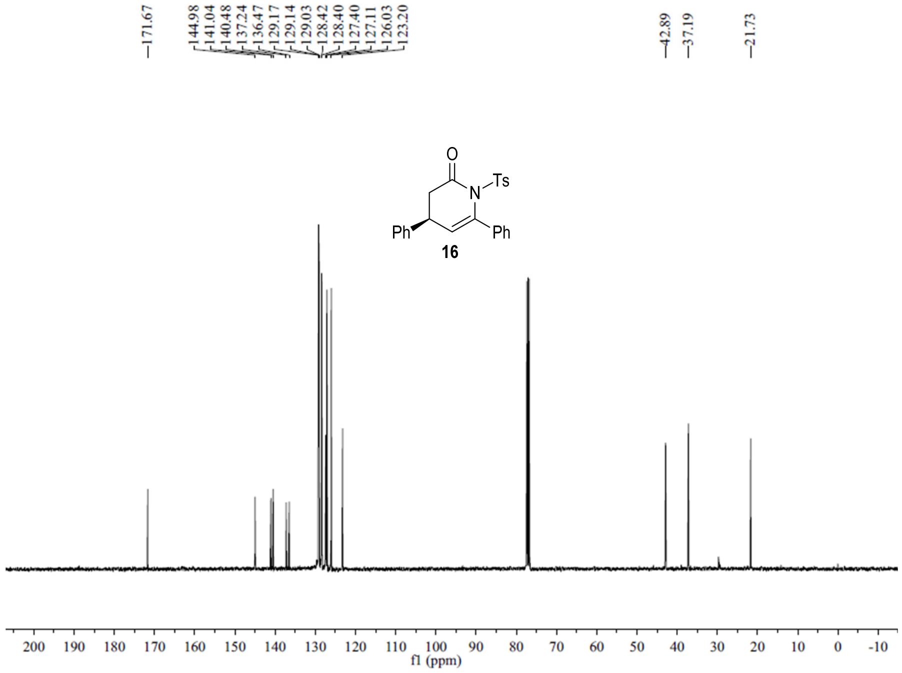

Supplementary Figure 46. ^{1}H and ^{13}C NMR spectra for compound **16**.

400 MHz, 298 K, in $CDCl_3$

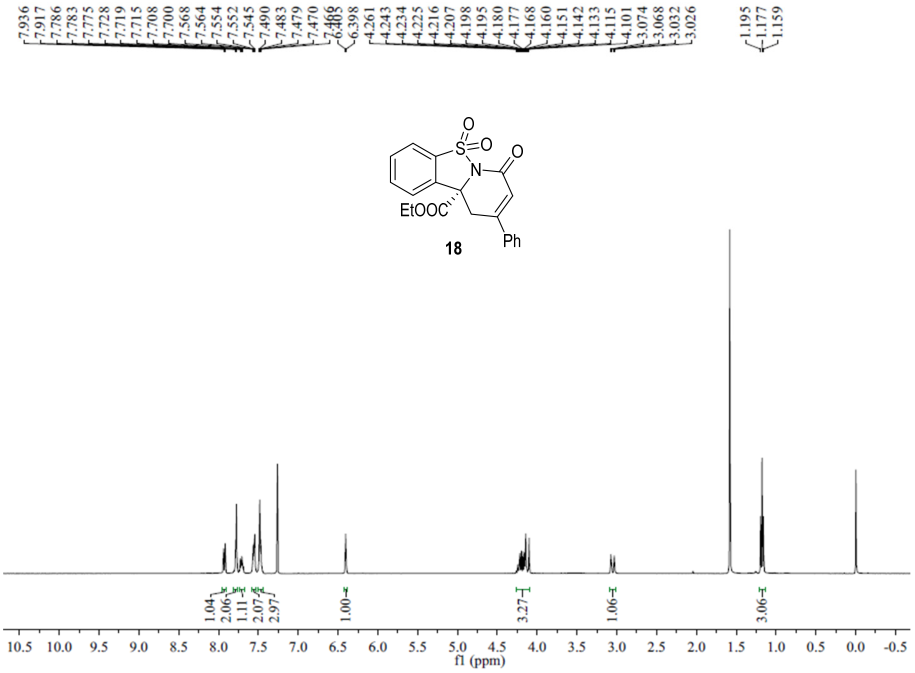

100 MHz, 298 K, in $CDCl_3$

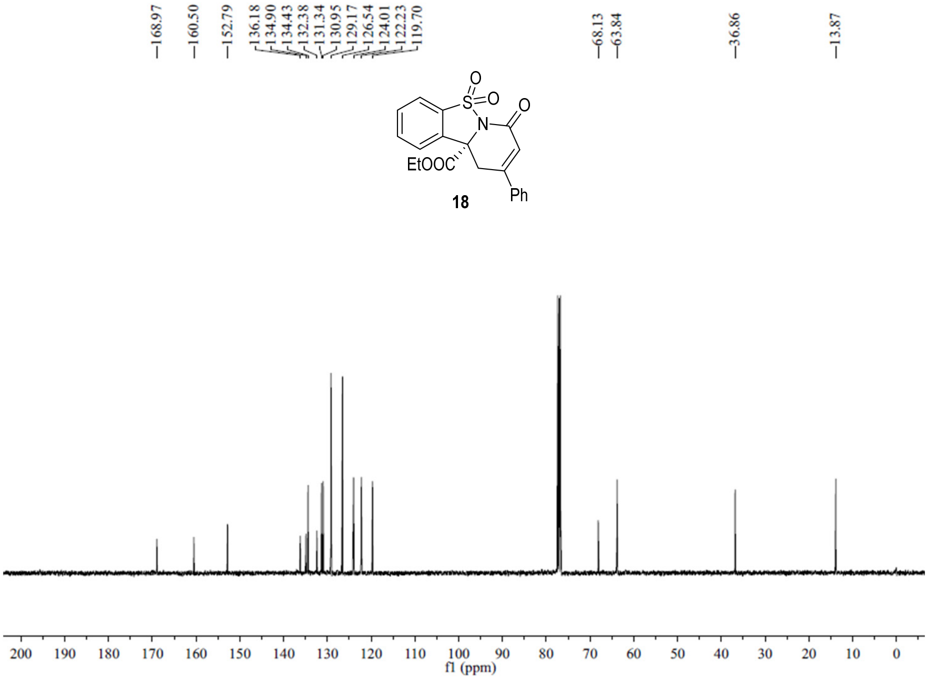

Supplementary Figure 47. ^{1}H and ^{13}C NMR spectra for compound **18**.

400 MHz, 298 K, in DMSO-d_6

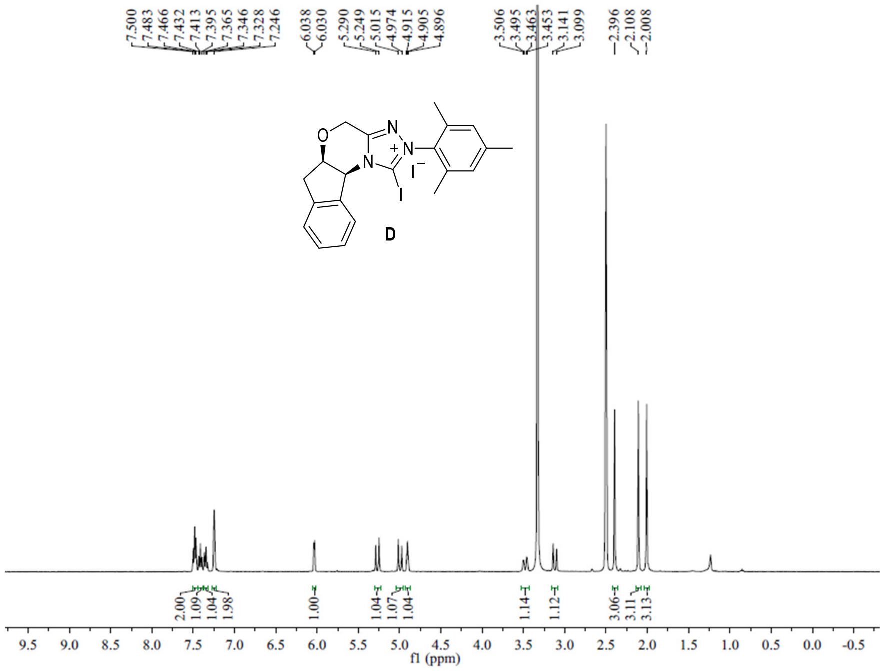

100 MHz, 298 K, in DMSO-d_6

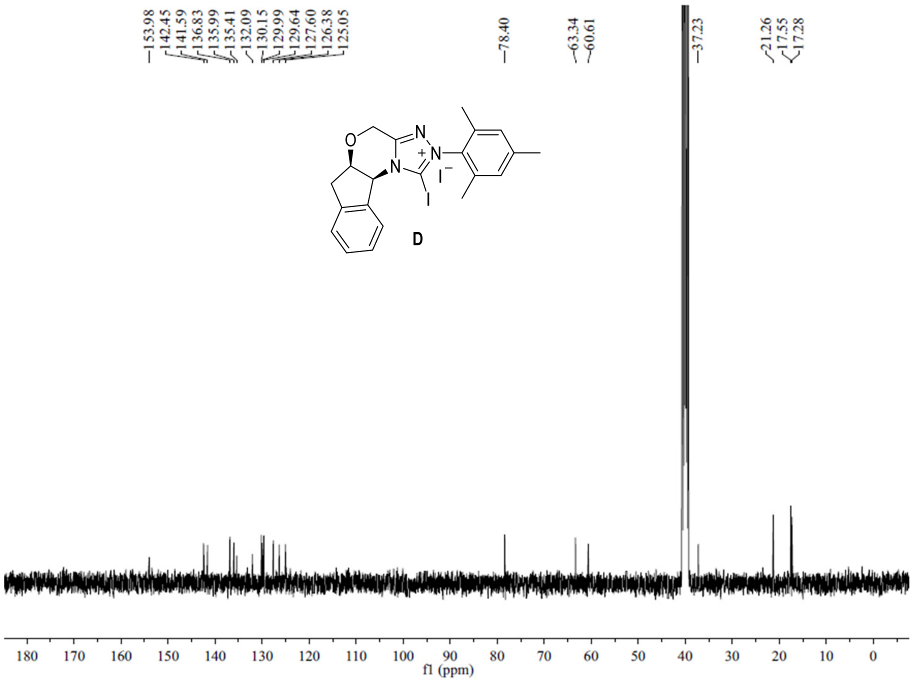

Supplementary Figure 48. ^{1}H and ^{13}C NMR spectra for compound **D**.

400 MHz, 298 K, in $CDCl_3$

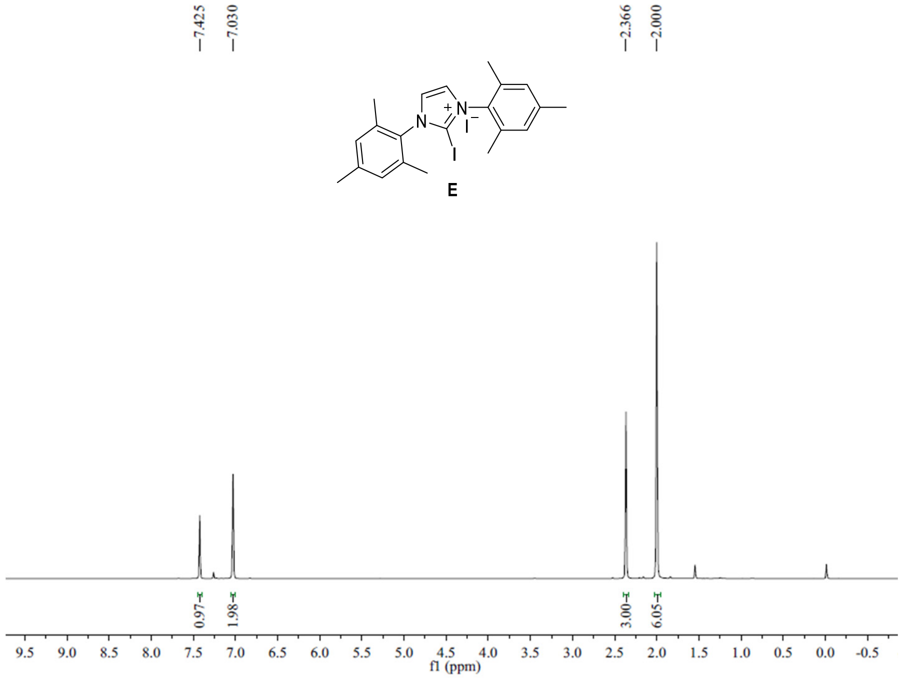

100 MHz, 298 K, in $CDCl_3$

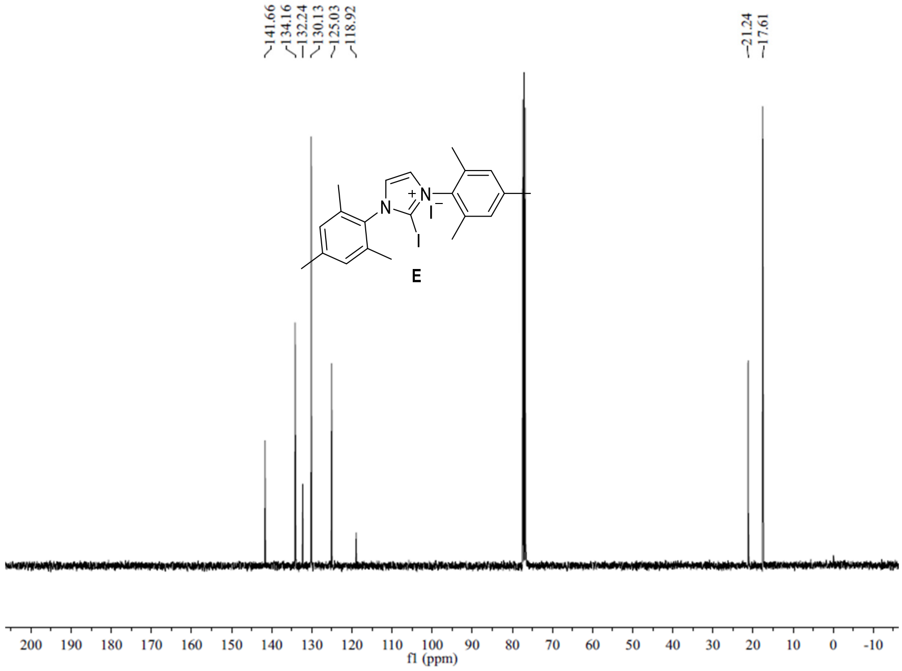

Supplementary Figure 49. ^{1}H and ^{13}C NMR spectra for compound **E**.

400 MHz, 298 K, in $CDCl_3$

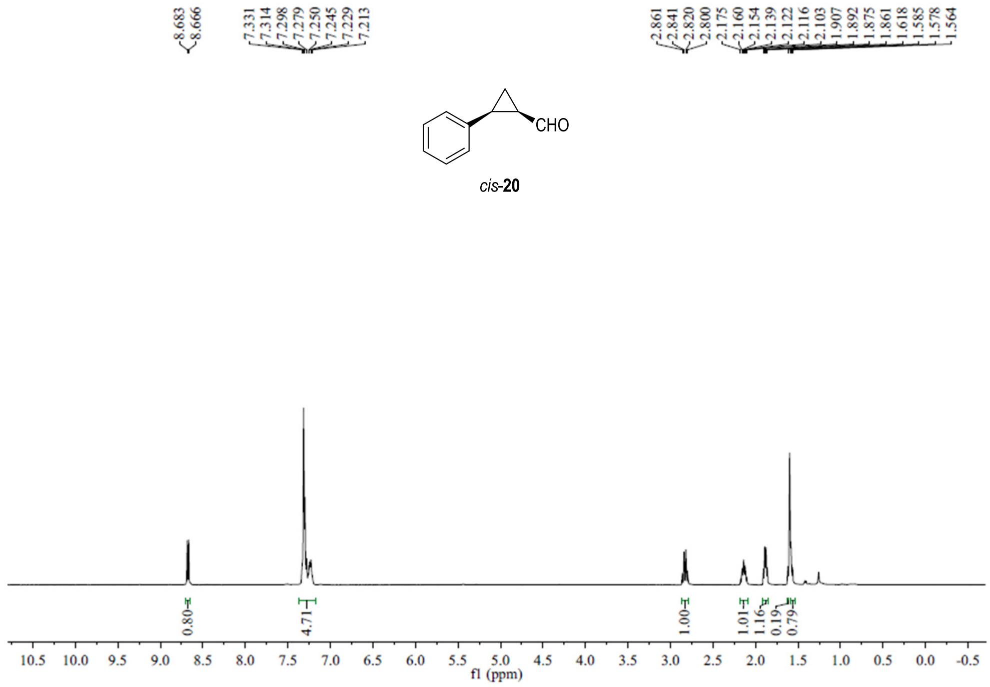

100 MHz, 298 K, in $CDCl_3$

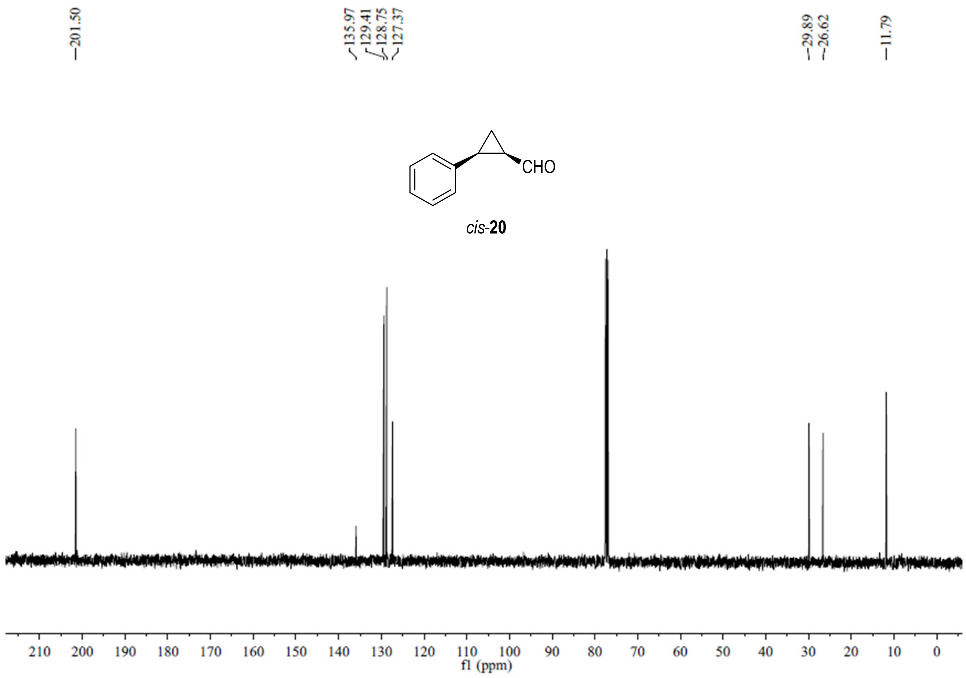

Supplementary Figure 50. ^{1}H and ^{13}C NMR spectra for compound *cis*-**20**.

400 MHz, 298 K, in $CDCl_3$

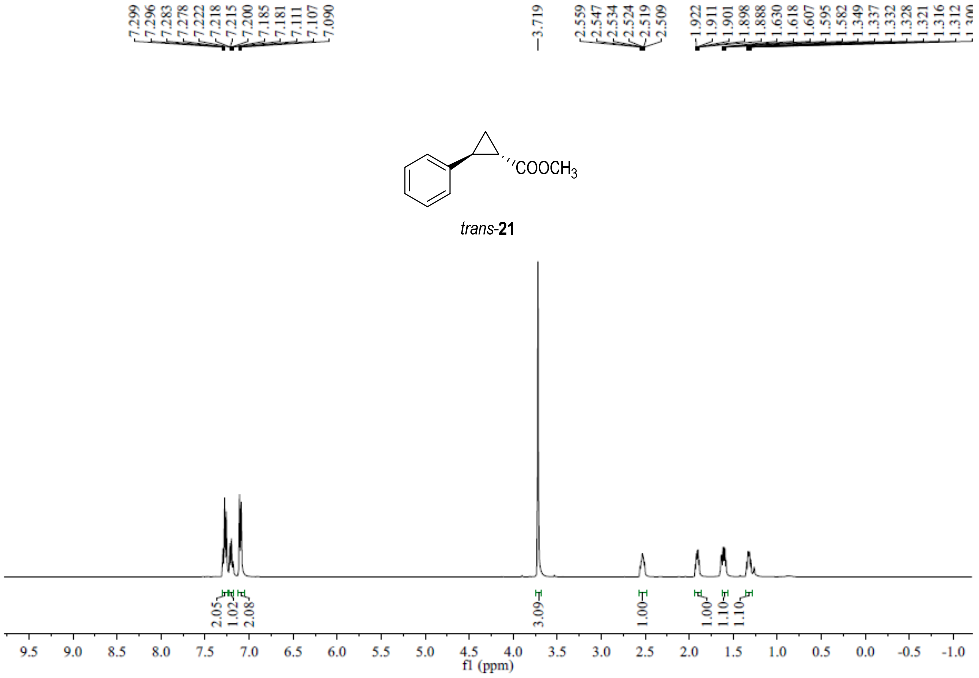

100 MHz, 298 K, in $CDCl_3$

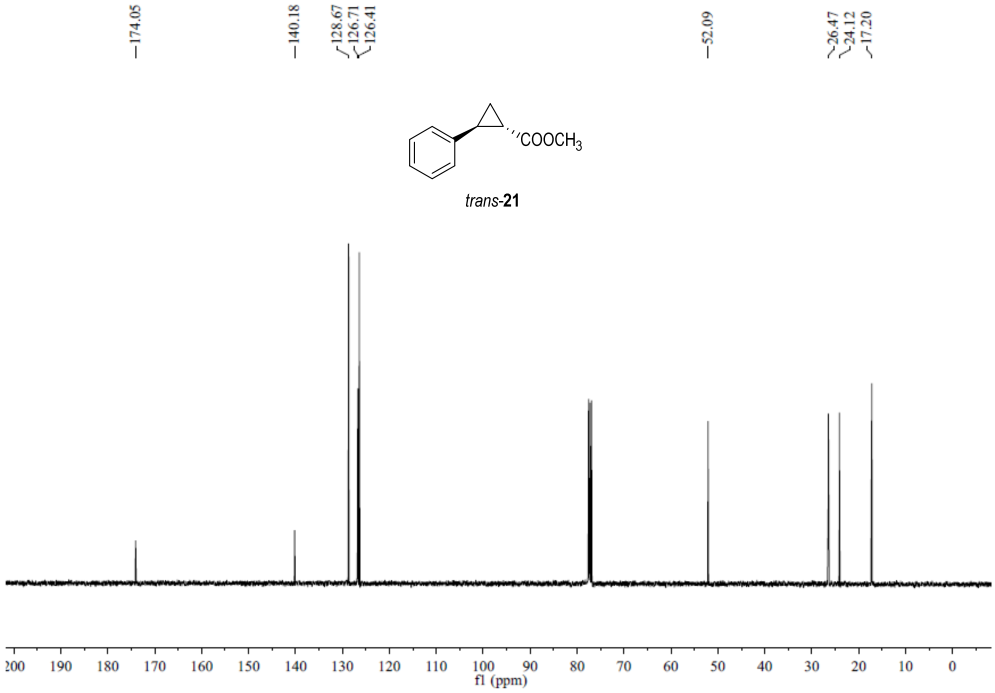

Supplementary Figure 51. ^{1}H and ^{13}C NMR spectra for compound *trans*-**21**.

400 MHz, 298 K, in $CDCl_3$

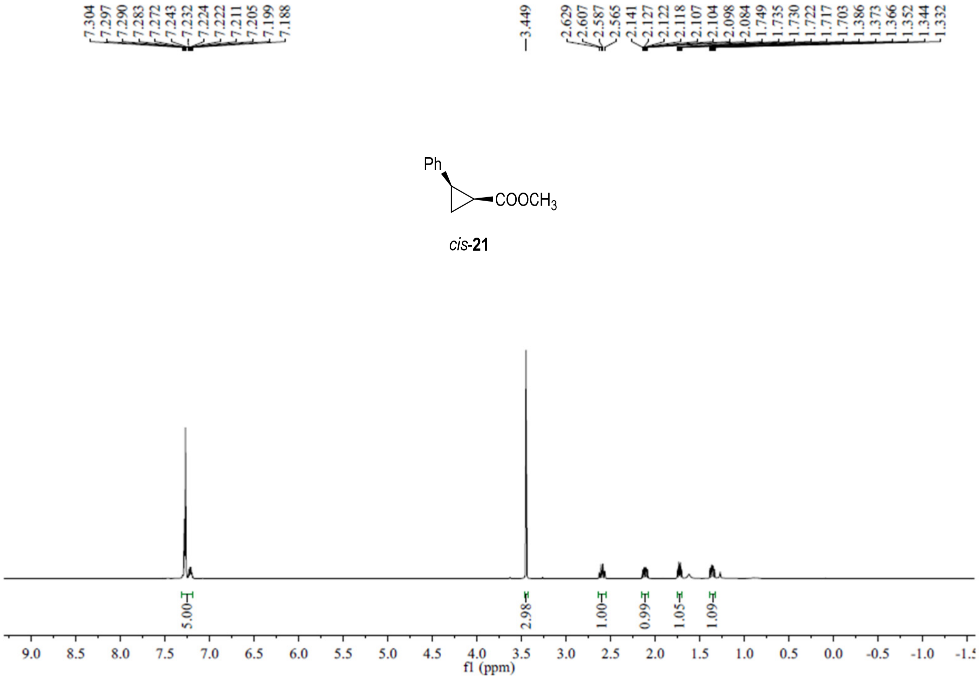

100 MHz, 298 K, in $CDCl_3$

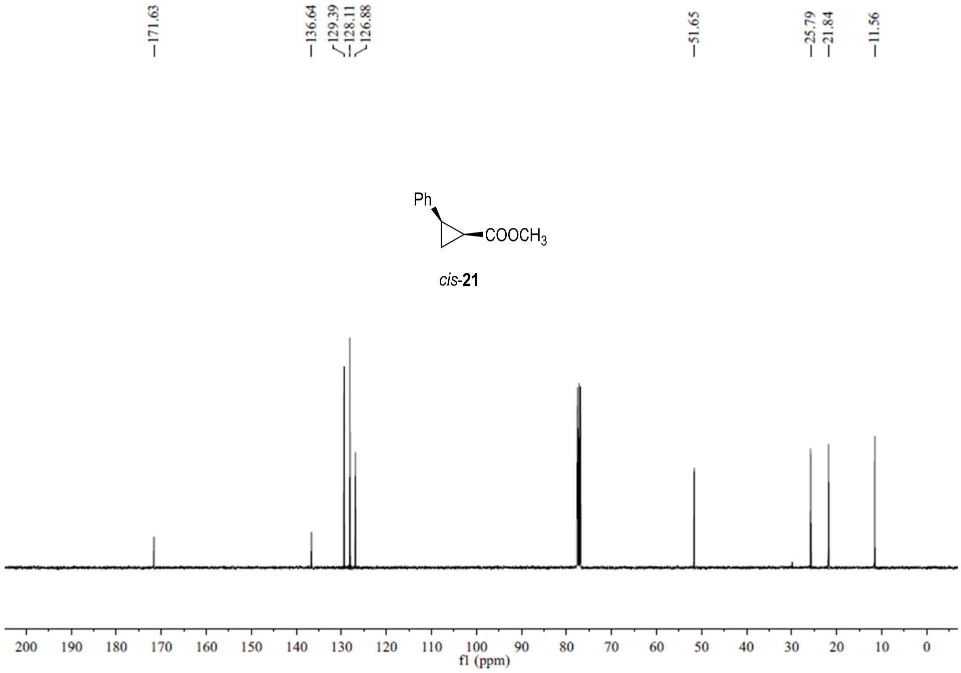

Supplementary Figure 52. ^{1}H and ^{13}C NMR spectra for compound *cis*-**21**.

3.3 UPCC Spectra

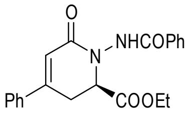

3a

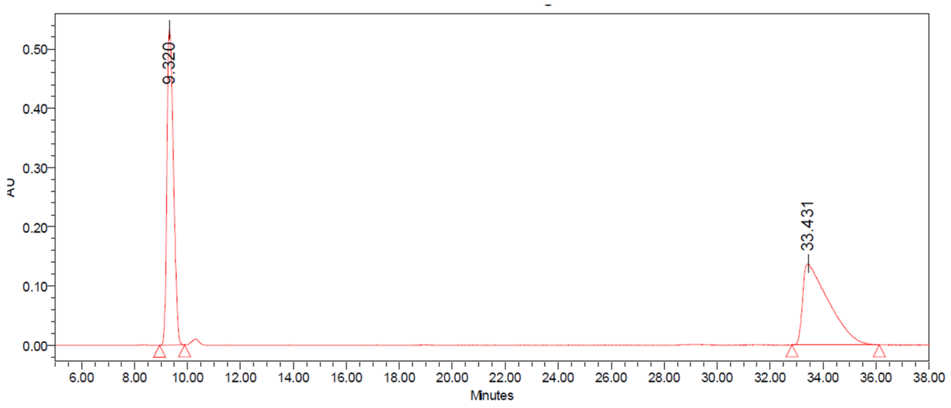

Peak Results

	RT	Height	Area	% Area
1	9.320	532226	9137536	49.55
2	33.431	137032	9303319	50.45

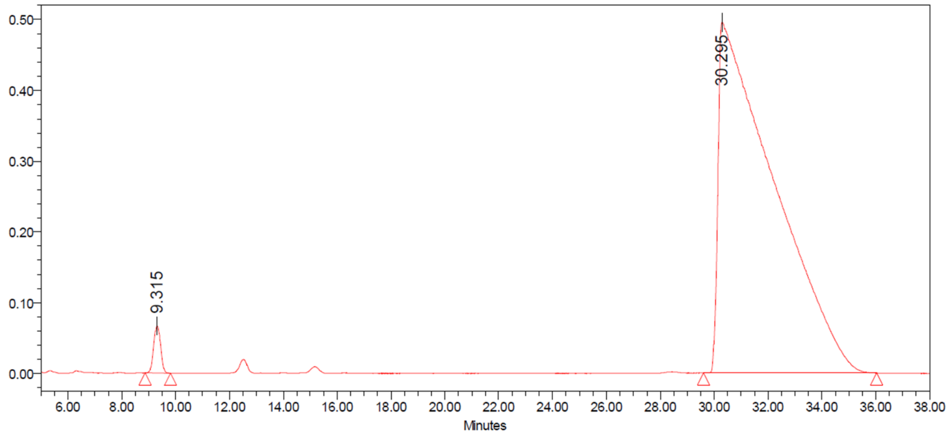

Peak Results

	RT	Height	Area	% Area
1	9.315	66587	1198633	1.65
2	30.295	495062	71457741	98.35

Supplementary Figure 53. UPCC traces of *rac*-**3a** (top) and enantioenriched-**3a** (bottom).

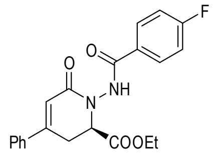

3b

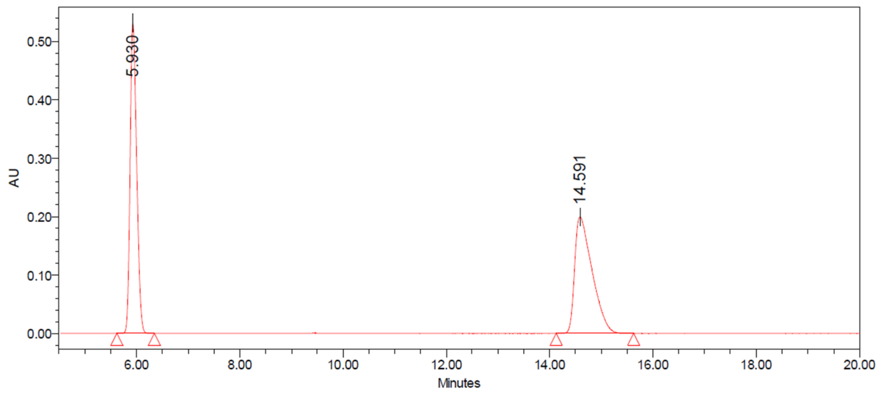

Peak Results

	RT	Height	Area	% Area
1	5.930	531298	4686023	50.03
2	14.591	199582	4680340	49.97

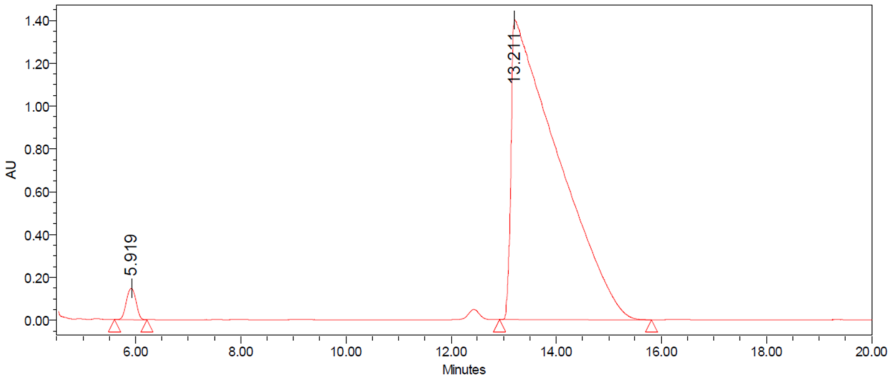

Peak Results

	RT	Height	Area	% Area
1	5.919	145586	1868462	2.09
2	13.211	1399660	87524412	97.91

Supplementary Figure 54. UPCC traces of *rac*-**3b** (top) and enantioenriched-**3b** (bottom).

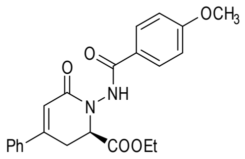

3c

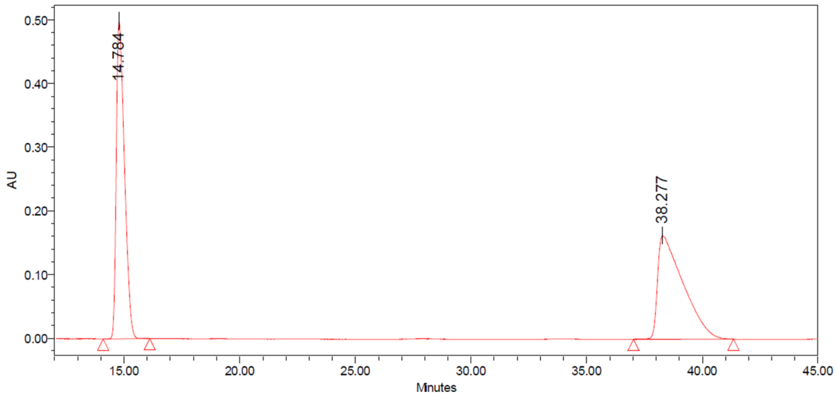

Peak Results

	RT	Height	Area	% Area
1	14.784	498851	12341375	49.81
2	38.277	163168	12437795	50.19

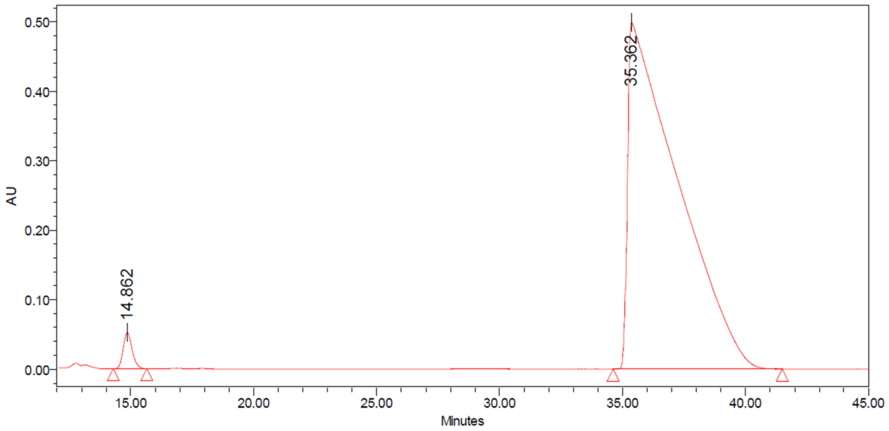

Peak Results

	RT	Height	Area	% Area
1	14.862	52071	1323171	1.84
2	35.362	498597	70783685	98.16

Supplementary Figure 55. UPCC traces of *rac*-**3c** (top) and enantioenriched-**3c** (bottom).

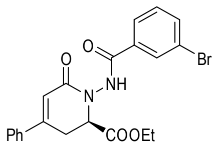

3d

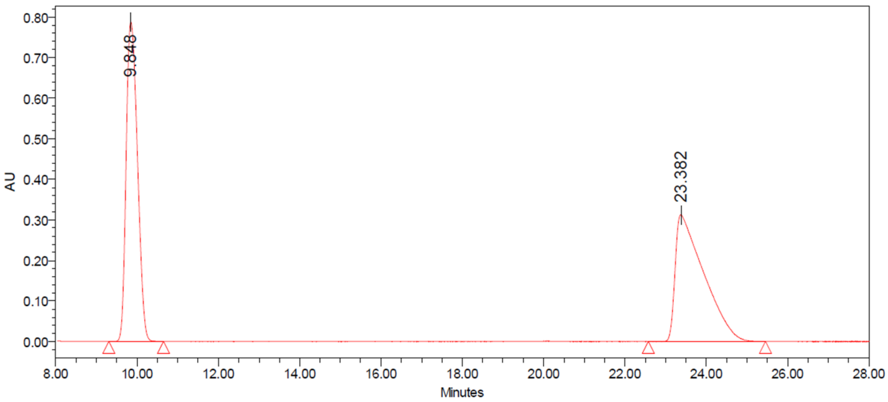

Peak Results

	RT	Area	Height	% Area
1	9.848	15246976	787430	49.98
2	23.382	15259793	312117	50.02

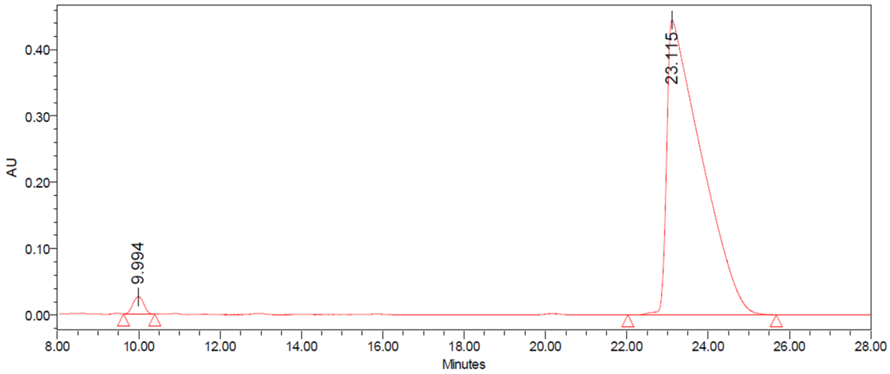

Peak Results

	RT	Area	Height	% Area
1	9.994	486452	26145	1.81
2	23.115	26355013	444693	98.19

Supplementary Figure 56. UPCC traces of *rac*-**3d** (top) and enantioenriched-**3d** (bottom).

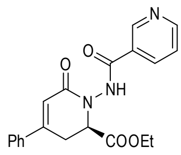

3e

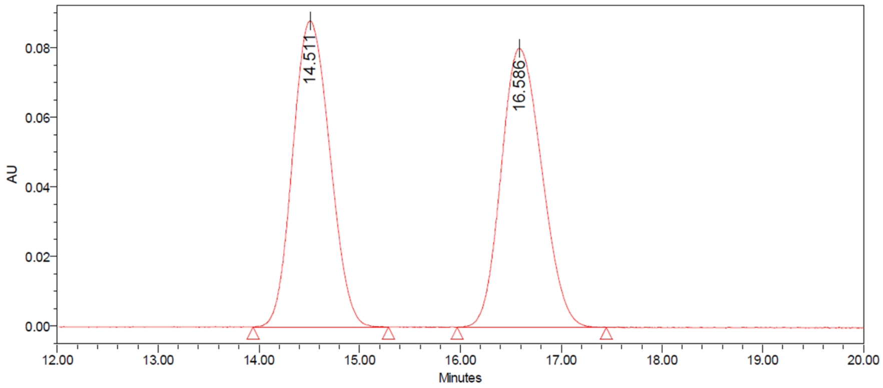

Peak Results

	RT	Area	Height	% Area
1	14.511	2251750	88032	50.03
2	16.586	2248632	80240	49.97

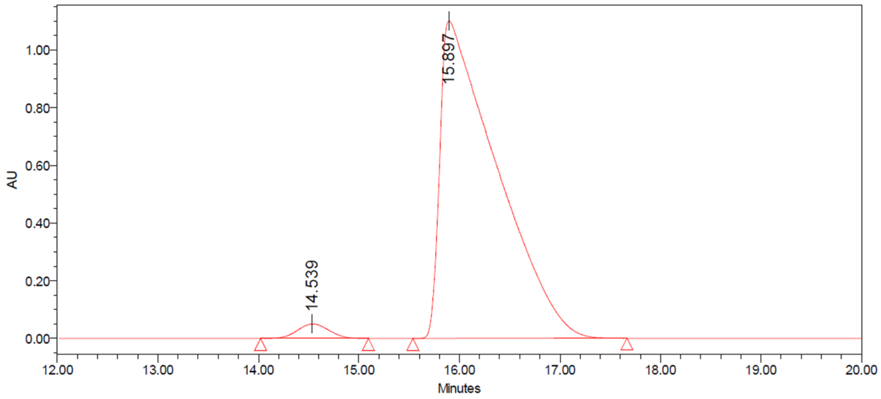

Peak Results

	RT	Area	Height	% Area
1	14.539	1091498	49844	2.45
2	15.897	43408485	1099789	97.55

Supplementary Figure 57. UPCC traces of *rac*-**3e** (top) and enantioenriched-**3e** (bottom).

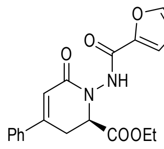

3f

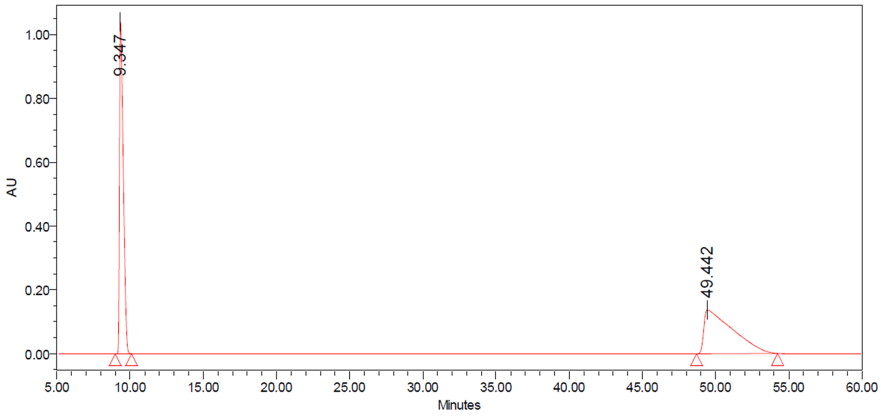

Peak Results

	RT	Height	Area	% Area
1	9.347	1040077	18673480	50.02
2	49.442	137134	18656374	49.98

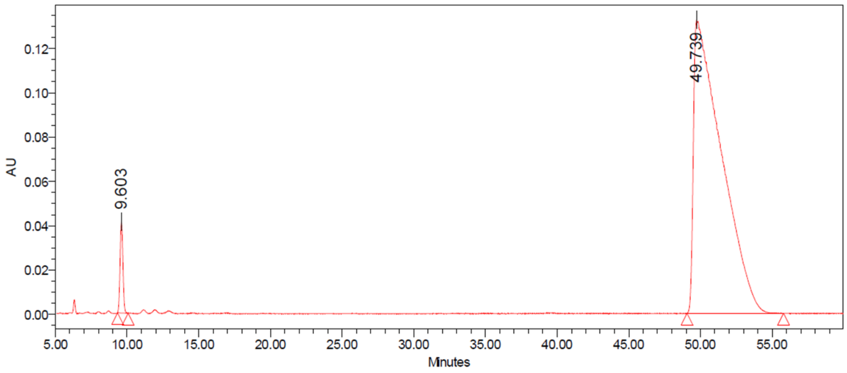

Peak Results

	RT	Height	Area	% Area
1	9.603	41152	568703	3.08
2	49.739	132204	17884472	96.92

Supplementary Figure 58. UPCC traces of *rac*-**3f** (top) and enantioenriched-**3f** (bottom).

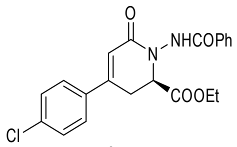

3g

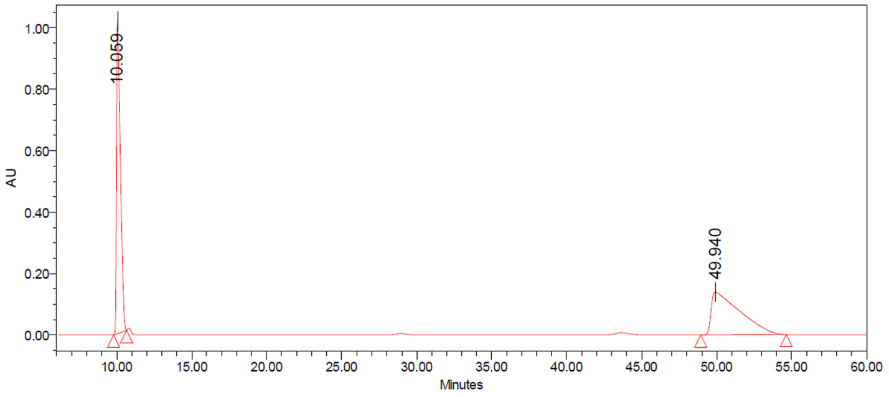

Peak Results

	RT	Height	Area	% Area
1	10.059	1017806	18268391	49.66
2	49.940	140405	18520524	50.34

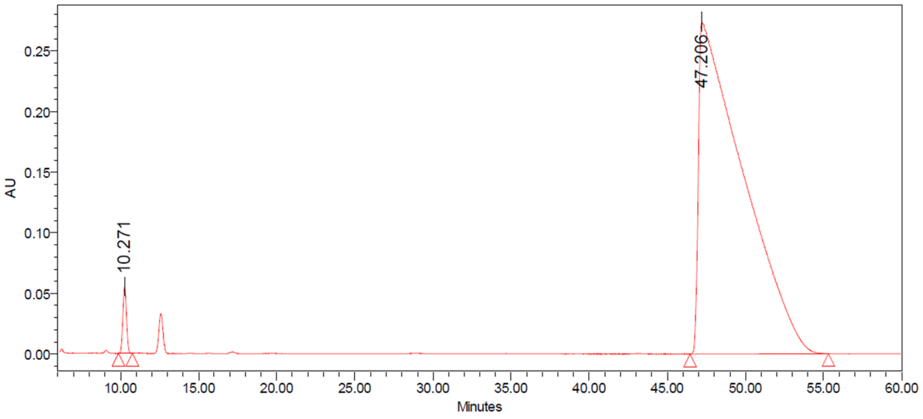

Peak Results

	RT	Height	Area	% Area
1	10.271	55159	891038	1.61
2	47.206	274217	54516529	98.39

Supplementary Figure 59. UPCC traces of *rac*-**3g** (top) and enantioenriched-**3g** (bottom).

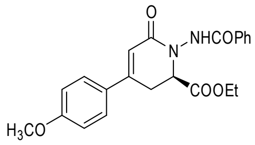

3h

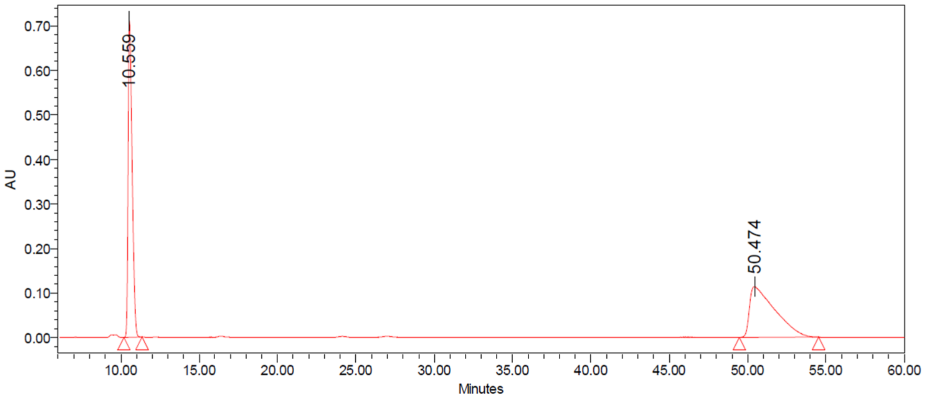

Peak Results

	RT	Height	Area	% Area
1	10.559	709824	12687129	50.00
2	50.474	113499	12685100	50.00

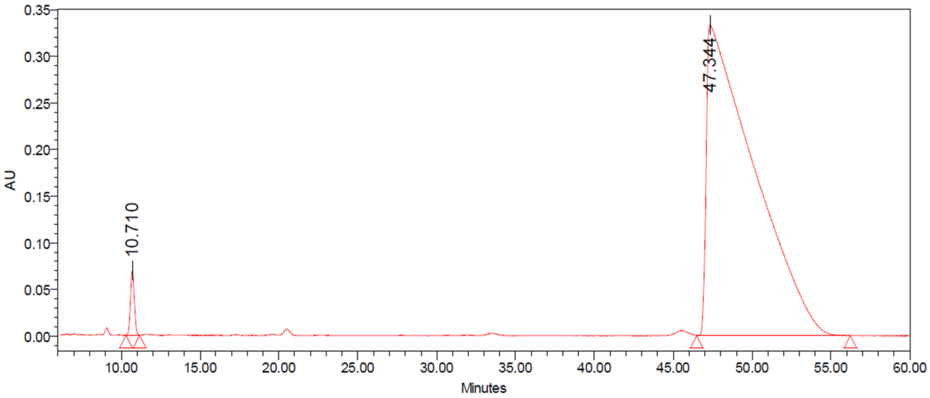

Peak Results

	RT	Height	Area	% Area
1	10.710	69408	1181953	1.66
2	47.344	333039	69921665	98.34

Supplementary Figure 60. UPCC traces of *rac*-**3h** (top) and enantioenriched-**3h** (bottom).

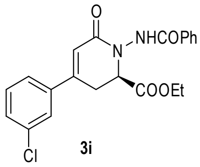

3i

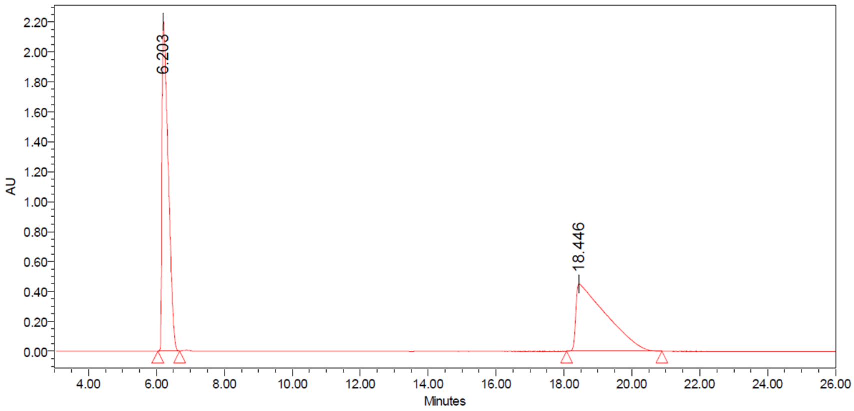

Peak Results

	RT	Area	Height	% Area
1	6.203	27579731	2200970	49.84
2	18.446	27756007	449276	50.16

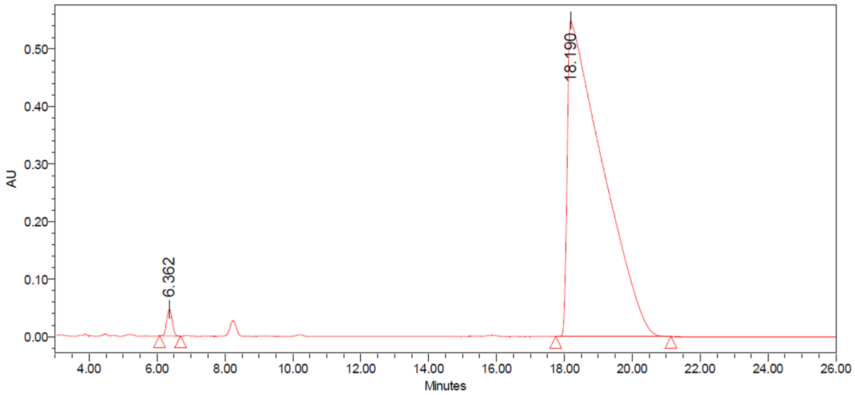

Peak Results

	RT	Area	Height	% Area
1	6.362	515745	45944	1.29
2	18.190	39335798	548561	98.71

Supplementary Figure 61. UPCC traces of *rac*-**3i** (top) and enantioenriched-**3i** (bottom).

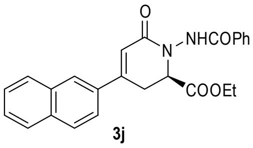

3j

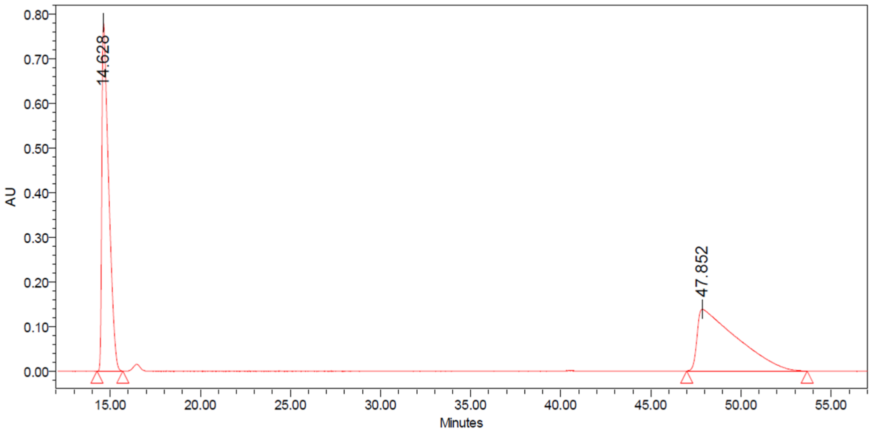

Peak Results

	RT	Height	Area	% Area
1	14.628	780525	20641999	49.56
2	47.852	138239	21006994	50.44

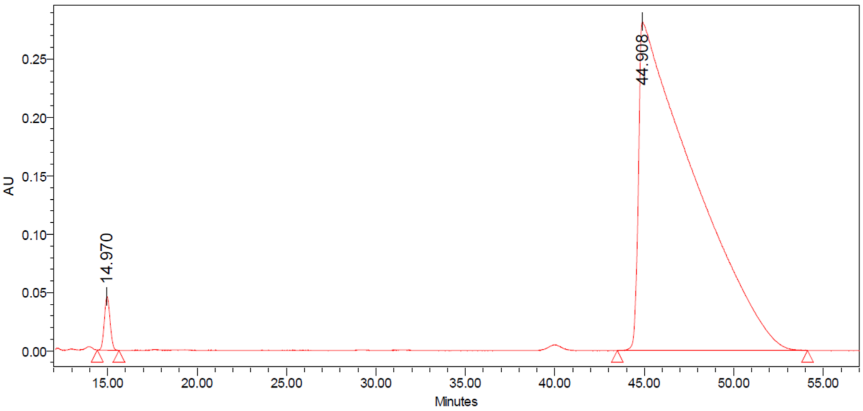

Peak Results

	RT	Height	Area	% Area
1	14.970	45997	1024454	1.66
2	44.908	281505	60821505	98.34

Supplementary Figure 62. UPCC traces of *rac*-**3j** (top) and enantioenriched-**3j** (bottom).

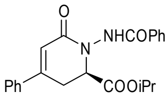

3k

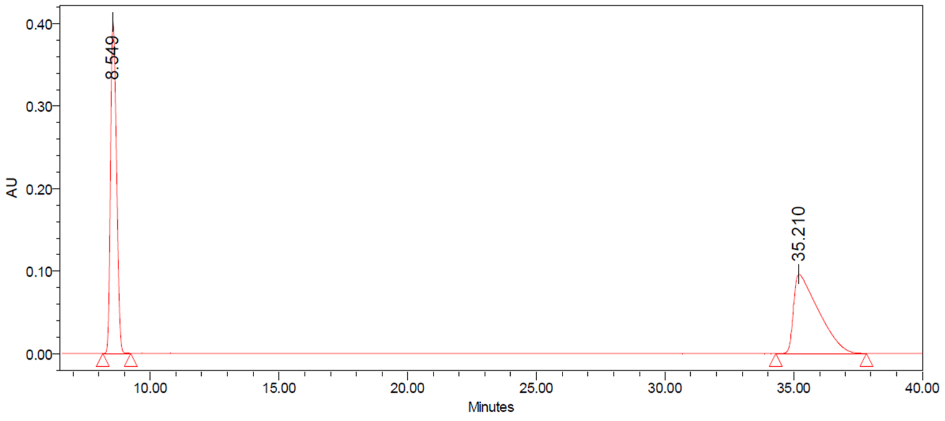

Peak Results

	RT	Area	Height	% Area
1	8.549	6507888	401879	50.12
2	35.210	6475609	96006	49.88

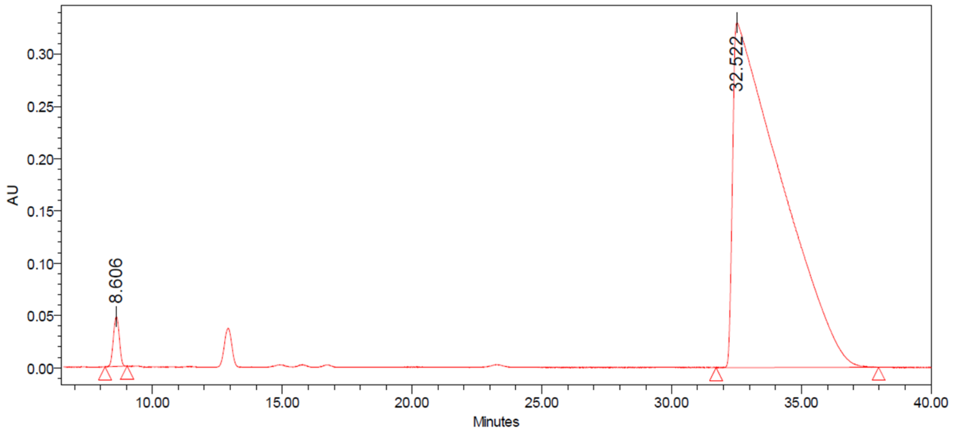

Peak Results

	RT	Area	Height	% Area
1	8.606	759395	47685	1.74
2	32.522	42782449	329780	98.26

Supplementary Figure 63. UPCC traces of *rac*-**3k** (top) and enantioenriched-**3k** (bottom).

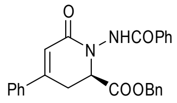

3l

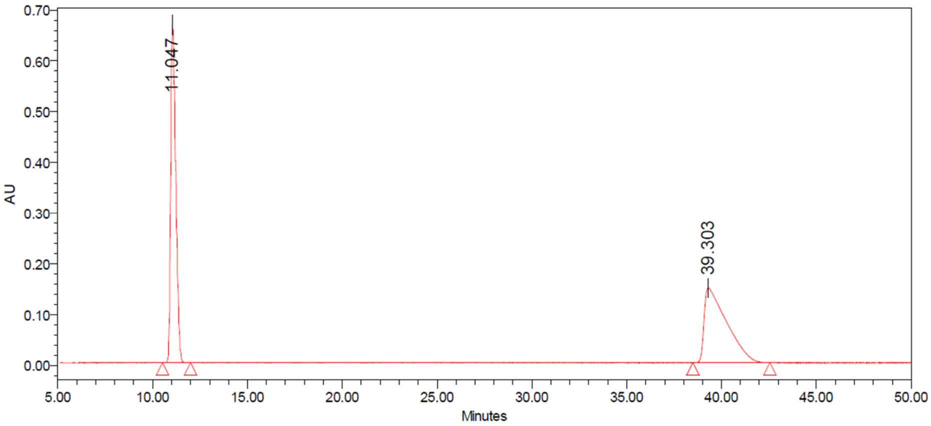

Peak Results

	RT	Area	Height	% Area
1	11.047	12246708	665272	50.07
2	39.303	12213277	147047	49.93

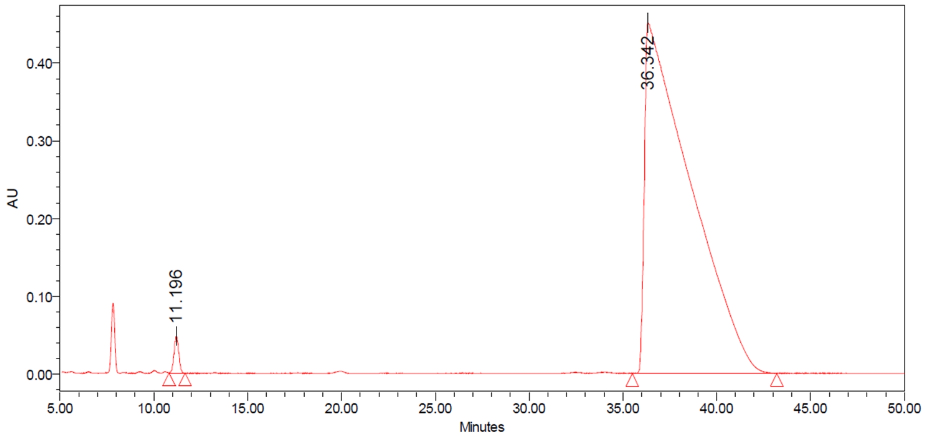

Peak Results

	RT	Area	Height	% Area
1	11.196	817662	47242	1.06
2	36.342	76499536	450392	98.94

Supplementary Figure 64. UPCC traces of *rac*-**3l** (top) and enantioenriched-**3l** (bottom).

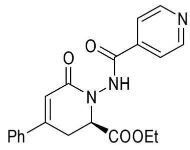

3m

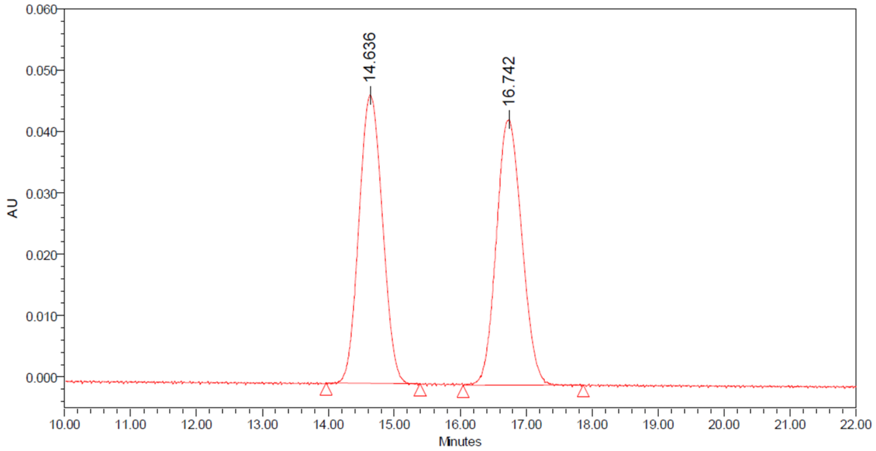

Peak Results

	RT	Area	Height	% Area
1	14.636	1159894	47002	49.83
2	16.742	1167677	43352	50.17

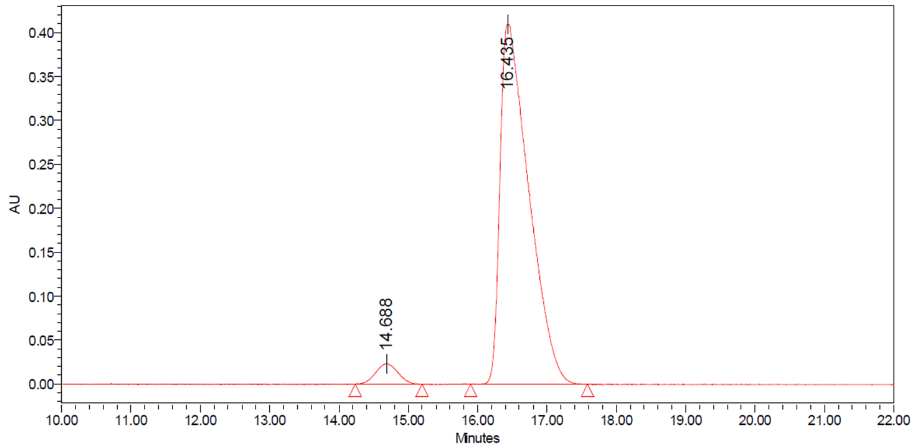

Peak Results

	RT	Area	Height	% Area
1	14.688	518470	23301	4.23
2	16.435	11745835	409917	95.77

Supplementary Figure 65. UPCC traces of *rac*-**3m** (top) and enantioenriched-**3m** (bottom).

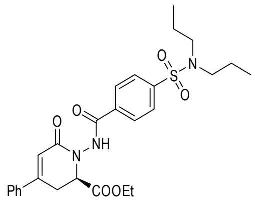

3n

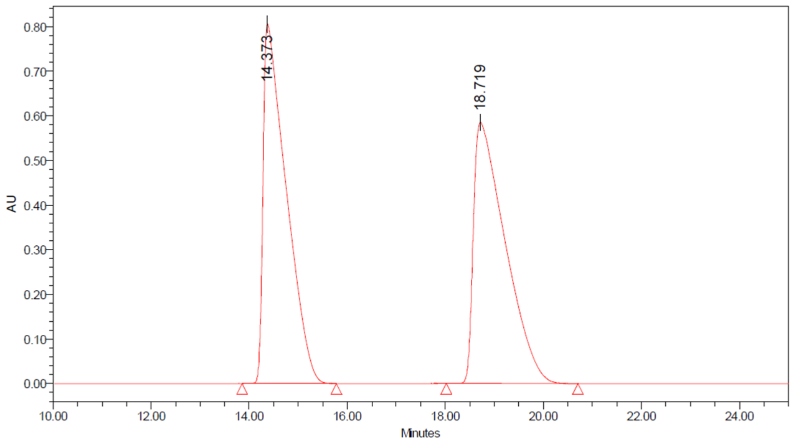

Peak Results

	RT	Area	Height	% Area
1	14.373	26313996	805139	50.01
2	18.719	26302806	584988	49.99

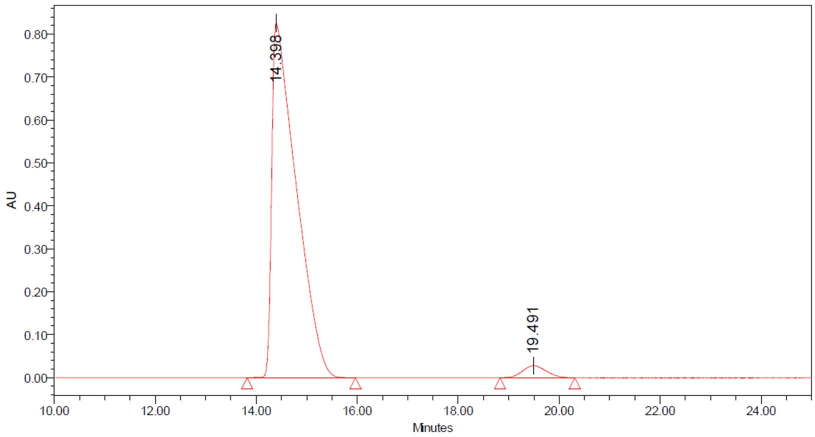

Peak Results

	RT	Area	Height	% Area
1	14.398	27774731	825651	96.80
2	19.491	917523	28020	3.20

Supplementary Figure 66. UPCC traces of *rac*-**3n** (top) and enantioenriched-**3n** (bottom).

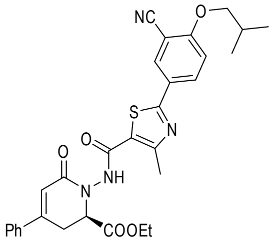

3o

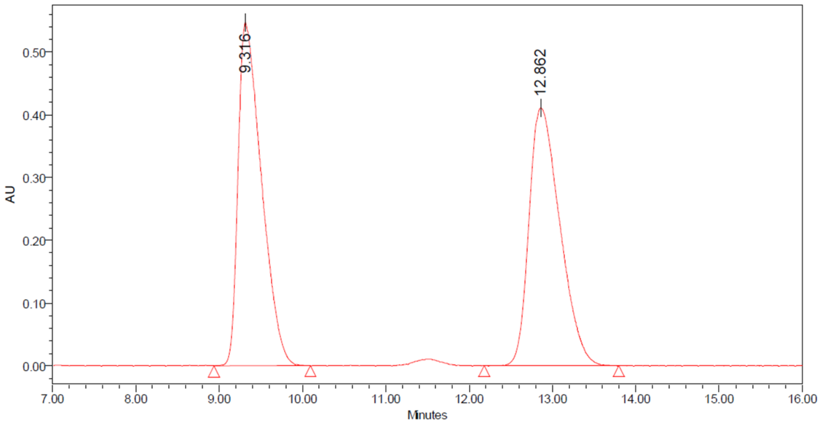

Peak Results

	RT	Area	Height	% Area
1	9.316	10619780	547146	50.07
2	12.862	10589904	410447	49.93

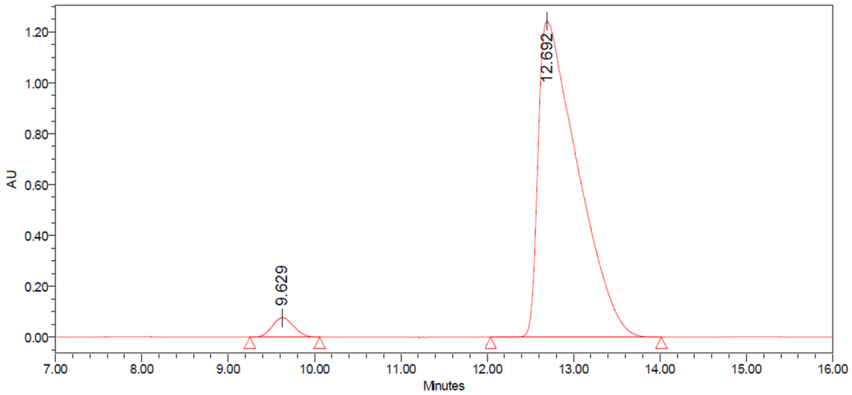

Peak Results

	RT	Area	Height	% Area
1	9.629	1320398	76388	3.23
2	12.692	39548958	1241573	96.77

Supplementary Figure 67. UPCC traces of *rac*-**3o** (top) and enantioenriched-**3o** (bottom).

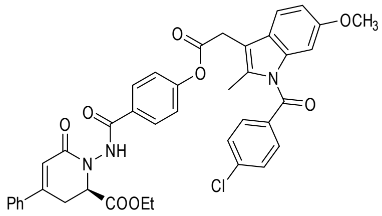

3p

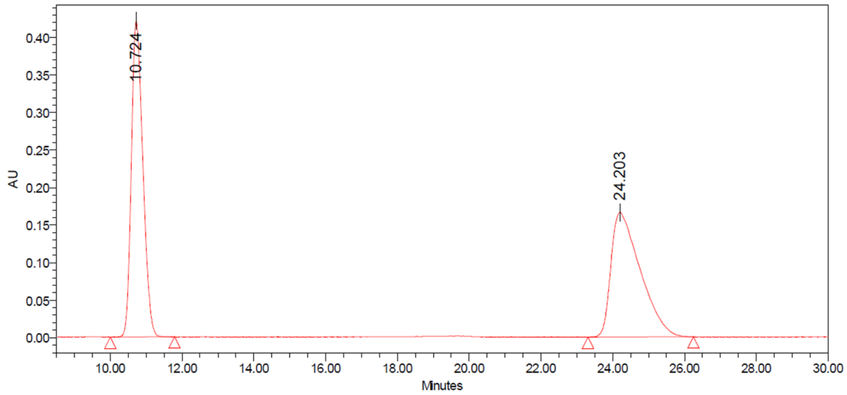

Peak Results

	RT	Area	Height	% Area
1	10.724	9384404	420633	49.90
2	24.203	9420316	165926	50.10

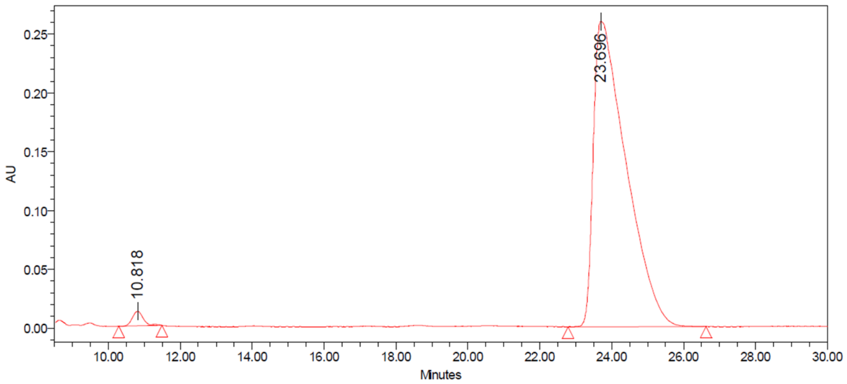

Peak Results

	RT	Area	Height	% Area
1	10.818	267424	12366	1.60
2	23.696	16399826	259734	98.40

Supplementary Figure 68. UPCC traces of *rac*-**3p** (top) and enantioenriched-**3p** (bottom).

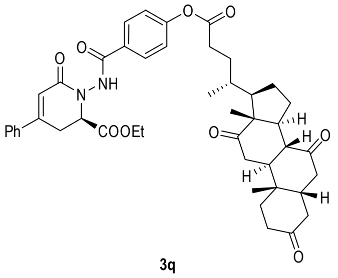

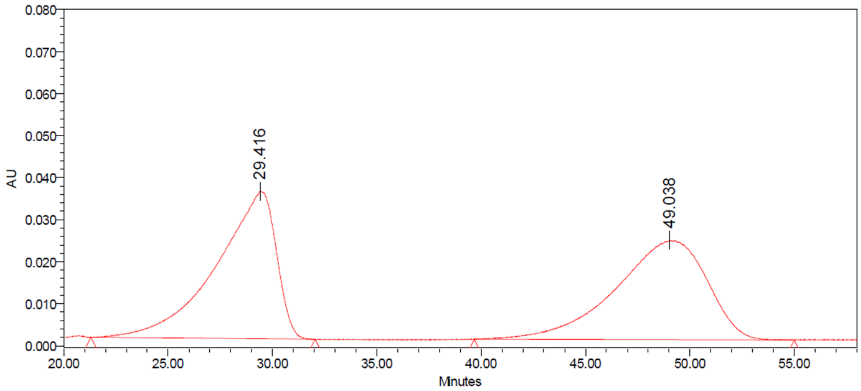

Peak Results

	RT	Area	Height	% Area
1	29.416	6971002	35052	49.41
2	49.038	7137987	23588	50.59

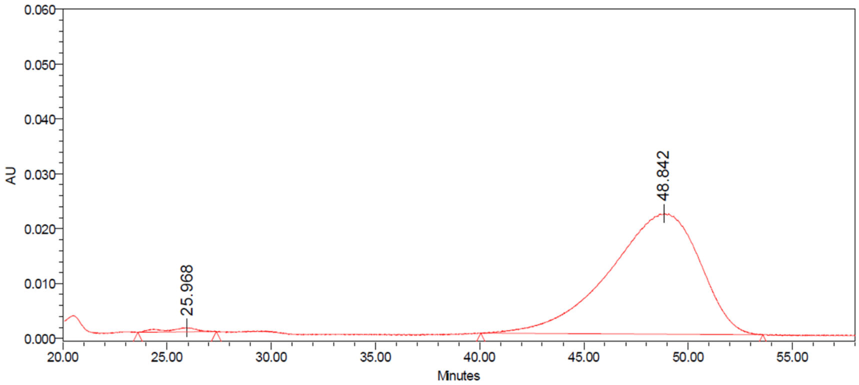

Peak Results

	RT	Area	Height	% Area
1	25.968	79894	756	1.19
2	48.842	6649549	22034	98.81

Supplementary Figure 69. UPCC traces of *rac*-**3q** (top) and enantioenriched-**3q** (bottom).

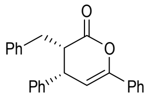

9a

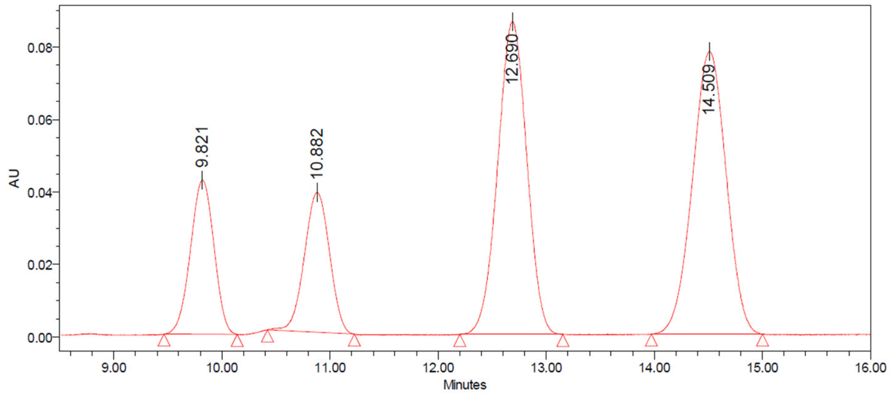

	RT	Area	Height	% Area
1	9.821	645755	42593	14.11
2	10.882	625673	38603	13.67
3	12.690	1610455	86319	35.19
4	14.509	1694048	77890	37.02

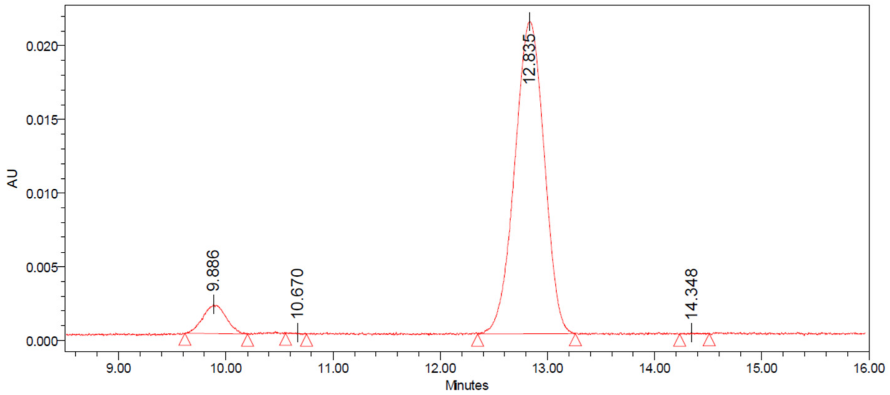

	RT	Area	Height	% Area
1	9.886	29272	2009	6.74
2	10.670	236	79	0.05
3	12.835	404614	21220	93.12
4	14.348	405	81	0.09

Supplementary Figure 70. UPCC traces of *rac*-**9a** (top) and enantioenriched-**9a** (bottom).

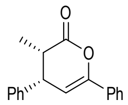

9b

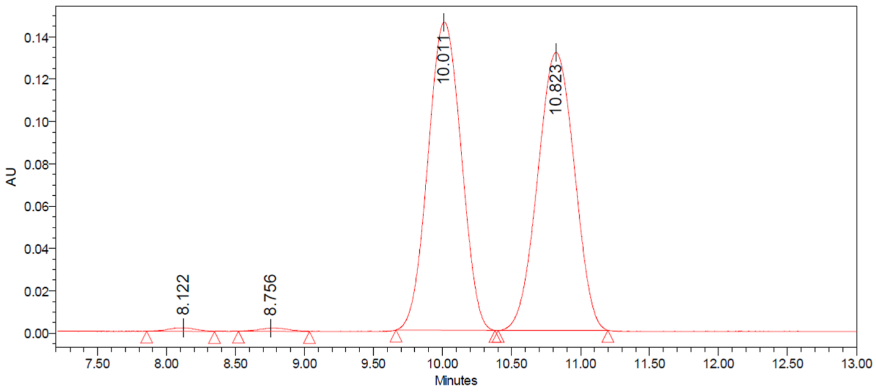

	RT	Area	Height	% Area
1	8.122	21798	1674	0.44
2	8.756	22480	1584	0.46
3	10.011	2443087	145574	49.68
4	10.823	2430536	131349	49.42

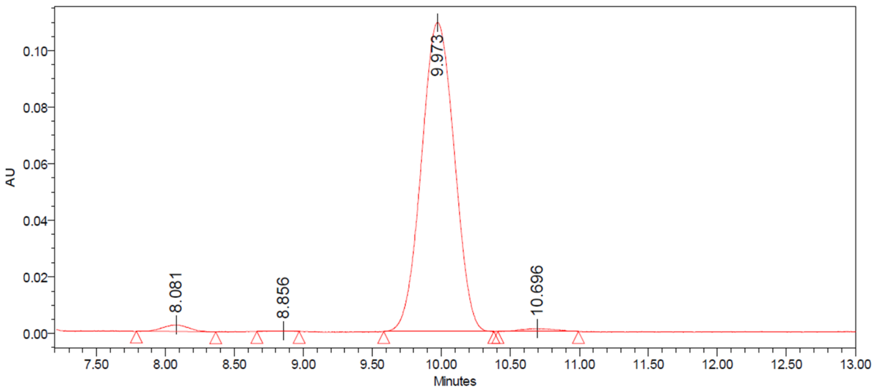

	RT	Area	Height	% Area
1	8.081	30267	2331	1.63
2	8.856	1936	212	0.10
3	9.973	1811696	109310	97.31
4	10.696	17795	1016	0.96

Supplementary Figure 71. UPCC traces of *rac*-**9b** (top) and enantioenriched-**9b** (bottom).

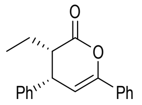

9c

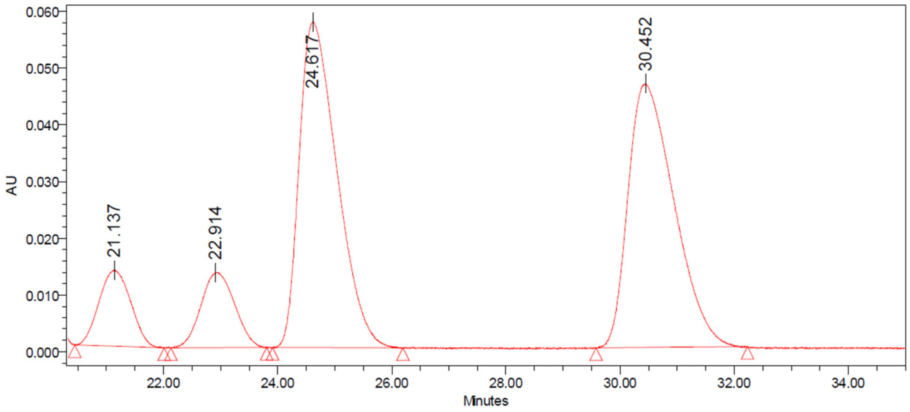

	RT	Area	Height	% Area
1	21.137	516995	13361	8.22
2	22.914	541806	13224	8.61
3	24.617	2624235	57415	41.73
4	30.452	2606293	46522	41.44

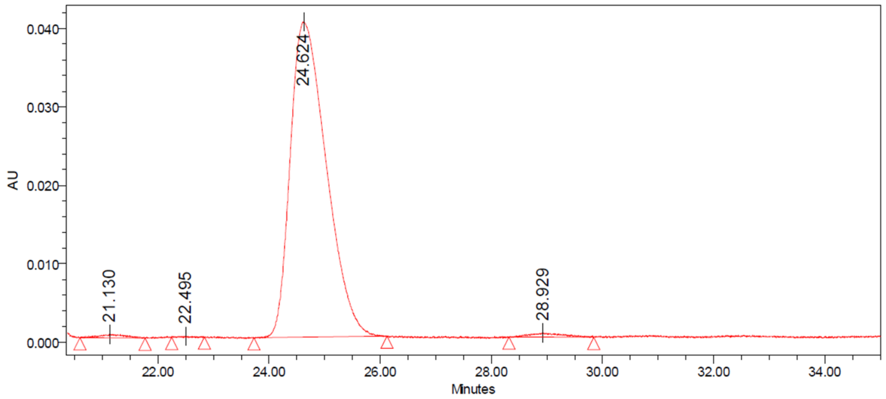

	RT	Area	Height	% Area
1	21.130	15226	472	0.83
2	22.495	1511	120	0.08
3	24.624	1802168	40222	97.94
4	28.929	21225	547	1.15

Supplementary Figure 72. UPCC traces of *rac*-**9c** (top) and enantioenriched-**9c** (bottom).

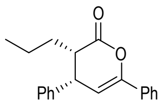

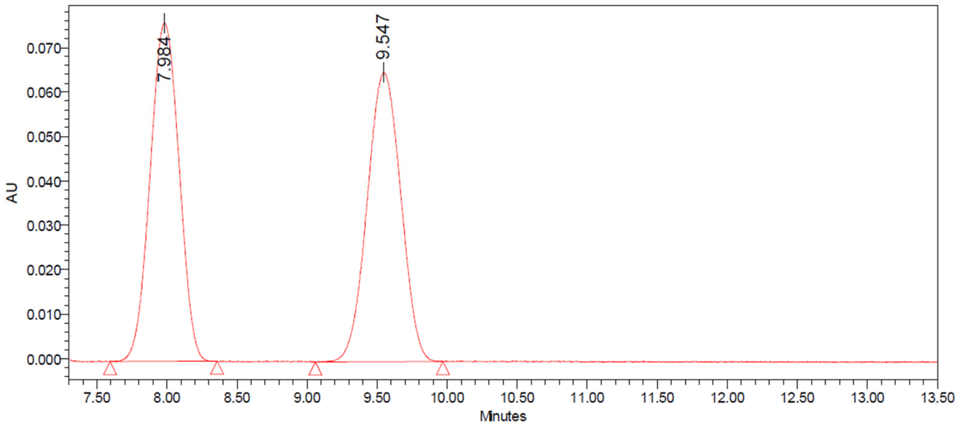

	RT	Area	Height	% Area
1	7.984	1088846	76141	50.00
2	9.547	1088720	65038	50.00

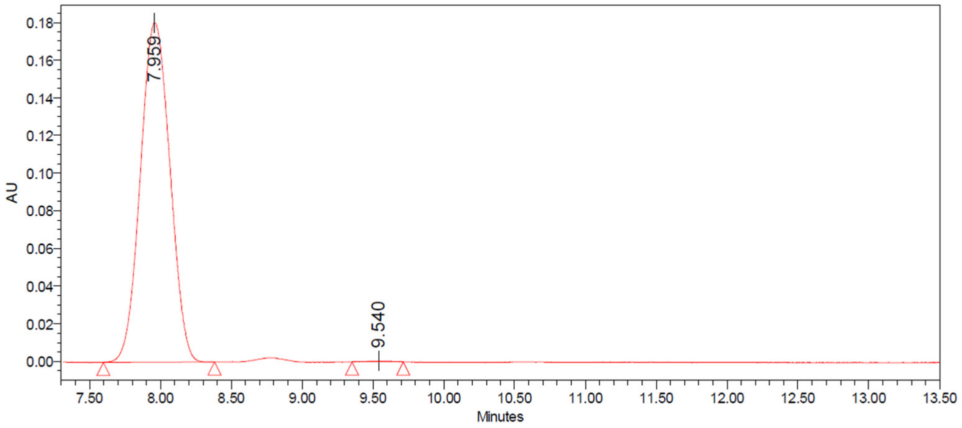

	RT	Area	Height	% Area
1	7.959	2609560	180256	99.76
2	9.540	6396	555	0.24

Supplementary Figure 73. UPCC traces of *rac*-**9d** (top) and enantioenriched-**9d** (bottom).

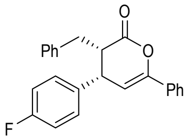

9e

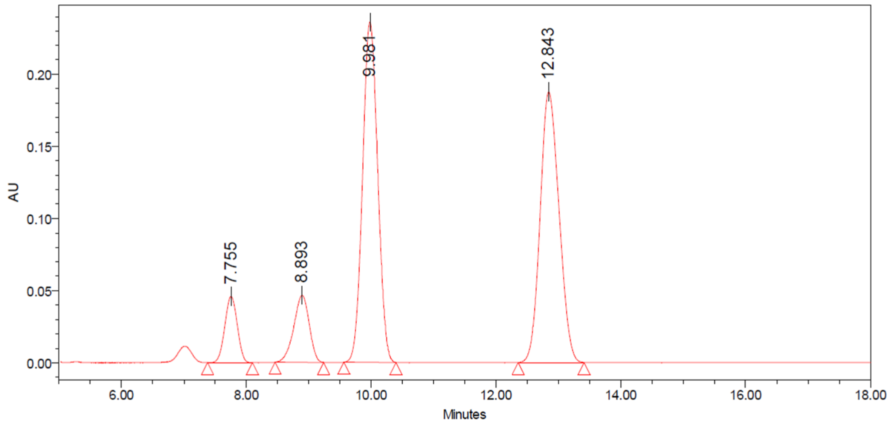

	RT	Area	Height	% Area
1	7.755	640478	45942	6.84
2	8.893	799861	46441	8.54
3	9.981	3968642	235887	42.38
4	12.843	3955087	187514	42.24

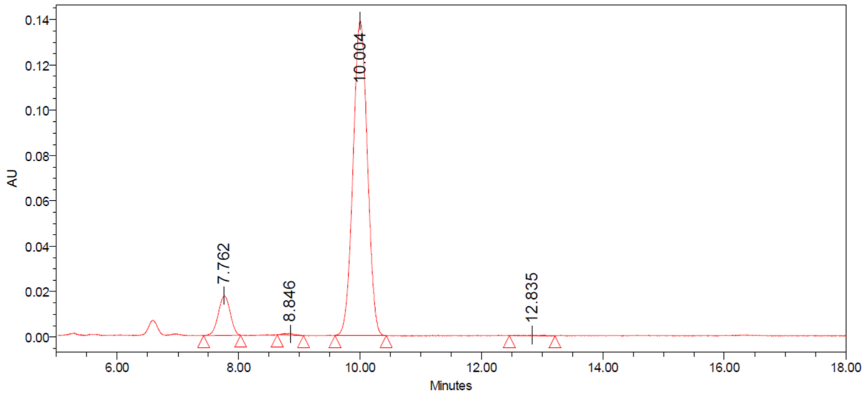

	RT	Area	Height	% Area
1	7.762	236273	17397	9.33
2	8.846	9384	665	0.37
3	10.004	2280405	138843	90.09
4	12.835	5276	262	0.21

Supplementary Figure 74. UPCC traces of *rac*-**9e** (top) and enantioenriched-**9e** (bottom).

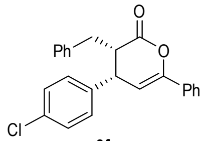

9f

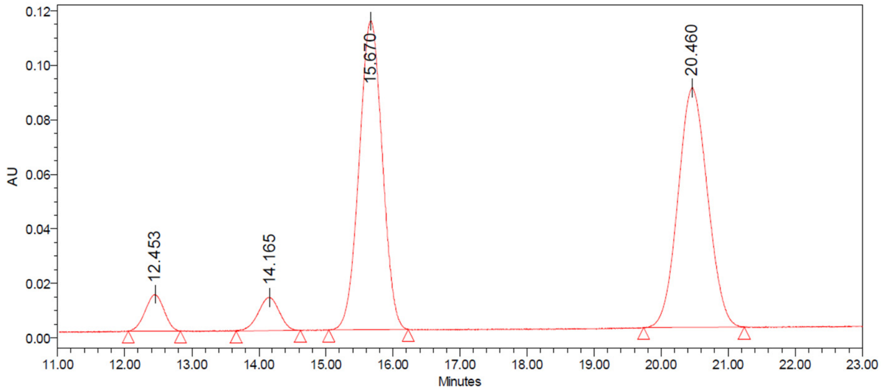

	RT	Area	Height	% Area
1	12.453	256950	13395	4.33
2	14.165	264587	12192	4.45
3	15.670	2718595	113301	45.76
4	20.460	2700332	87829	45.46

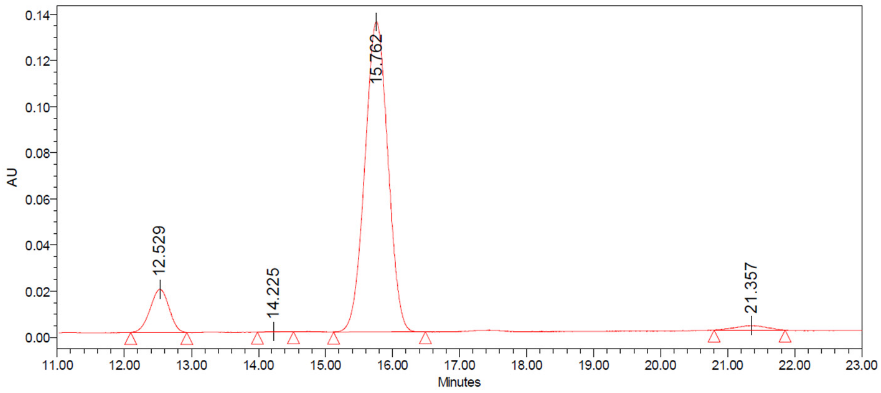

	RT	Area	Height	% Area
1	12.529	361958	18668	9.97
2	14.225	2096	168	0.06
3	15.762	3204908	134606	88.32
4	21.357	59717	1901	1.65

Supplementary Figure 75. UPCC traces of *rac*-**9f** (top) and enantioenriched-**9f** (bottom).

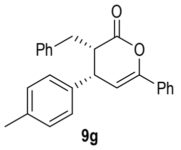

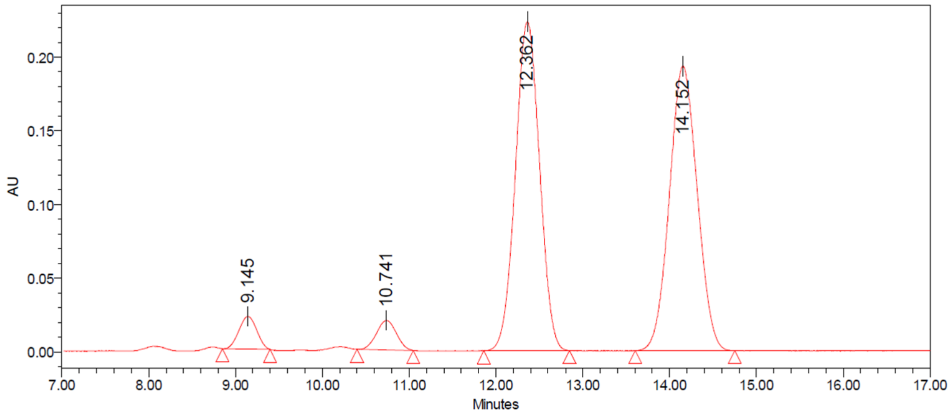

	RT	Area	Height	% Area
1	9.145	317349	22242	3.44
2	10.741	319855	20129	3.47
3	12.362	4303863	223324	46.66
4	14.152	4281845	193230	46.43

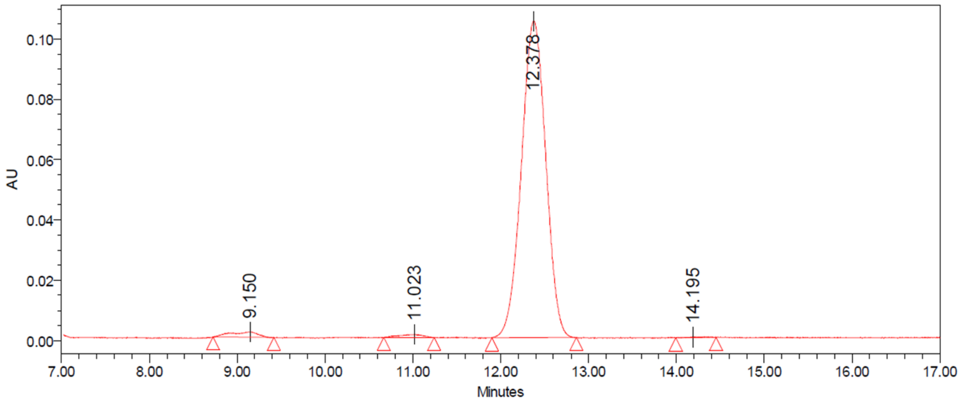

	RT	Area	Height	% Area
1	9.150	38862	1767	1.87
2	11.023	18507	1054	0.89
3	12.378	2018528	105012	97.13
4	14.195	2299	184	0.11

Supplementary Figure 76. UPCC traces of *rac*-**9g** (top) and enantioenriched-**9g** (bottom).

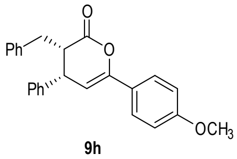

9h

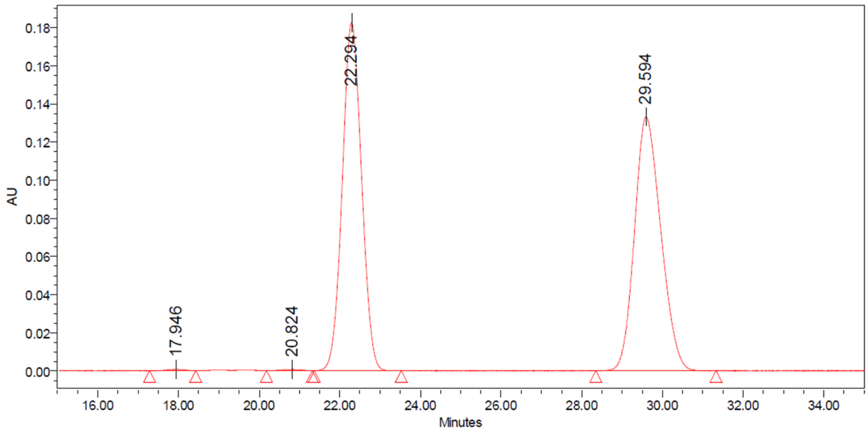

Peak Results

	Name	RT	Area	Height	% Area
1		17.946	17441	663	0.14
2		20.824	16733	595	0.14
3		22.294	6046024	182358	49.90
4		29.594	6035621	133151	49.82

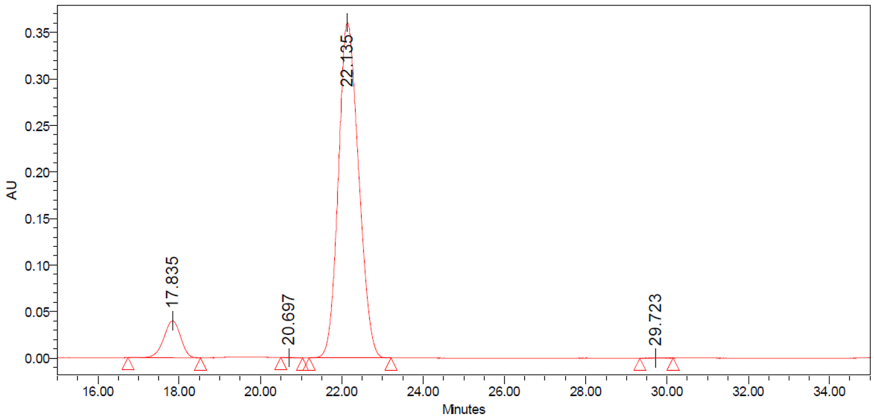

Peak Results

	RT	Area	Height	% Area
1	17.835	1139030	39646	8.42
2	20.697	708	80	0.01
3	22.135	12381607	360155	91.56
4	29.723	2243	127	0.02

Supplementary Figure 77. UPCC traces of *rac*-**9h** (top) and enantioenriched-**9h** (bottom).

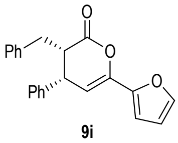

9i

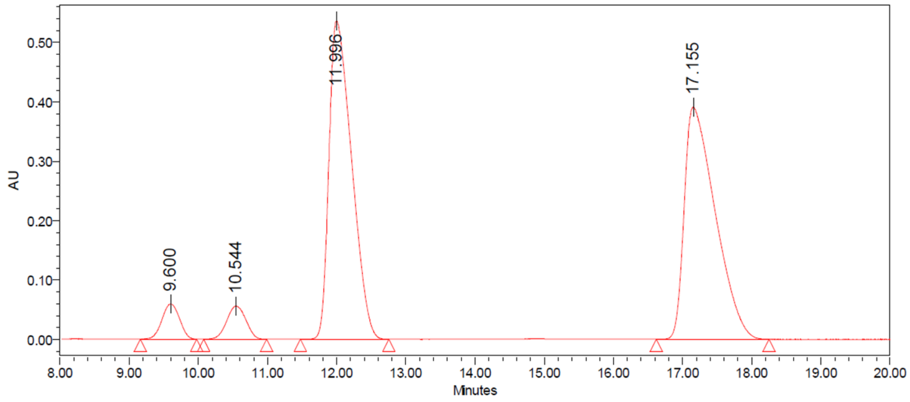

Peak Results

	RT	Area	Height	% Area
1	9.600	1045409	59260	3.96
2	10.544	1064225	55646	4.03
3	11.996	12148771	535725	46.02
4	17.155	12141849	390651	45.99

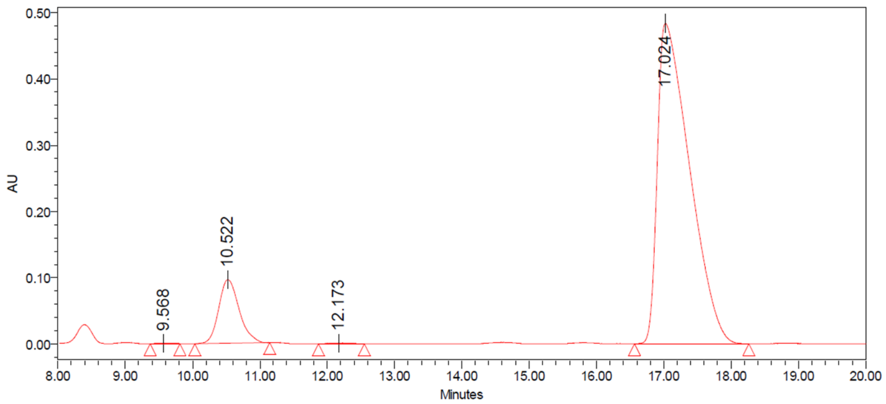

Peak Results

	RT	Area	Height	% Area
1	9.568	9514	714	0.05
2	10.522	2030161	95867	11.20
3	12.173	15176	816	0.08
4	17.024	16068840	483107	88.66

Supplementary Figure 78. UPCC traces of *rac*-**9i** (top) and enantioenriched-**9i** (bottom).

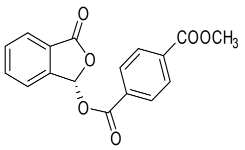

12a

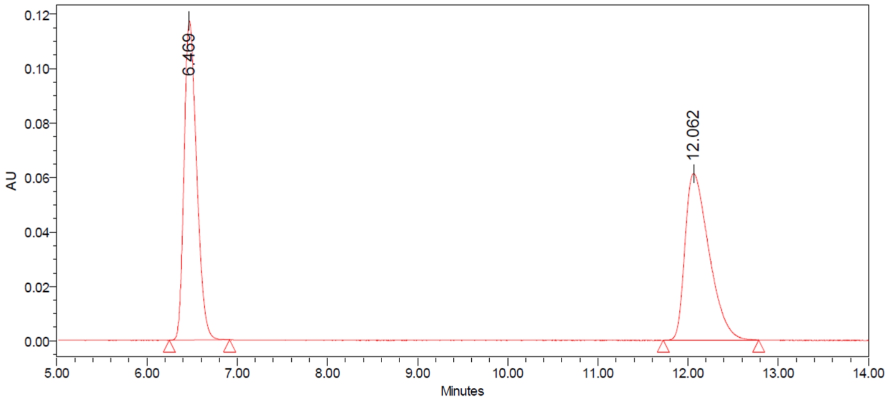

Peak Results

	RT	Area	Height	% Area
1	6.469	1136079	117295	50.01
2	12.062	1135787	61220	49.99

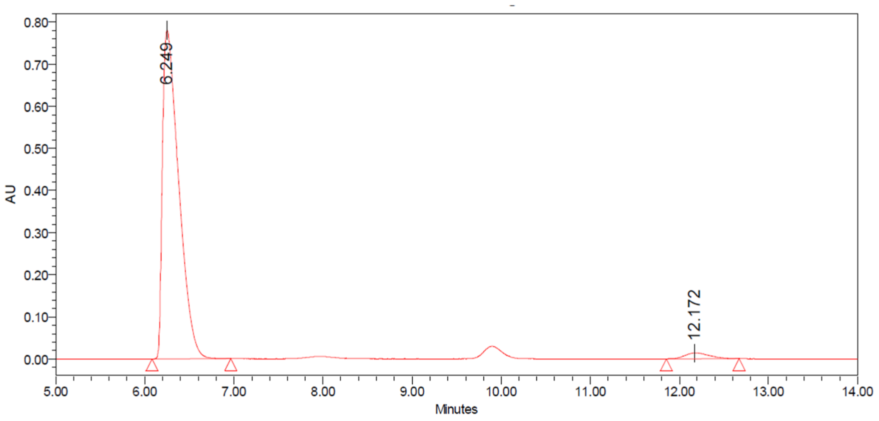

Peak Results

	RT	Area	Height	% Area
1	6.249	9851029	780265	97.38
2	12.172	264566	13814	2.62

Supplementary Figure 79. UPCC traces of *rac*-**12a** (top) and enantioenriched-**12a** (bottom).

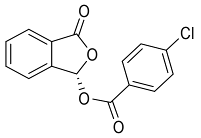

12b

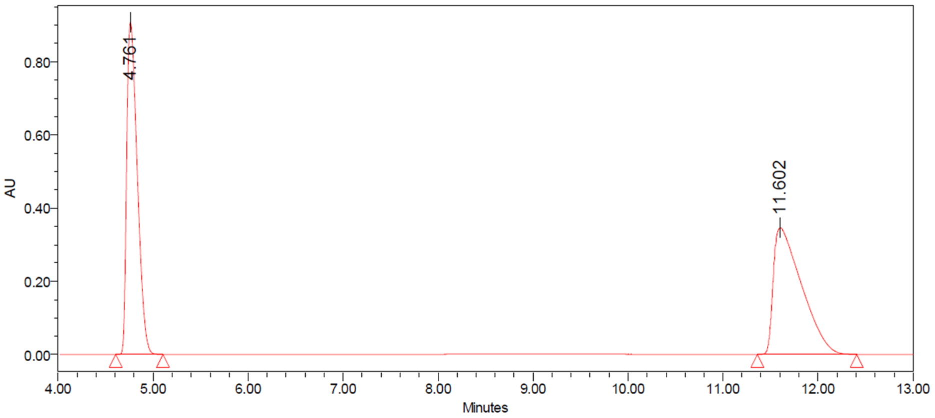

Peak Results

	RT	Area	Height	% Area
1	4.761	7035698	908148	49.65
2	11.602	7135935	346201	50.35

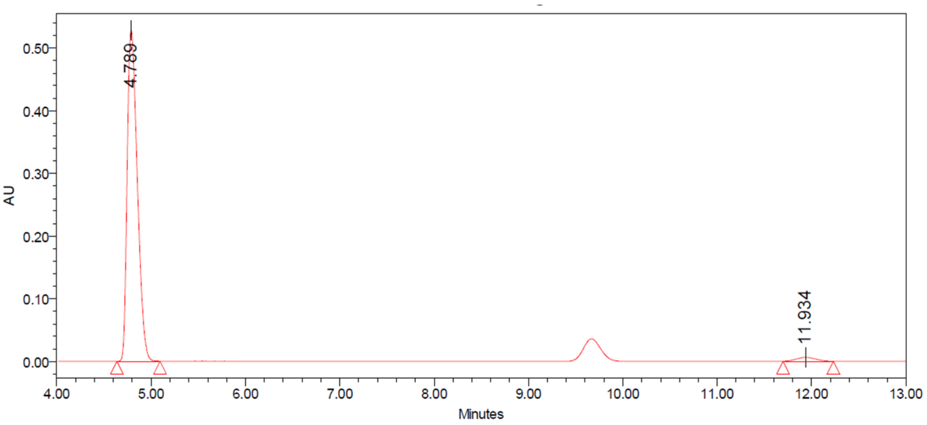

Peak Results

	RT	Area	Height	% Area
1	4.789	3945250	528010	97.86
2	11.934	86263	6085	2.14

Supplementary Figure 80. UPCC traces of *rac*-**12b** (top) and enantioenriched-**12b** (bottom).

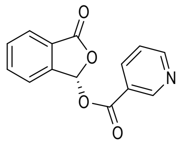

12c

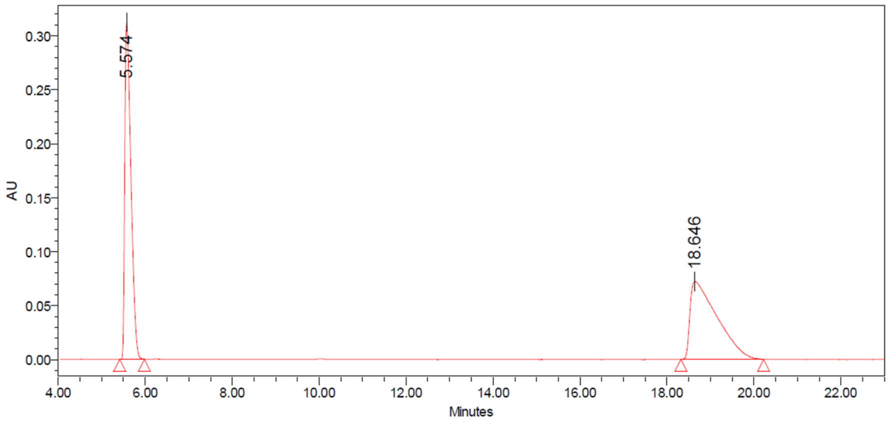

Peak Results

	RT	Area	Height	% Area
1	5.574	3089883	312182	49.70
2	18.646	3126959	72139	50.30

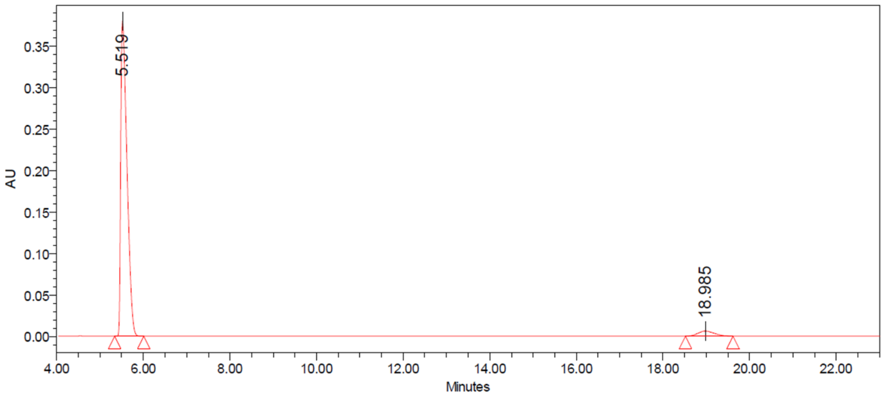

Peak Results

	RT	Area	Height	% Area
1	5.519	3811157	380106	95.93
2	18.985	161571	6116	4.07

Supplementary Figure 81. UPCC traces of *rac*-**12c** (top) and enantioenriched-**12c** (bottom).

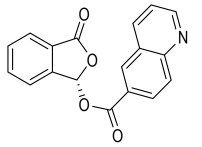

12d

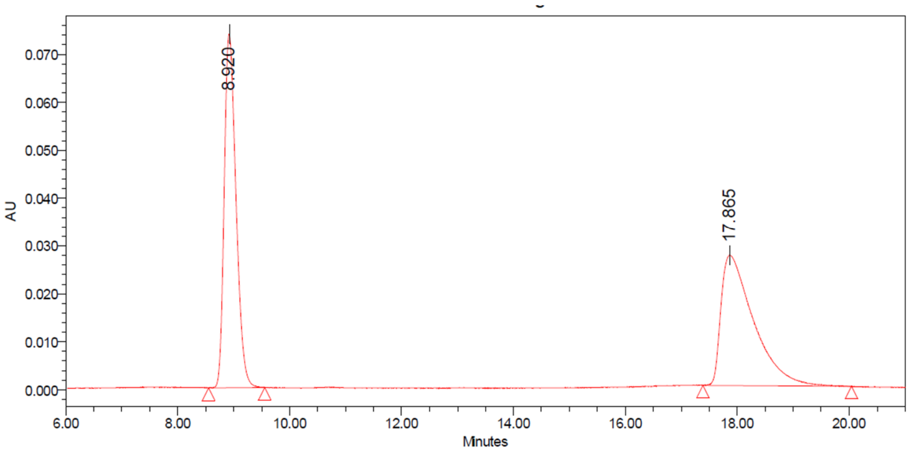

Peak Results

	RT	Area	Height	% Area
1	8.920	1069157	73791	49.33
2	17.865	1098065	27139	50.67

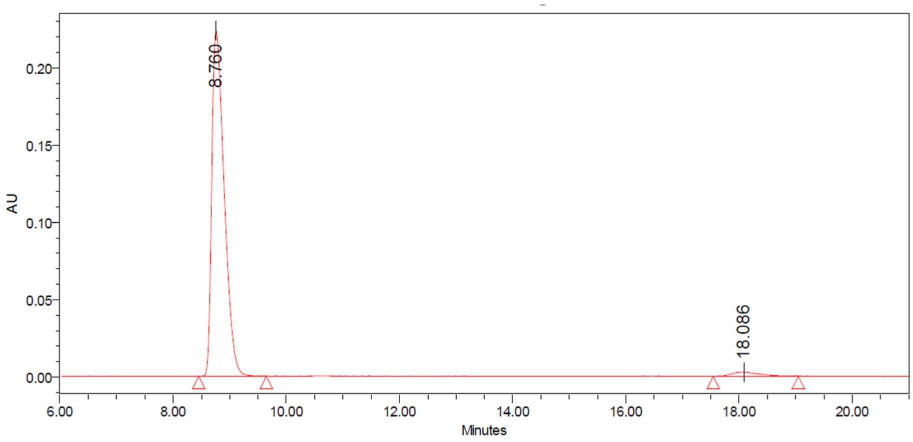

Peak Results

	RT	Area	Height	% Area
1	8.760	3427855	223550	97.49
2	18.086	88229	2621	2.51

Supplementary Figure 82. UPCC traces of *rac*-**12d** (top) and enantioenriched-**12d** (bottom).

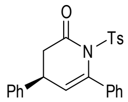

16

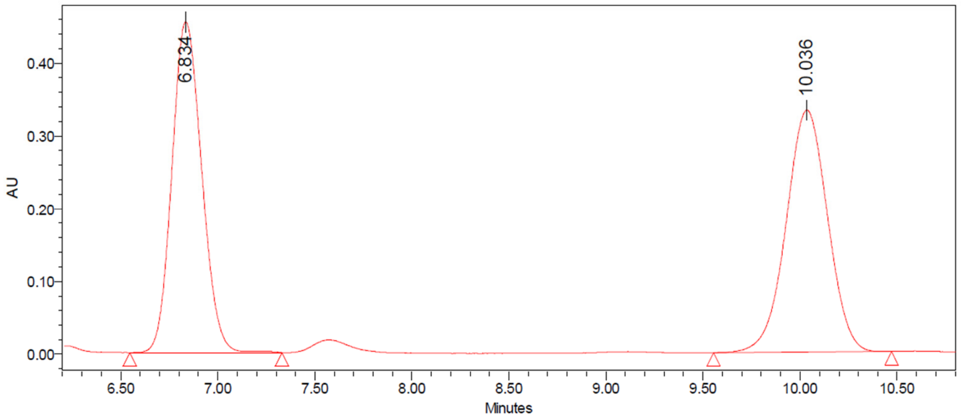

Peak Results

	RT	Area	Height	% Area
1	6.834	4835972	454107	49.51
2	10.036	4930744	332584	50.49

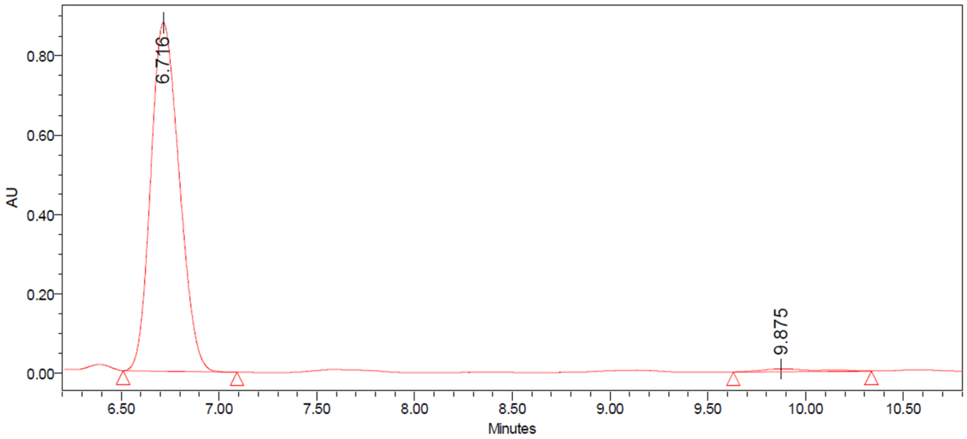

Peak Results

	RT	Area	Height	% Area
1	6.716	8888817	878680	98.42
2	9.875	142388	7103	1.58

Supplementary Figure 83. UPCC traces of *rac*-**16** (top) and enantioenriched-**16** (bottom).

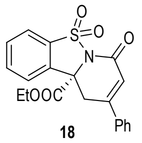

18

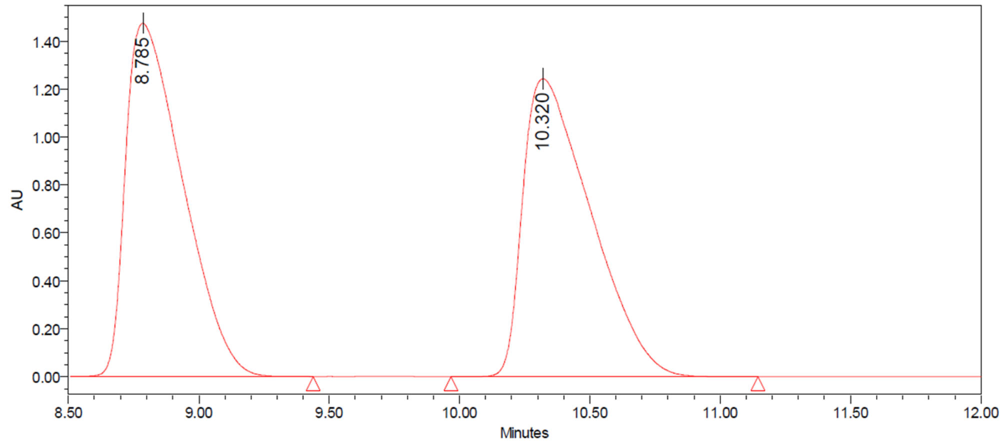

Peak Results

	RT	Area	Height	% Area
1	8.785	22476558	1474737	49.96
2	10.320	22515333	1242805	50.04

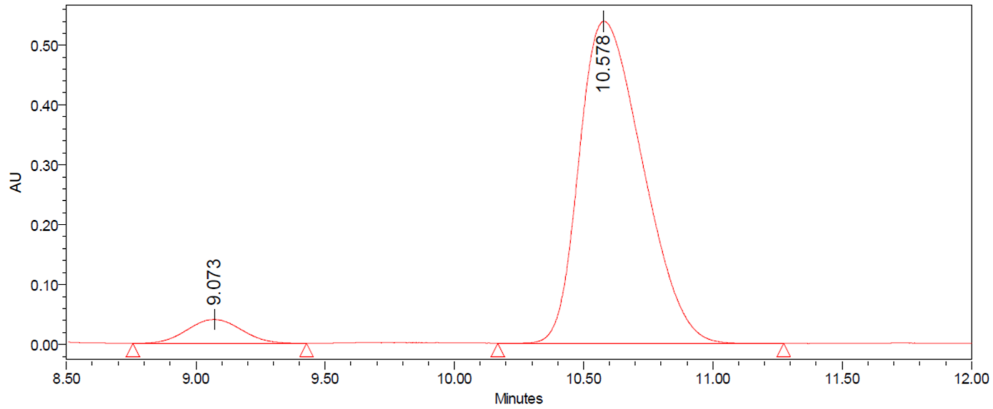

Peak Results

	RT	Area	Height	% Area
1	9.073	577641	39656	5.92
2	10.578	9175044	537940	94.08

Supplementary Figure 84. UPCC traces of *rac*-**18** (top) and enantioenriched-**18** (bottom).

4. Supplementary Reference

1. Mauleo´n, P., Krinsky, J. L. & Toste, F. D. Mechanistic studies on Au(I)-catalyzed [3,3]-sigmatropic rearrangements using cyclopropane probes. *J. Am. Chem. Soc.* **131**, 4513–4520 (2009).
2. Sone, Y., Kimura, Y., Ota, R., Mochizuki, T., Ito, J. & Nishii, Y. Catalytic hydrogenolysis of enantioenriched donor-acceptor cyclopropanes using H_2 and palladium on charcoal. *Eur. J. Org. Chem.* **2017**, 2842–2847 (2017).
3. Debad, J. D., Morris, J. C., Magnus, P. & Bard, A. J. Anodic coupling of diphenylbenzo[*k*]fluoranthene: Mechanistic and kinetic studies utilizing cyclic voltammetry and electrogenerated chemiluminescence. *J. Org. Chem.* **62**, 530–537 (1997).
4. Xu, J., Jin, Z. & Chi, R. Y. Organocatalytic enantioselective γ-aminoalkylation of unsaturated ester: Access to pipecolic acid derivatives. *Org. Lett.* **15**, 5028–5031 (2013).
5. Jia, W.-Q., Zhang, H.-M., Zhang, C.-L., Gao, Z.-H. & Ye, S. N-Heterocyclic carbene-catalyzed [4+2] annulation of α,β-unsaturated carboxylic acids: enantioselective synthesis of dihydropyridinones and spirocyclic oxindolodihydropyridinones. *Org. Chem. Front.* **3**, 77–81 (2016).
6. Wu, X.*, et al.* Polyhalides as efficient and mild oxidants for oxidative carbene organocatalysis by radical processes. *Angew. Chem. Int. Ed*. **56**, 2942–2946 (2017).
7. De Sarkar, S. & Studer, A. NHC-catalyzed Michael addition to α,β-unsaturated aldehydes by redox activation. *Angew. Chem. Int. Ed.* **49**, 9266–9269 (2010).
8. Yetra, S. R., Bhunia, A., Patra, A., Mane, M. V., Vanka, K. & Biju, A. T. Enantioselective N-heterocyclic carbene-catalyzed annulations of 2-bromoenals with 1,3-dicarbonyl compounds and enamines via chiral α,β-unsaturated acylazoliums. *Adv. Synth. Catal.* **355**, 1089–1097 (2013).
9. Mo, J., Shen, L. & Chi, Y. R. Direct β-activation of saturated aldehydes to formal Michael acceptors through oxidative NHC catalysis. *Angew. Chem. Int. Ed.* **52**, 8588–8591 (2013).
10. Mo, J., Yang, R., Chen, X., Tiwari, B. & Chi, Y. R. Direct α-functionalization of simple aldehydes via oxidative N-heterocyclic carbene catalysis. *Org. Lett.* **15**, 50–53 (2013).
11. Zhao, X., Ruhl, K. E. & Rovis, T. N-heterocyclic-carbene-catalyzed asymmetric oxidative hetero-Diels-Alder reactions with simple aliphatic aldehydes. *Angew. Chem. Int. Ed.* **51**, 12330–12333 (2012).
12. Liu, Y.*, et al.* Carbene-catalyzed dynamic kinetic resolution and asymmetric acylation of hydroxyphthalides and related natural products. *Angew. Chem. Int. Ed.* **59**, 3859–3863 (2020).
13. Liu, Y.*, et al.* Catalytic asymmetric acetalization of carboxylic acids for access to chiral phthalidyl ester prodrugs. *Nat. Commun.* **10**, 1675 (2019).
14. Zhu, T., Mou, C., Li, B., Smetankova, M., Song, B. A. & Chi, Y. R. N-heterocyclic carbene-catalyzed δ-carbon LUMO activation of unsaturated aldehydes. *J. Am. Chem. Soc.* **137**, 5658–5661 (2015).
15. Cheng, J., Huang, Z. & Chi, Y. R. NHC organocatalytic formal LUMO activation of α,β-unsaturated esters for reaction with enamides. *Angew. Chem. Int. Ed.* **52**, 8592–8596 (2013).
16. Zheng, P. C.*, et al.* Oxidative N-heterocyclic carbene-catalyzed γ-carbon addition of enals to imines: Mechanistic studies and access to antimicrobial compounds. *Chem. Eur. J.* **21**, 9984–9987 (2015).
17. Squitieri, R. A., Fitzpatrick, K. P., Jaworski, A. A. & Scheidt, K. A. Synthesis and evaluation of azolium-based halogen-bond donors. *Chem. Eur. J.* **25**, 10069–10073 (2019).
18. Chan, Y. C. & Yeung, Y. Y. Halogen bond catalyzed bromocarbocyclization. *Angew. Chem. Int. Ed.* **57**,

3483–3487 (2018).
